# Single-walled carbon-nanohorns improve biocompatibility over nanotubes by triggering less protein-initiated pyroptosis and apoptosis in macrophages

**DOI:** 10.1038/s41467-018-04700-z

**Published:** 2018-06-19

**Authors:** Bing He, Yujie Shi, Yanqin Liang, Anpu Yang, Zhipu Fan, Lan Yuan, Xiajuan Zou, Xin Chang, Hua Zhang, Xueqing Wang, Wenbin Dai, Yiguang Wang, Qiang Zhang

**Affiliations:** 1 Department of Pharmaceutics, School of Pharmaceutical Sciences, Peking University, Beijing, 100191 China; 2 State Key Laboratory of Natural and Biomimetic Drugs, Beijing, 100191 China; 3 Beijing Key Laboratory of Molecular Pharmaceutics and New Drug Delivery Systems, School of Pharmaceutical Sciences, Peking University, Beijing, 100191 China; 4 Department of Chemical Biology, School of Pharmaceutical Sciences, Peking University, Beijing, 100191 China; 5 Centre of Medical and Health Analysis, Peking University, Beijing, 100191 China

## Abstract

Single-walled carbon-nanohorns (SNH) exhibit huge application prospects. Notably, spherical SNH possess different morphology from conventional carbon nanotubes (CNT). However, there is a tremendous lack of studies on the nanotoxicity and mechanism of SNH, and their comparison with nanotubes. Here, the dissimilarity between SNH and CNT is found in many aspects including necrosis, pyroptosis, apoptosis, protein expression, hydrolases leakage, lysosome stress, membrane disturbance and the interaction with membrane proteins. The improved biocompatibility of SNH over four types of established CNT is clearly demonstrated in macrophages. Importantly, a key transmembrane protein, glycoprotein nonmetastatic melanoma protein B (GPNMB) is discovered to initiate the nanotoxicity. Compared to CNT, the weaker nano-GPNMB interaction in SNH group induces lower degree of cascade actions from nano/membrane interplay to final cell hypotoxicity. In conclusion, the geometry of single-construct unit, but not that of dispersive forms or intracellular levels of nanocarbons make the most difference.

## Abstract (web summary)

Carbon-nanohorns have a unique morphology and structure yet little is known about the biocompatibility. Here, the authors investigate the biocompatibility and bio-interaction of carbon nanohorns, compare them to carbon nanotubes and show the superior biocompatibility and safety of the nanohorns.

## Introduction

Single-walled carbon-nanohorns (SNH) are a new form of nanocarbons that have attracted increasing attentions due to their unique morphology and structure. A single nanohorn consists of a cone of sp^2^-bonded carbon atoms with 2–5 nm in diameter and 40–80 nm in length^1,2^. Hundreds of nanohorn monomers assemble during synthesis to form globular aggregate. Owing to the similarity in atomic arrangement, carbon nanotubes (CNT) are the closest structural analog of SNH. In fact, SNH inherit the physicochemical features of CNT, and exhibit great potentials in energy conversion, chemical engineering, catalysts, fuel cell and electronic applications^3,4^.

Very importantly, SNH may possess superiority over CNT in biomedical applications due to its structure characteristics, no metal catalyst used during synthesis and their mass production at room temperature^5^. Currently, SNH have been reported broadly as a multi-functional platform for drug delivery^6^, in vivo photoacoustic imaging^7^, photothermal therapy^8^ and so forth^9^. However, there is a tremendous lack of studies on the nanosafety and biocompatibility of SNH, not to mention its molecular mechanism. On the other hand, SNH exhibit great difference in morphology from CNT. For cannular CNT, the aspect ratios (or length/wide ratios) as the key factor significantly affect their nanotoxicity^10^, while SNH are approximately isotropic in three dimensions owing to the spherical morphology. Therefore, it is very essential to compare these two types of nanocarbons in terms of their nanosafety and principles. Currently there are few studies, such Miyawaki et al. tested the acute toxicities of SNH choosing CNT as one of references^11–14^.

Cell death is the direct reflection of nanotoxicity, and different death mechanisms determine dissimilar toxic effects^15^. So it is vital to clarify the exact death-associated mechanism in nanotoxicity evaluation. Currently, multiple pathways of cell death are confirmed in mammals^16^. Apoptosis, as the most classic one, is a developmental remodeling program and a defensive, organized self-destruction of the cell in reaction to severe damage^17,18^. Distinctively, necrosis triggers the release of intracellular contents, causing greater toxicological response and inflammatory reaction^19^. The necrotic death of cells is usually characterized by swelling of organelles, rupture of plasma membrane, and lysis of cytoplasm^20^. Based on the signaling transductions and the events occurring in particular cellular organelles, the non-apoptosis pathways can be further divided into necroptosis, pyroptosis, ferroptosis, oxytosis, etc^21^. So far, it is not clear how SNH induce cell death and what is the difference between SNH and CNT in this regard.

Cell death is generally initiated by organelle stress^22^. More accurately, nanomaterials usually trigger the death-associated signaling by their interactions with different proteins or membrane in organelles. In effect, distinct proteins, especially receptors, are reported to function in the cell death induced by a variety of nanomaterials^23^. Compared to nanotubes which have been extensively explored, there actually exists a huge vacancy on the study of interactions between receptor and SNH. Besides, membrane, as another important cellular component, significantly impacts the function and stability of receptors in organelles. So the simultaneous investigations on both receptor and membrane will be beneficial for better understanding the mechanism of organelle stress induced by nanomaterials in cell death process. However, this is ordinarily neglected in most of nanotoxicological studies.

In this study, we focus on identifying the differences between SNH and CNT in nanotoxicity and mechanism at cellular and even molecular levels, in order to draw a panorama of the cell death caused by these two types of nanocarbons. Macrophage, as a critical member of initial immune system, is investigated as cell model in the study. Here, SNH are compared with four types of nanotubes, including single-walled carbon nanotubes (SNT) and multiple-walled carbon nanotubes (MNT), throughout the study. For the first time, SNH and CNT are found different in many aspects such as cell uptake, nanocarbon-induced apoptosis, necrosis, pyroptosis, cytokine secretion and protein expression, hydrolase leakage, lysosome stress, membrane disturbance, and interaction with membrane protein. Importantly, it is demonstrated by multiple approaches that SNH possessed better safety and biocompatibility compared to CNT. Based on proteomics and others, a key transmembrane protein, glycoprotein nonmetastatic melanoma protein B (GPNMB) is found to initiate the nanotoxicity. Compared to CNT groups, the weaker nano-GPNMB interaction in SNH group consequently induce lower degree of nano-membrane interplay, lysosome stress, pyroptosis/apoptosis, and finally the hypotoxicity in macrophages, while the involvement of necroptosis, oxytosis/ferroptosis, autophagy, and mitochondrial permeability transition (MPT)-dependent necrosis are basically excluded. It is interestingly to find that the morphology of nanocarbons make the most difference.

## Results

### SNH showed different morphological characteristic with CNT

Transmission electron microscopy (TEM) was firstly utilized to evaluate the morphological feature of SNH and CNT. Especially, four pristine CNT with different nominal lengths (MNT1, MNT2, SNT1, and SNT2) were chosen to compare with SNH throughout the study. As shown in Supplementary Fig. 1, SNH exhibited unique spherical characteristic and cone-like structure (red arrows), significantly differing from the cannular shapes of nanotubes. SNT dispersed in bundle forms which were parallelly constituted by dozens of single nanotube as the structural unit (Supplementary Fig. 2). Though very different in morphology, it was noticed in Supplementary Fig. 1 (red marks) that five nanocarbons possessed similar cross-section diameters (20–40 nm) which was confirmed by the quantitative statistical analysis based on TEM imaging (Supplementary Fig. 3a). Supplementary Fig. 3 also illustrated that SNH had an extremely small aspect ratio (or length/width ratio) compared to nanotubes, indicating the huge differences between these two forms of nanocarbons in geometry. Next, the dispersive characteristics of nanocarbons in aqueous medium were detected by dynamic light scattering (DLS) technology. Interestingly, the dispersive forms of SNH and CNT showed similar size distributions, especially among MNT2, SNT1, and SNH, although they greatly differed in morphology (Supplementary Fig. 4). Additionally, the polydispersity index (PDI) illustrated that SNH and MNT1 had a relatively small size distribution, while other nanomaterials possessed relatively big values (Supplementary Fig. 4c), though all these data are within the range of literature reports^24,25^. These studies hinted that most of nanocarbons did not dispersed in monodispersive pattern, but aggregated in medium to form particles in nano-scale. Generally, SNH demonstrated very different geometric feature from CNT, but the dispersive forms of five nanocarbons in aqueous medium possessed similar size characteristics (Supplementary Fig. 5), thus attenuating the possible size effect on nano-bio interactions^23^.

Besides, five nanocarbons were further characterized by other approaches. Raman spectrometer confirmed their structure compositions (Supplementary Fig. 6). Fourier transform infrared spectroscopy (FTIR) (Supplementary Fig. 7) revealed no significant stretching vibration at the range of 1700–1750 cm^−1^, indicating the absence of carboxyl groups, namely low-level of surface oxidation. Thermal gravity analysis (TGA) demonstrated over 93% weight losses of elemental carbon (Supplementary Fig. 8), suggesting the high purities of nanocarbons. X-ray photoelectron spectroscopy (XPS) (Supplementary Fig. 9 and 10) showed that the overwhelming majority of nanocarbon components were carbon and oxygen, while iron, copper, and zinc were not detected. This was further confirmed by inductively coupled plasma mass spectrometry (ICP-MS) (Supplementary Fig. 11), in which the mass ratios of Zn, Cu, Ni, Co, Fe were all below 1‰, even below 0.1‰. In summary, SNH and CNT used here were basically qualified for subsequent comparison studies, since all of them possessed the similar components, high purities, similar surface functionalizations and low levels of metal catalysts. Thus we assured that the following biological difference between SNH and CNT could basically attribute to their difference in morphological characteristics.

Additionally, it was confirmed in Supplementary Fig. 12 that SNH was quite stable in plasma, based on the facts that there was no aggregation tendency or particle size change for 7 days and no degradation for 3 days in this group. The stability of CNT in plasma varied from 1 to 7 days, still longer than the time of cellular experiment.

### Macrophages internalized less SNH by phagocytosis compared to CNT

**Fig. 1:**
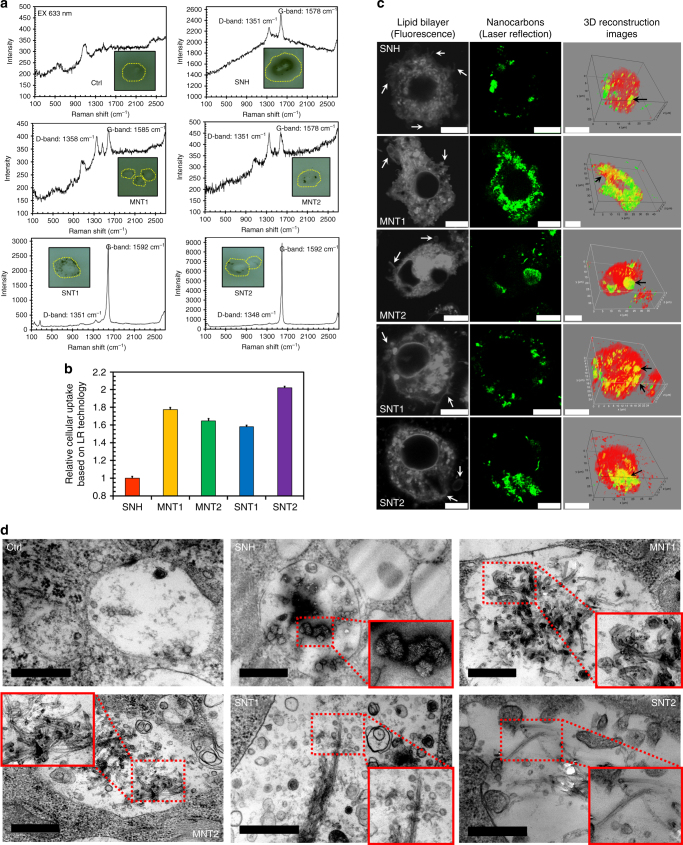
Macrophages internalized less SNH by phagocytosis compared to CNT. **a** Raman spectroscopy of intracellular nanocarbons after cellular incubations. The insert graphs showed the corresponding cells detected by microscope. **b** Relative cellular uptake comparison of five nanocarbons based on laser reflection (LR) signal detection (*n* = 3). **c** Confocal images of cells after nanocarbon incubations. Intracellular nanocarbons were detected by CLSM via LR technology. Cell membrane and endomembrane were stained with lipid dye DiI. White arrows indicated the membrane wrinkles. 3D reconstruction by Imaris software illustrated the co-localizations of nanocarbons with DiI-labeled vesicles (yellow spots, black arrows). Scale bar: 10 μm. **d** Transmission electron microscopy images of intracellular lysosomes after nanocarbon incubations. The magnified insets showed the significant morphological features of nanocarbons. Scale bar: 500 nm. In **b**, three independent nanocarbon incubations were performed in study. Data were expressed as mean ± s.d

Scanning electron microscopy (SEM) observation revealed that all five nanocarbons adsorbed on the surface of J774A.1 cell membrane in aggregative forms (Supplementary Fig. 13, arrows). Confocal laser Raman microscopy (CLRM) clearly demonstrated the internalizations of nanocarbons because of the unique intracellular Raman signals for five nanocarbons at D-band and G-band (Fig. 1a). The cellular uptake was also verified by flow cytometry, as evidenced by the increased side scatter signals by intracellular nanocarbons (Supplementary Fig. 14).

To evaluate the intracellular nanocarbons, a label-free laser reflection (LR) technology based on confocal laser scanning microscopy (CLSM) was utilized here^26^. Distinct from the fluorescently labeled assay, LR technology detected the reflective laser signals of intracellular particles after laser irradiation (Supplementary Fig. 15). As shown in Supplementary Fig. 16, intracellular nanocarbons exhibited prominent LR signals, demonstrating the feasibility of this approach. In addition, the acellular models containing nanocarbons based on polyacrylamide gel were constructed to evaluate the connection between mass concentrations and LR intensities (Supplementary Fig. 17). The good correlation in Supplementary Fig. 18 revealed that LR technology could be used for the quantitative evaluation of nanocarbons. Importantly, it was found that the intracellular LR intensities also correlated well with the nanomaterial concentrations after cellular uptakes of nanocarbons (Supplementary Fig. 19). The linear correlations in acellular and cellular models linked the extracellular and intracellular nanocarbons, making it possible to quantitatively compare the cellular uptake among different nanocarbons. Figure 1b illustrated that the internalized amounts of CNT were 1.6–2.0 times that of SNH when the incubation concentration of nanocarbons was the same, which revealed the significant impact of nanocarbon morphology on their cellular uptakes. Besides, the confocal based study of SNH and CNT demonstrated their significantly co-localization with fluorescent-labeled lipid bilayers (Fig. 1c, black arrows), indicating a vesicle-mediated pathway. TEM further confirmed the lysosome locations of five nanocarbons (Fig. 1d). In addition, both CLSM (Fig. 1c, white arrows) and TEM (Supplementary Fig. 20) investigations showed the SNH- and CNT-induced membrane deformation. Totally, it was concluded SNH and CNT could be internalized into cells via phagocytosis pathway but they had different amount of uptake due to their dissimilarity in geometry. The phagocytosis mechanism was further validated by the reductive internalizations at low temperature (Supplementary Fig. 21), because membrane reformation and fusion in phagocytosis always need energy supply.

Besides, different cancer cells (Caco-2, MDA-MB-231, and Hela) were compared with macrophages in cell uptake pattern (Supplementary Fig. 22, 23), given the potential tumor-directed applications of SNH. First, the different uptake levels of nanocarbons were found in different cell lines. Macrophages exhibited the highest endocytosis for most of nanomaterials in all cell lines. Then, three canonical endocytosis pathways were identified by using pharmaceutical inhibitors. For each nanocarbon group, there was obvious difference in the ratios of these three pathways in different types of cells. Interestingly, SNH exhibited a distinct endocytosis mechanism from that of MNT2 and SNT1 in four cell lines, although these three nanocarbons shared the same size range, suggesting the influence of morphological structure on the endocytosis pathway of nanocarbons.

### SNH caused lower nanotoxicity than CNT

**Fig. 2:**
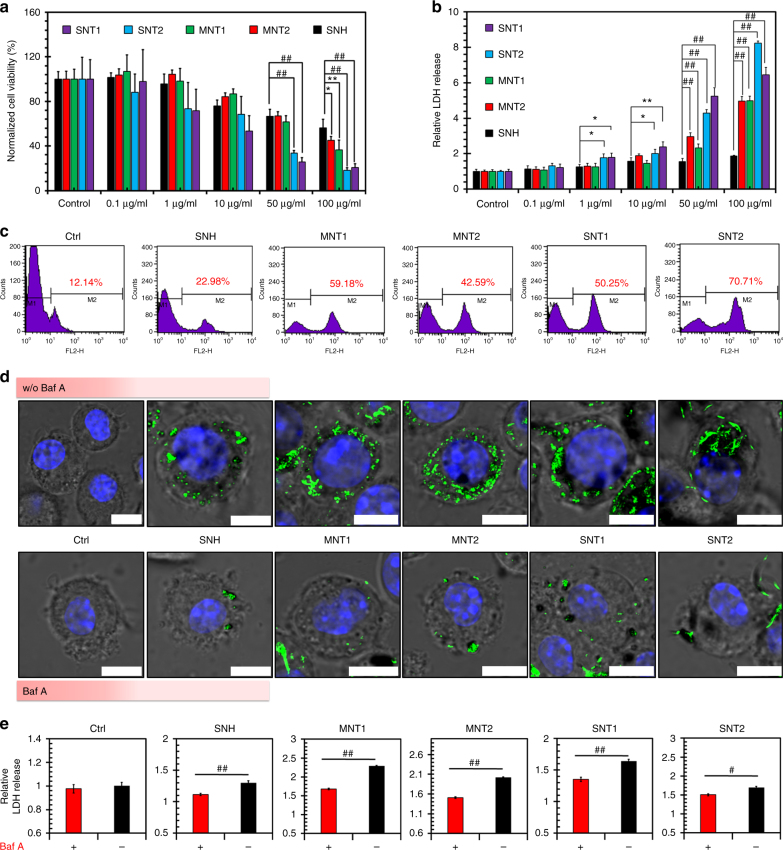
SNH caused lower nanotoxicity than CNT. **a**, **b** Cellular viability comparisons of different nanocarbons detected by **a** MTT assay and **b** LDH release investigations (*n* = 5). **c** Flow cytometry analysis of nanocarbon-incubated cells via PI labeling assay. **d** Confocal images of cells after nanocarbon incubations with and without Baf A addition. Intracellular nanocarbons were detected by laser reflection (LR) technology and shown with pseudo green color. Scale bar: 10 μm. **e** Cytotoxic analysis of different nanocarbons based on LDH release assay with and without Baf A addition *(n* = 4). In **a**, **b**, and **e**, data were presented as means ± s.d. Statistical significances were calculated by Student’s *t*-test: *p < 0.05, **p < 0.01, ^#^*p* < 0.005, ^##^*p* < 0.001

The nanotoxicity of different nanocarbons to cells was firstly investigated via 3-(4,5-dimethylthiazol2yl)-2,5-diphenyltetrazolium bromide (MTT) assay. It was shown in Fig. 2a that SNH caused lower cytotoxicity than four types of CNT. This was further confirmed by lactate dehydrogenase (LDH) release assay, in which SNH triggered the leakage of LDH significantly less than CNT groups (Fig. 2b). The hypotoxicity of SNH was also demonstrated by flow cytometry based on propidium iodide (PI) labeling assay (Fig. 2c). The incubation of five nanocarbons increased the numbers of dead cells labeled by PI in comparison with negative control, but SNH-induced minimal cell death among the five nanocarbons (22.98% for SNH vs >40% for other CNT). Next, single-cell gel electrophoresis (comet assay) was used to investigate the possible nanocarbon-triggered DNA disruption. Compared to control group, SNH treatment did not change the fluorescent distribution of labeled DNA (Supplementary Fig. 24), suggesting no obvious impact of SNH to DNA integrity. However, four CNT groups clearly exhibited comet-like characteristics (Supplementary Fig. 24, arrows), which indicated higher genotoxicities than SNH. Based all above findings, it was clear that SNH caused obviously lower nanotoxicity than CNT.

To explore the initiation mechanism of toxicity, the correlation of toxicity with cellular uptake of nanocarbons was investigated by pharmacological inhibition strategy. Two common inhibitors^27^, bafilomycin A (Baf A, a blocker of H^+^ ATPase) and cytochalasin D (Cyto D, a microfilament depolymerization inducer) were utilized here. By means of LR imaging in CLSM, Baf A was found to obviously inhibit the internalizations of five nanocarbons (Fig. 2d). Cyto D showed the same effect on cellular uptakes of SNH and CNT (Supplementary Fig. 25a). Interestingly, the cytotoxicities of nanocarbons were significantly alleviated by the additions of both Baf A and Cyto D (Fig. 2e, Supplementary Fig. 25b). These findings indicated that the phagocytosis of nanocarbons was main prerequisite of their nanotoxicities. In other words, the toxic effects of nanocarbons might initiate from intracellular but not extracellular environment.

However, the above conclusion raises a question that the hypotoxicity of SNH might be attributed to their lower intracellular level owing to less cell uptake (Fig. 1b), but not the structure feature. To address this issue, the incubation concentrations of nanocarbons were adjusted to assure the same intracellular contents among the five types of nanocarbons (Supplementary Note 1, Supplementary Table 1). Based on the normalization of cell uptake level, it was demonstrated in apoptosis–necrosis assay (Supplementary Fig. 26a and b) that SNH still showed lower cytotoxicity than CNT. Additionally, both MTT and LDH assays had the same findings (Supplementary Fig. 26c and d), even if the macrophage uptake of SNH was identical with four types of CNT. So it was concluded that the low cytotoxicity of SNH was closely associated with its morphological structure.

### SNH-induced less apoptosis and necrosis than CNT

**Fig. 3:**
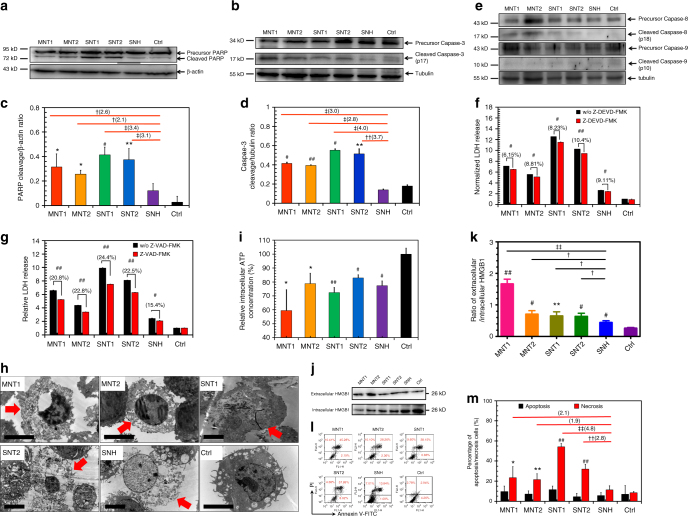
SNH-induced less apoptosis and necrosis than CNT. **a**, **b** Western blot analyses of **a** PARP and **b** caspase-3 cleavages after different nanocarbon incubations. **c**, **d** Quantitative cleavage ratio measurements of **c** PARP and **d** caspase-3 in nanocarbon-incubated cells according to the integrated optic density (IOD) value detection based on western blot imaging (*n* = 3). **e** Western blot analyses of caspase-8 and caspase-9 cleavages after nanocarbon incubations. **f**, **g** Cytotoxicity detections of different nanocarbons with and without two caspase inhibitors, **f** Z-DEVD-FMK and **g** Z-VAD-FMK) (*n* = 4). **h** Transmission electron microscopy images of dead cells caused by different nanocarbons. Red arrows showed the typical necrosis characteristics of cells. Scale bar: 5 μm. **i** Intracellular ATP detection after nanocarbon incubations (*n* = 4). **j** Immunoblot analysis of extracellular and intracellular HMGB1 after cellular incubations with different nanocarbons. **k** Quantitative ratio of extracellular HMGB1 to intracellular HMGB1accorind to the IOD detection based on WB imaging (*n* = 3). **l** Flow cytometry analysis of cells based on Annexin V/PI assay after nanocarbon incubations. **m** Quantitative comparison of apoptosis and necrosis caused by different nanocarbons detected by apoptosis/necrosis assay kit (*n* = 4). In **c**, **d**, **f**, **g**, **i**, **k** and **m**, data were presented as means ± s.d. Statistical significances were calculated by Student’s *t*-test. In **c**, **d**, **k**, and **m**, data were compared with control (Ctrl) and SNH groups separately. Versus Ctrl: *p < 0.05, **p < 0.01, ^#^*p* < 0.005, ^##^*p* < 0.001. Versus SNH: ^†^*p* < 0.05, ^††^*p* < 0.01, ^‡^*p* < 0.005, ^‡‡^*p* < 0.001. The values in brackets denoted the data ratios compared to SNH group. In **f**, **g**, data were compared with no inhibitor adding groups for each type of nanocarbons: ^#^*p* < 0.005; ^##^*p* < 0.001

As one of potential mechanisms of nanocarbon-induced cytotoxicity, apoptosis, the classical form of programmed cell death (PCD), was firstly investigated. Poly-ADP-ribose polymerase (PARP) cleavage and caspase-3 (CASP-3) activation, two hallmarks of apoptosis, were analyzed by western blot (WB). As illustrated in Fig. 3a, b, CNT triggered obvious cleavages of PARP and CASP-3 compared to control and SNH group, and in more detail, CNT triggered 2.1–3.4 folds of PARP and 2.8–4.0 folds of CASP-3 compared to SNH group (Fig. 3c, d). The significant increases of cleavage ratios in CNT groups confirmed the apoptosis mechanism. Caspase-8 (CASP-8) and caspase-9 (CASP-9), as the upstream regulative proteins, were further analyzed (Fig. 3e). It was shown that CNT caused prominent cleavages of CASP-8 but with little effect on CASP-9. The co-activations of CASP-3 and CASP-8 revealed that four types of CNT-induced apoptosis through extrinsic but not intrinsic pathway^16^. Distinctively, SNH showed almost no effect on the activations of PARP, CASP-3, and CASP-8 in all tests above, and its cleavage ratios were significantly less than CNT groups (Fig. 3c, d), indicating the extremely low apoptosis inducibility of SNH. Next, Z-VAD-FMK (pan-caspase inhibitor) and Z-DEVD-FMK (specific caspase-3 inhibitor) were utilized to explore the proportion of apoptosis in PCD. As the result, Z-VAD-FMK decreased the cytotoxicities of five nanocarbons more significantly than Z-DEVD-FMK (Fig. 3f, g). Therefore, it was supposed that there might be other pathways played major role in the PCD induced by nanocarbons, except CASP-3 regulated apoptosis. Additionally, even in the case of identical intracellular content for five types of nanocarbons, both inhibitors also significantly reduced the cell death (Supplementary Fig. 27). So, it was indicated that the cell death mechanism was not associated with the uptake level of nanocarbons.

Necrosis was another type of cell death process besides apoptosis. For many years it was considered to be unregulated and non-programmed. However, it was recently reported that necrosis could undergo programmed process through a series of intracellular signal transductions^28^. To determine whether SNH- and CNT-induced cell death through necrosis, TEM was firstly used to observe the cell morphologies. As shown in Fig. 3h, dead cells after incubation with nanocarbons exhibited obvious necrosis characteristics, in which cell membrane was destroyed and cytoplasm was vacuolated. ATP depletion as the hallmark of necrosis process was also detected here. Figure 3i illustrated that intracellular ATP levels decreased significantly after the addition of SNH and CNT. Besides, high-mobility group box 1 protein (HMGB1) is considered as the specific marker to distinguish necrosis and apoptosis. In health and apoptosis cells, HMGB1 is retained in nucleus, but in necrosis cells HMGB1 can transport from nucleus to cytosol and then to extracellular space^29^. Here we utilized WB and CLSM to determine the location of HMGB1 in cells. The assay of extracellular and intracellular HMGB1 by immune blot indicated that nanocarbons caused the leakage of HMGB1 (Fig. 3j). MNT induced the largest leaking and SNH the least. The corresponding quantification data in Fig. 3k verified the same finding. Likewise, the fluorescence intensities of HMGB1 in cell nuclei apparently decreased after the incubation of SNH and CNT (Supplementary Fig. 28). In summary, these findings revealed that SNH and CNT triggered cell death through necrosis pathway in addition to apoptosis.

To further identify which pathway was dominant during the PCD process, Annexin V/PI assay based on flow cytometry was performed. Compared to the minor change of early apoptosis cells (Annexin V single positive labeling), five nanocarbons induced significant increase of PI single positive labeling cells (Fig. 3l), which was the main indication of necrosis cells. Besides, the quantitative comparison based on apoptosis/necrosis assay kit also revealed that SNH and CNT caused more significant necrosis than apoptosis (Fig. 3m, Supplementary Fig. 29). Importantly, the comparison among nanocarbons as shown in Fig. 3m indicated that CNT-induced 1.9–4.8 folds of necrosis compared to SNH, indicating the much lower necrosis inducibility for SNH. So it was concluded that necrosis dominated in PCD induced by nanocarbons, and notably, SNH always triggered less cell death compared to CNT, no matter through necrosis or apoptosis.

### SNH generated less impact on protein expression than CNT

**Fig. 4:**
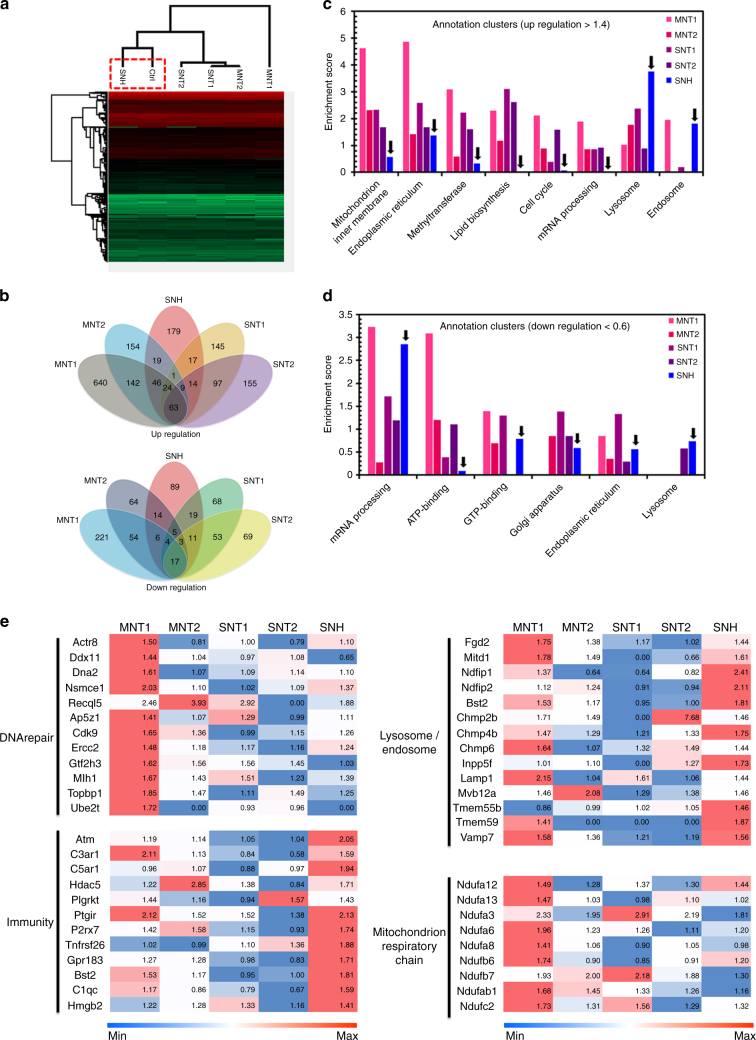
SNH generated less impact on protein expression than CNT. **a** Hierarchical clustering analysis on the changes of cellular proteins investigated by LFQ proteomics after incubation of nanocarbons. **b** The Venn diagrams of cellular proteins with more than 40% expression change over control group after treatment of nanocarbons. **c**, **d** The gene functional annotation clustering of the **c** upregulated and **d** downregulated proteins based on enrichment analysis after the incubation of different nanocarbons. **e** The heat maps on the expression comparison of changed proteins that involved in part of key cellular processes after the incubation of nanocarbons. The values in heat maps represented the expression ratios of identified proteins compared to control group

The downstream cellular response to different nanocarbons was further detected by label-free quantification (LFQ) proteomic technology based on mass spectrometry (MS). Over 5000 proteins were identified after the incubation with nanocarbons (Supplementary Data 1). First, the hierarchical clustering analysis showed that the protein expression characteristics in SNH group were more close to those in blank control compared to CNT (Fig. 4a). Then, SNH group was found to be different from CNT in category and quantity for cellular proteins which had an expression change of more than 40% over control (Fig. 4b). Functional cluster analysis (DAVID Bioinformatics Resources 6.8) indicated that the changed proteins by SNH incubation were less involved in the key cellular processes including gene repair, protein biosynthesis, and metabolism, but more in vesicle transport and lysosomes, while CNT mainly affected mitochondria and ER associated proteins (Fig. 4c, d). The heat map of protein expressions confirmed the lower effect of SNH to DNA and mitochondria (Fig. 4e). In summary, the damages on mitochidria and ER might be the downstream executors for nanocarbon-induced cell deaths. More importantly, compared to CNT, SNH showed less impact on these proteins involving key processes.

Additionally, to confirm that the cellular response to nanocarbons were attributed to their morphological structure but not the uptake amount, the LFQ proteomic analysis was also performed based on the identical intracellular content for five types of nanocarbons. Interestingly, almost the same facts as above were observed (Supplementary Data 2). As illustrated in Supplementary Fig. 30a, the hierarchical clustering analysis manifested closer similarity of SNH with control group compared to CNT. Likewise, both gene enrichment analysis (Supplementary Fig. 30c and d) and protein expression comparison (Supplementary Fig. 30e) revealed that SNH-induced the upregulation of transport associated proteins, but had less effect to DNA and mitochondria associated proteins compared to CNT. These studies demonstrated that the lower cellular response to SNH was closely related to its structure.

### SNH triggered less pyroptosis than CNT

**Fig. 5:**
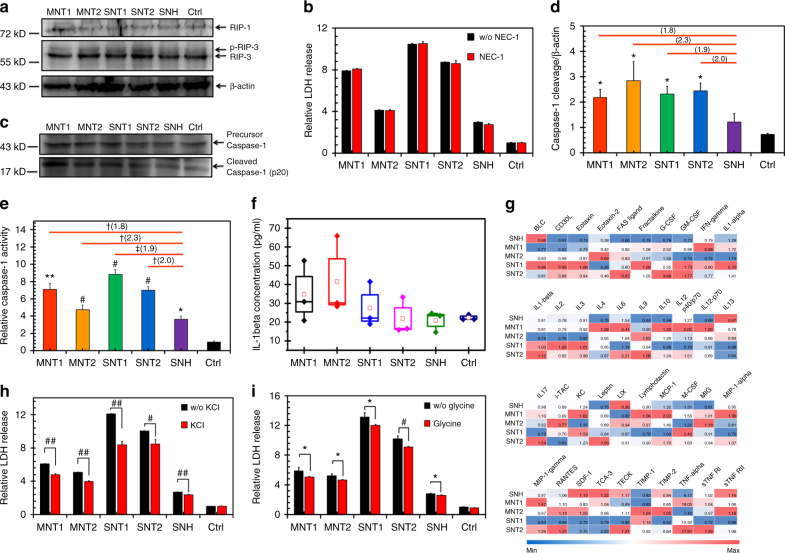
SNH triggered less pyroptosis than CNT. **a** Western blot analyses of RIP1 and RIP3 kinases after nanocarbon incubations. **b** Cytotoxicity detection of different nanocarbons with and without RIP-specific inhibitor NEC-1 addition (*n* = 4). **c** Western blot analysis of caspase-1 cleavage induced by different nanocarbons. **d** Quantitative cleavage ratio measurement of caspase-1 in nanocarbon-incubated cells based on the integrated optic density (IOD) value detection during western blot imaging process (*n* = 3). **e** Caspase-1 activity determination after cellular incubations of different nanocarbons based on specific assay kit (*n* = 3). **f** Extracellular IL-1β measurement induced by different nanocarbons detected by ELISA. Each data point represented an independent detection, and the box denoted the standard deviation for each group (*n* = 3). **g** The heat map of extracellular inflammatory cytokines induced by nanocarbons based on the protein array technology. The data in map represented the secretion changes of cytokines caused by nanocarbons compared to control group. **h**, **i** Cytotoxicity detections of different nanocarbons with and without two pan-inhibitors of pyroptosis, **g** KCl and **h** glycine additions (*n* = 4). From **b** to **h**, data were all presented as means ± s.d. and the statistical significances were calculated by Student’s *t*-test. In **d** and **e**, data were compared with control (Ctrl) and SNH groups respectively. Versus Ctrl: *p < 0.05, **p < 0.01, ^#^*p* < 0.005, ^##^*p* < 0.001. Versus SNH: ^†^*p* < 0.05, ^††^*p* < 0.01, ^‡^*p* < 0.005. The values in brackets denoted the data ratios compared to SNH group. In **g** and **h**, data were compared with no inhibitor adding groups for each type of nanocarbons: *p < 0.05; ^#^*p* < 0.005; ^##^*p* < 0.001

Regulated necrosis was not a single-cell death mechanism, which was composed of different pathways including necroptosis, pyroptosis, oxytosis, ferroptosis, and MPT-dependent necrosis (some of them will be tested late)^20^. Since necroptosis is the best-characterized form of regulated necrosis, it was first explored here via the expression detection of RIP1 and RIP3 which regulate the necroptosis-mediated necrosis as kinases^30^. As shown in Fig. 5a, five nanocarbons did not alter the expression of RIP1 or induce more phosphorylation of RIP3 compared to negative control. Besides, Necrostatin-1 (NEC-1), as the specific inhibitor of RIP kinases^31^, did not reduce cytotoxicities caused by SNH and CNT (Fig. 5b). Finally, on the basis of normalization of uptake level of nanocabons, NEC-1 still had no effect on the cell viabilities (Supplementary Fig. 31). These results indicated that the necrosis processes induced by SNH and CNT were not mediated by necroptosis pathway.

**Fig. 6:**
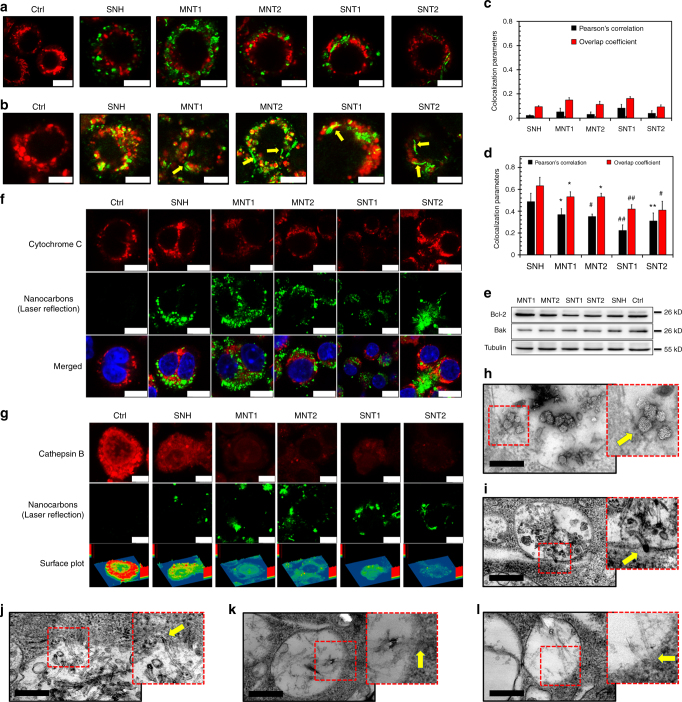
SNH initiated less lysosome stress than CNT. **a** Co-localization images of intracellular nanocarbons (pseudo green color, laser reflection detection) with mitochondria (red color, labeled with MitoTracker) detected by CLSM. Scale bar: 7.5 μm. **b** Co-localization images of intracellular nanocarbons (pseudo green color, laser reflection detection) with lysosomes (red color, labeled with LysoTracker) viewed by CLSM. Yellow arrows indicated the penetrations of CNT through lysosome membrane. Scale bar: 7.5 μm. **c**, **d** Co-localization parameter measurements of different nanocarbons with **c** mitochondria and **d** lysosomes respectively based on CLSM investigations (*n* = 5). **e** Western blot analyses of Bcl-2 and Bak after nanocarbon incubations. **f**, **g** Intracellular distribution images of two organelle-specific proteins, **f** Cytochrome C (mitochondria location) and **g** Cathepsin B (lysosome location) after the cellular incubations of nanoarbons. Scale bar in **f** and **g**: 10 μm. **h**–**l** Transmission electron microscopy images of the interactions of different nanocarbons with lysosome membrane. The insert graphs indicated the moderate contact of **h** SNH with membrane, and the striking membrane penetrations for **i** MNT1, **j** MNT2, **k** SNT1, and **l** SNT2. Scale bar in **h**–**l**: 500 nm. In **c** and **d**, data were presented as means ± s.d. Statistical significances were calculated by Student’s *t*-test. Data were all compared with control (Ctrl) group: *p < 0.05, **p < 0.01, ^#^*p* < 0.005, ^##^*p* < 0.001

Pyroptosis, as another common regulated necrosis pathway, was distinct from other pathways in mechanism. Caspase-1 (CASP-1) dependency was the specific characteristic of pyroptosis^32^. In pyroptosis process, caspase-1 activation facilitates the secretion of activated inflammatory cytokines including IL-1β and the release of intracellular proinflammatory factors^33^. Here, CNT were found to trigger significant cleavage of CASP-1 (Fig. 5c, d) while SNH-induced only slight increase of cleaved CASP-1 expression compared to control. Likewise, the caspase-1 activity assay in Fig. 5e illustrated the activations of CASP-1 after incubations of SNH and CNT. Also, the activity enhancements caused by CNT were significantly higher than SNH group. Next, enzyme-linked immunosorbent assay (ELISA) test showed that CNT enhanced the level of extracellular IL-1β, while SNH had little effect (Fig. 5f). Additionally, 40 types of inflammatory cytokines in medium were also detected here based on the protein array technology. The heat map in Fig. 6g exhibited that SNH caused the least changes of cytokine level in most cases, suggesting the lower ability of inducing cytokine secretion, which was also the hallmark of pyroptosis. So these findings revealed the lower inducibility of SNH than CNT in terms of inflammatory response and pyroptosis.

Finally, pyroptosis also facilitates the formation of membrane pores and dissipates cellular ion gradients, while ion gradient inhibitor KCl and cytoprotective agent glycine can non-specifically suppress pyroptosis by blocking the ion flux in cell membrane^34^. Here, KCl significantly reduced the cytotoxicities of SNH and CNT (Fig. 5h), and so did glycine (Fig. 5i). Even in the case of identical intracellular level among all type of nanocarbons, KCl and glycine also significantly reduced the cell death (Supplementary Fig. 32). In short, we demonstrated here that SNH- and CNT-induced cell death through pryroptosis-mediated necrosis, and more importantly, SNH triggered obviously less pryroptosis compared to CNT.

### SNH initiated less lysosome stress than CNT

Both mitochondria and lysosomes played important roles during the initiation of cell death. To investigate the correlation of nanocarbon-caused toxicities with them, co-localization analysis based on CLSM was first performed here. It was illustrated in Fig. 6a that intracellular SNH and CNT did not co-localize with mitochondria. This was further verified by the very low co-localization parameters (Pearson’s correlation and overlap coefficient) as shown in Fig. 6c. Next, WB analysis in Fig. 6e indicated that SNH and CNT did not affect the levels of Bcl-2 and Bak, both of which are important regulative proteins in mitochondria-dependent cell death^35^. Additionally, CLSM imaging showed that five nanocarbons did not alter the distribution and intensity of cytochrome C which specifically locates in mitochondria (Fig. 6f). These findings revealed that SNH and CNT had little effect on the function and integrity of mitochondria. Combining with the fact that nanocarbons did not activate caspase-9 (Fig. 3e), it was concluded that internalized SNH and CNT did not directly interact with mitochondria and trigger cell death through mitochondria-dependent pathways, including MPT-dependent necrosis.

Distinctively, CLSM observation exhibited that most intracellular nanocarbons located in lysosomes (Fig. 6b), and SNH exhibited even significantly higher co-localization parameters than CNT (Fig. 6d). Unlike CNT, the internalized SNH did not notably penetrate and disperse into cytoplasm but mostly stayed within lysosomes (Fig. 6c, yellow arrows). It was also found in Fig. 6g that both SNH and CNT significantly reduced the fluorescence intensities of cathepsin B (CTSB) in lysosomes, indicating the lysosome disturbance caused by nanocarbons and the resultant lysosome membrane penetration (LMP), which was the landmark of lysosome stress^36^. Furthermore, it was demonstrated by FITC-dextran (FD) imaging that five nanocarbons, especially CNT-induced obvious spillage of FD from lysosomes to cytoplasma (Supplementary Fig. 33). Both the surface plot in Fig. 6g and FD imaging manifested that SNH caused less leakage of CTSB and FD than CNT, revealing the lower SNH-induced lysosome stress. More remarkably, TEM observation clearly confirmed that all CNT interacted with lysosome membrane in a directly penetrating pattern, but SNH acted much moderately (Fig. 6h-l). In general, SNH triggered weaker membrane disturbance and LMP compared to CNT, thus keeping more degrading enzymes detained within lysosomes and finally leading to hypotoxicity.

Reactive oxygen species (ROS) is usually considered as the main mechanism for oxytosis/ferroptosis^37^. In this study, ROS-specific inhibitor NAC had little impact on the cytotoxicities of SNH and CNT (Supplementary Fig. 34). Together with the extremely low contents of metal catalysts (Fe, Ni, Cu) that always act as initiator of oxytosis/ferroptosis, it was indicated that the observed cell death was basically not mediated by ROS or ROS-dependent oxytosis/ferroptosis.

The possibility of autophagy-regulated cell death was also explored here. CLSM investigation revealed that intracellular nanocarbons could neither induce more expressions of LC-3B protein, nor co-localize with the labeled autophagosomes (Supplementary Fig. 35)^38^, manifesting that SNH and CNT did not trigger the autophagy-regulated cell death.

### SNH brought lower membrane disturbance than CNT

**Fig. 7:**
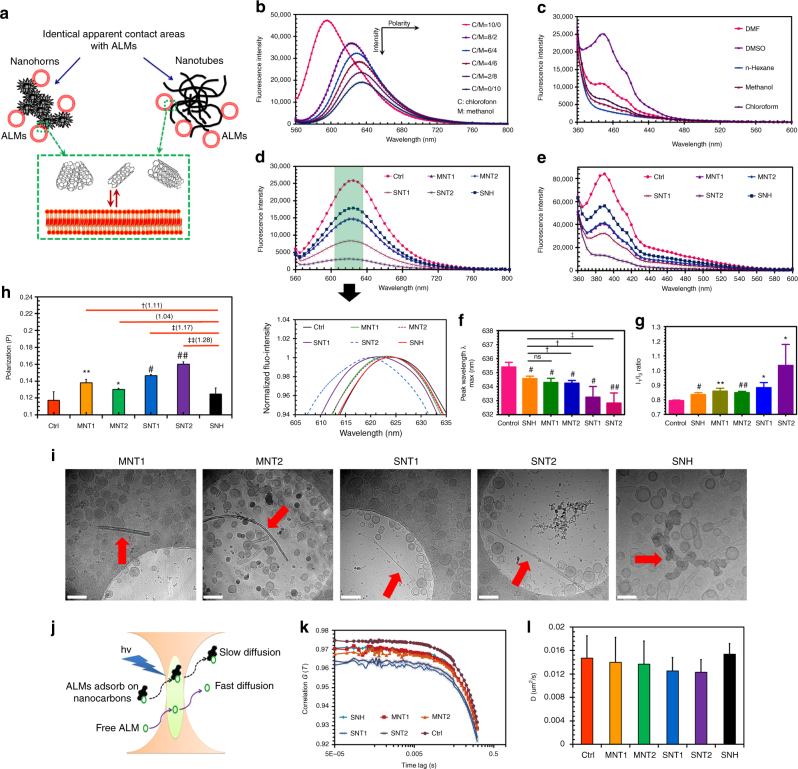
SNH brought lower membrane disturbance than CNT. **a** Schematic of membrane interactions induced by different nanocarbons. Artificial lipid membranes (ALMs) were fabricated in the investigations. Since nanocarbons usually assembled in aqueous medium, the mass concentrations of five nanocarbons were modulated to keep the identical apparent contact area, which was calculated based on hydrodynamic radius (R_H_). **b**, **c** Fluorescence spectrums of two environment sensitive probes, **b** nile red and **c** pyrene in solvents with different polarities. **d**, **e** Fluorescence spectrums of ALMs labeled with **d** nile red and **e** pyrene respectively after different nanocarbon incubations. The insets represented the normalized spectrums of fluorescent ALMs near emission peaks. **f** The comparison of *λ*_max_ after the incubation of nanocarbons with ALMs. Three independent spectrum scanning were performed in each experiment group. **g** The quantitative detection of *I*_1_/*I*_3_ after nanocarbon incubation based on the spectrum scanning of pyrene-labeled ALMs (*n* = 3). **h** Polarization (*P*) measurements of ALMs labeled with DPH after the incubations of different nanocarbons (*n* = 4). **i** Cryo-transmission electron microscopy images of ALMs after different nanocarbon incubations. Red arrows showed the nanocarbons in aqueous medium. Scale bar: 100 nm. **j** Schematic of fluorescence correlation spectroscopy (FCS). Fluorescent ALMs that bound with nanocarbons could be distinguished from free ALMs by FCS because of the reduced diffusion rates. **k** Autocorrelation curves of ALMs after nanocarbon incubations detected by FCS. **l** Diffusion coefficient detection of ALMs after different nanocarbon incubations based on autocorrelation curve fitting (*n* = 12). In **f**, **g**, **h**, and **l**, data were presented as means ± s.d. Statistical significances were calculated by Student’s *t*-test. Data were compared with control (Ctrl) and SNH groups respectively. Versus Ctrl: *p < 0.05, **p < 0.01, ^#^*p* < 0.005, ^##^*p* < 0.001. Versus SNH: ^†^*p* < 0.05, ^††^*p* < 0.01, ^‡^*p* < 0.005, ^‡‡^*p* < 0.001. The values in brackets denoted the data ratios compared to SNH group

Although the obvious interactions between lysosome membrane and nanocarbons have been affirmed in Fig. 6h-l, the detailed mechanism was kept unknown. Here, artificial lipid membranes (ALMs) were constructed to investigate the membrane disturbances of nanocarbons by lipid phase analysis based on fluorescence spectrum scanning strategy (Fig. 7a). Nile red and pryene^39,40^, two of environment sensitive fluorescence dyes as verified in Fig. 7b, c, were used to label ALMs in order to monitor the configuration change of phospholipids. It was also approved that nanocarbons did not interfere with the fluorescence spectrum of ALMs (Supplementary Fig. 36). Based on the identical apparent contact areas with ALMs according to DLS diameter conversion via cumulants fitting (Fig. 7a, Supplementary Fig. 37, Supplementary Table 2), all five nanocarbons significantly reduced the spectrum fluorescence intensities of labeled ALMs (Fig. 7d, e), namely, both SNH and CNT led to disturbed configuration of phospholipids. However, SNH exhibited less impact on ALMs compared to CNT. Besides, the normalized spectrum (Fig. 7d) and the quantitative detection on the maximum wavelength (*λ*_max_) revealed the obvious blue-shift of spectrums compared to control (Fig. 7f). It was demonstrated the membrane interaction^41^. More importantly, the significant difference among different test groups could be seen, and SNH caused lower degree of alterations, no matter in fluorescence intensity and blue-shift, thus indicating the lower membrane perturbation from SNH. Because of the environmental sensitivity, pyrene has been effectively used for evaluating the polarity of microenvironments^42^. Reportedly, the ratio of the first (375 nm) and third (385 nm) vibronic peak intensities (*I*_1_/*I*_3_) in pyrene spectrum provided a measure of the apparent polarity of the environment^43^. So in this study we performed quantitative *I*_1_/*I*_3_ detection in addition to the spectrum intensity comparison. As shown in Fig. 7g, five nanocatbons caused the increase of *I*_1_/*I*_3_, suggesting the membrane interaction. The alteration of ratio in SNH group was lower than CNT, also revealing the lower degree of interaction between SNH and ALMs.

The spectrums based on non-negatively constrained least squares (NNLS) fitting algorithm conversion could draw the same conclusion (Supplementary Fig. 38). Additionally, during the detection on membrane fluidity by using a DPH polarization assay^44^, CNT obviously enhanced the fluidity of ALMs (Fig. 7h, Supplementary Fig. 39), while SNH had almost no such effect. This study actually explained why CNT fiercely inserted membrane but SNH had moderate effect as shown in Fig. 6.

The cross interaction of nanocarbons and biomembrane was further investigated by Cryo-TEM. Surprisingly, only a small number of ALMs directly adhered to the surface of nanocarbons (Fig. 7i), indicating that the direct binding between ALMs and nanocarbons might be weak. This was further verified by fluorescence correlation spectroscopy (FCS) via detecting the diffusion coefficients of fluorescent ALMs (Fig. 7j, Supplementary Fig. 40a)^45^. As shown in Fig. 7k, l, after the incubation with five nanocarbons, ALMs exhibited similar autocorrelation curves and the corresponding diffusion coefficients showed no difference with negative control. Also, confocal images confirmed the absence of big fluorescent spots of ALMs on nanocarbons (Supplementary Fig. 40b). It revealed that ALMs might hardly bind to nanocarbon surface. In other words, the affinities of nanocarbons with lipid membrane might not be powerful enough to maintain the continuous binding. This finding was interesting, as we discovered in Fig. 6 that the intracellular nanocarbons intensively interacted with lysosome membrane and some even directly penetrated across the membrane. So, we hypothesized that certain proteins in lysosome might play bridging role during the interactions. These proteins might directly interact with intracellular nanocarbons, facilitating their access to lysosome membrane and initiating the membrane damage.

### Difference in nano-protein interaction between SNH and CNT

**Fig. 8:**
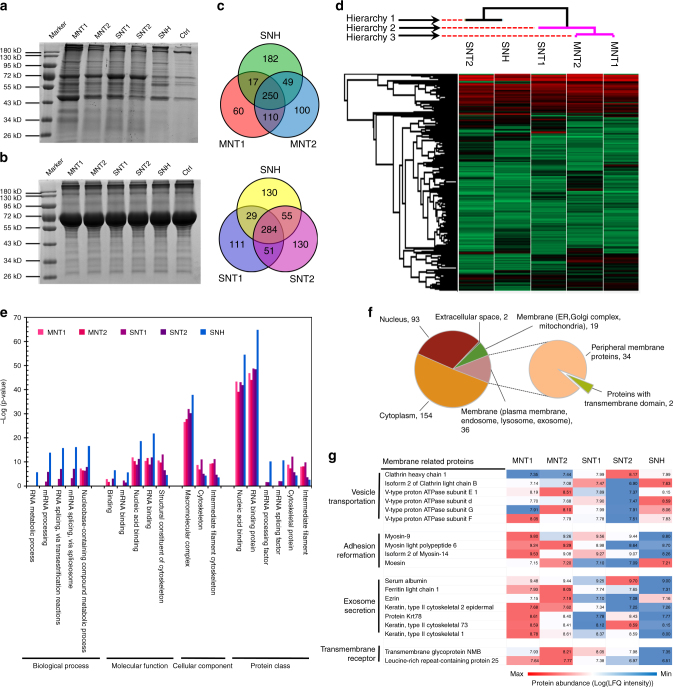
Difference in nano-protein interaction between SNH and CNT. **a** Gel electropherogram of cellular proteins that bound with different nanocarbons after cell lysate incubations. The resultant stripes revealed the high affinities of part intracellular proteins with nanocarbons. **b** Gel electropherogram of different nanocarbon dispersions that contained 0.5% bovine serum albumin (BSA). **c** Venn diagrams of identified proteins among different nanocarbon groups. SNH shared near half of proteins with MNT and SNT separately. **d** Hierarchical clustering analysis of affinitive proteins among five nanocarbon groups, revealing the distinctions of SNH with CNT in protein class. **e** Overrepresentation (OR) tests of affinitive proteins in different nanocarbon groups based on gene ontology (GO) analysis. **f** Intracellular distribution of shared affinitive proteins for five nanocarbon groups. **g** Protein-binding abundance comparisons among different nanocarbon groups via heat mapping analysis. The values in heat map indicated the logarithmic intensities of proteins based on label-free quantification (LFQ) detected by LC-MS/MS

To validate the potential protein-mediated membrane disturbance, we firstly investigated the general interaction of nanocarbons with cellular proteins. A centrifugation-elution strategy similar with immunoprecipitation (IP) was utilized to identify the possible high-affinitive proteins (Supplementary Fig. 41). Fig. 8a illustrated the gel electrophoresis result of surface binding proteins, while Fig. 8b showed the corresponding control before cell lysate incubation. Remarkably, more protein stripes were detected after the incubation of nanocarbons with cell lysate. It revealed that certain cellular proteins replaced bovine serum albumin (BSA) via greater affinities to bind with nanocarbons. Additionally, the difference in molecular weight distributions of adhesive proteins among five nanocarbon groups was noticed (Supplementary Fig. 42). Especially, some stripes of bonded proteins in SNH group were different from CNT groups in distribution and intensity (Supplementary Fig. 42, black arrows), indicating the different nano-protein interaction between these two groups.

The cross activity between cellular proteins and nanocarbons was further verified by mass spectrometry (MS) based on LFQ proteomics technology^46^. Two biological duplicative experiments showed acceptable correlations (Supplementary Fig. 43). Over 400 cellular proteins were discovered to interact with the five nanocarbons (Supplementary Data 3-5). Notably, SNH shared near half of affinitive proteins with MNT and SNT separately (Fig. 8c), suggesting the differences and similarities coexisted in the nano-protein interactions between SNH and CNT. Here, hierarchical clustering analysis of whole affinitive proteins was performed. As shown in Fig. 8d, SNH group exhibited obvious difference from CNT in lower hierarchy (hierarchy 1), revealing their significant dissimilarity in both category and abundance of proteins. Interestingly, the surface bonded proteins on SNH was obviously different from those on MNT2 and SNT1 groups due to the hierarchy discrepancy, although these nanocarbons possessed close size as dispersive forms in medium, thus revealing the significant influence of nanocarbon morphology on nano-protein interaction.

Besides, GO analysis was performed to compare the functional classifications of identified proteins for different nanocarbons. In three common aspects, namely the molecular function (MF), biological process (BP), and cellular component (CC), the proportional distributions of bonded proteins on five nanocarbons exhibited some similarities (Supplementary Fig. 44). However, the overrepresentation (OR) test based on GO analysis clearly revealed the statistical differences in function emphases of the affinitive proteins between SNH and CNT (Supplementary Fig. 45-49). Figure 8e showed such discrepancies between these two nanocarbon groups. Interestingly, SNH exhibited obviously greater emphases in RNA binding and processing including splicing and metabolism, etc (Fig. 8e, red arrows). While in cytoskeletal and structural protein category, SNH showed the lowest representative capacities compared to CNT. However, the differences based on OR test were not so obvious among the four types of CNT groups themselves. So it was indicated that the unique morphology of SNH might be the key issue to trigger different nano-protein cross activity.

Membrane-related proteins, especially transmembrane receptors, are the key mediators during the real cellular uptake process of nanocarbons. So the related screening and identification is of great significance for the exploration on nano-bio interaction. By using trypsin to partly degrade the extracellular sequences of membrane-related proteins, it was found that the cellular uptakes of SNH and CNT decreased (Supplementary Fig. 50 and 51), revealing the involvement and regulation role of certain membrane proteins. Furthermore, the identified affinitive proteins based on MS technology were screened according to the cellular location information derived from Unitprot database. It was shown in Fig. 8f that most proteins located in cytoplasm and nucleus, and only <20% existed in plasmalemma or vesicle membrane. Part of these membrane-related proteins were further analyzed based on the binding abundance differences as in the heat map (Fig. 8g). Interestingly, compared to CNT, SNH group exhibited relatively higher protein abundances in vesicle transportation class, but lower abundances for adhesion and secretion proteins. Two identified transmembrane receptors also displayed an affinity with SNH lower than CNT (see below). In short, SNH and CNT had an obvious interaction with membrane-related proteins, however, they were different in terms of category and abundances of affinitive proteins, likely due to the morphology effect.

### SNH triggered less GPNMB-regulated cell death

**Fig. 9:**
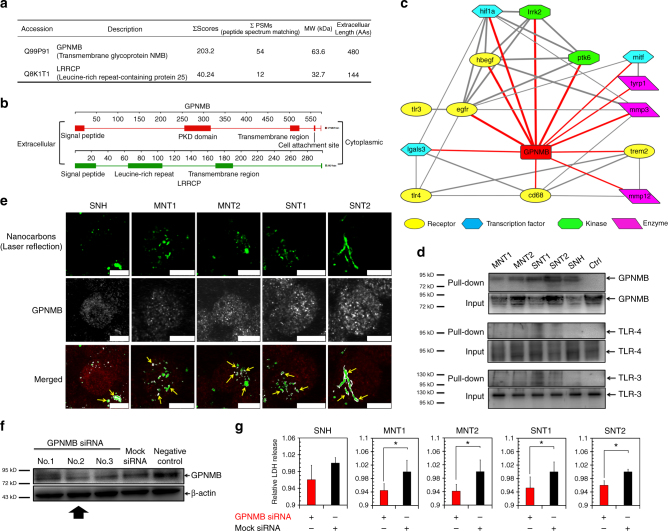
SNH triggered less GPNMB-regulated cell death. **a** The brief illustration of properties of two identified transmembrane proteins, GPNMB and LRRCP. **b** Schematic of topology and structure domain analysis of GPNMB and LRRCP. **c** Protein–protein interaction network centered with GPNMB detected by STRING software. The direct interactions with GPNMB were exhibited with red color. The thickness of bond represented the strength of interaction. **d** “Pull down” assay of GPNMB, TLR-3 and TLR-4 proteins based on immunoprecipitation-like strategy after nanocarbon incubations. GPNMB showed higher affinity with nanocarbons than Toll-like receptors. **e** Co-localization images of intracellular nanocarbons with GPNMB proteins detected by CLSM. Yellow arrows and white colors denoted the co-localization regions. Scale bar: 10 μm. **f** Western blot analysis of GPNMB expression after targeted GPNMB siRNA transfections with different sequences. As shown by black arrow, No.2 exhibited the greatest silence effect and was selected for subsequent cytotoxic test. **g** Cytotoxicity detections of different nanocarbons after GPNMB knockdown via LDH release assay (*n* = 3). Data were presented as mean ± s.d. Statistical significances were calculated by Student’s *t*-test: *p < 0.05

It was noteworthy that only two transmembrane receptors, glycoprotein nonmetastatic melanoma protein B (GPNMB) and leucine-rich repeat containing protein 25 (LRRCP), were identified to bind with all five nanocarbons based on MS technology. According to the evaluation by Mascot software, GPNMB had greater values of score and peptide spectral matching (PSM) compared to LRRCP (Fig. 9a), indicating the higher identification accuracy and more binding amount for GPNMB. The topological analysis further showed that GPNMB had a longer chain of extracellular domain (480 AAs in 552 AAs), which occupied the dominant proportion of whole sequence of GPNMB (Fig. 9b). It meant that the affinity of GPNMB with nanocarbons mostly relied on its extracellular domain, and this might be more aligned with the real nano-protein interaction.

In spite of the known heparin or integrin binding functions, GPNMB was recently reported to highly express on macrophages and regulate the uptake of microbes or cellular debris for inflammation repair^47,48^. The protein–protein interaction network based on STRING analysis (Fig. 9c) further showed that GPNMB had strong correlations with proteins including MITF, CD68, and TREM2, all of which function in lysosome stress, lysosome metabolism, and cell–pathogen interactions^49,50^. So it was indicated that GPNMB might function like pattern recognition receptors (PRRs) to recognize nanocarbons with high affinity and regulate their degradation in phagosome/lysosome pathway.

Here, “pull down” assay based on immunoprecipitation (IP) technology was used to evaluate the affinity of GPNMB with nanocarbons and the canonical PRRs like Toll-like receptor-3 (TLR-3) and TLR-4 was set as reference. It was showed in Fig. 9d that the immunoblots of GPNMB were clearly observed after the treatment with nanocarbons, whereas no corresponding bands of TLR-3 and TLR-4 were detected, therefore demonstrating the greater interaction of GPNMB with nanocarbons. Likewise, CLSM was utilized to clarify whether nanocarbons could interact with GPNMB in the real cellular uptake process. As illustrated in Fig. 9e, plenty of GPNMB proteins bound and wrapped the intracellular nanocarbons (yellow arrows), revealing the significant co-localization.

Last but not least, it was very essential to identify the correlation of GPNMB with nanocarbon-induced cell death. In this regard, we specifically reduced the expression of GPNMB in macrophages via gene silencing technology by choosing No.2 siRNA sequence (Fig. 9f). As showed in Fig. 9g, the reduction of intracellular GPNMB levels partly decreased the nanocarbon-induced cytotoxicities, thus demonstrating the involvement and regulation role of GPNMB proteins in cell death caused by SNH and CNT. Notably, due to the lower binding abundance, GPNMB knockdown in SNH group induced much less cytotoxicity reduction than CNT groups, suggesting the better biocompatibility of SNH in molecular level. Generally, our findings demonstrated that GPNMB could recognize intracellular nanocarbons, facilitate nano-membrane interaction and induce GPNMB-associated lysosome stress. Distinctively, SNH triggered less GPNMB-regulated cell death compared to CNT.

## Discussion

Generally, a careful investigation on nanocarbons was performed from their cell internalization to intracellular location by transportation analysis, and from cytotoxicity to protein-initiated cell death pathway by nanotoxicology evaluation. All the examinations on SNH were compared in parallel with four types of nanotubes throughout the study, in an attempt to demonstrate the difference in safety and biocompatibility between the novel and conventional forms of nanocarbons. With great emphasis on the mechanism of nanocarbon-induced cell death, each point or key event was investigated by multiple approaches and techniques. Consequently, we could illuminate the molecular principle of these nanocarbons in terms of cascade toxicity issues from initiation to final cell death.

**Fig. 10:**
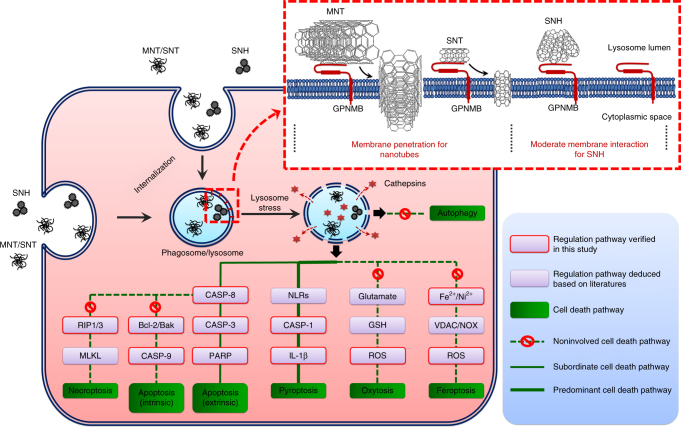
Schematic of cell death mechanisms induced by SNH and CNT. Nanocarbons interacted with test cells in assemblies and internalized through phagocytosis pathway. GPNMB was found with high affinity to nanocarbons, which strengthened the nano-membrane interaction, initiated lysosome stress, induced the leakage of hydrolases, triggered the activation of pyroptosis and extrinsic apoptosis pathways, and finally led to the death of macrophages. Distinctively, the less cellular uptake, lower nano-protein interaction, and moderate membrane disturbance endowed SNH with hypotoxicity, predicting a promising future for the potential biomedical applications for SNH. *SNH* single-walled carbon nanohorns, *MNT* multiple-walled carbon nanotubes, *SNT* single-walled carbon nanotubes, *GPNMB* glycoprotein nonmetastatic melanoma protein B, *CASP-1/3/8/9* caspase-1/3/8/9, *NLRs* Nod-like receptors, *RIP1/3* receptor-interacting protein kinase 1/3, *Bcl-2* B cell lymphoma‑2, *Bak* apoptosis regulator BAK, *PARP* poly (ADP-ribose) polymerase, *MLKL* mixed lineage kinase domain-like, *GSH* glutathione, *ROS* reactive oxygen species, *VDAC* mitochondrial voltage-dependent anion channel, *NOX* NADPH oxidase

Based on the series of careful studies, it was concluded that SNH possessed better nanosafety compared to CNT, which in fact guaranteed a very promising future for the potential application of SNH in the field of biomedicines. As seen in the insert of Fig. 10, owing to different morphological features, SNH and CNT exhibited distinct patterns of nano-protein and nano-membrane interactions, which generated the difference of interplay in degree, and eventually the differences in nanotoxicity between them. GO analysis based on functional annotation and hierarchical clustering revealed that proteins bonded on nanohorns differed from nanotubes. More precisely, less amount of GPNMB was detected on SNH than that of CNT, and it endowed the nanohorns the lower degree of GPNMB-reinforced biomembrane disturbance, which was the key to organelle-initiating cell death^51^, such deciphering the favorable biocompatibility of SNH compared to nanotubes. Besides, spherical SNH could initiate cell death via a mechanism similar to CNT, which was properly attributed to the identical structure composition of nanocarbons.

Up to now, we are able to draw a clear panorama on the nano/bio interaction between nanocarbons and model cells. As shown in Fig. 10, nanocarbons could be endocytozed by macrophage via phagocytosis. GPNMB, as a membrane protein highly expressed in endosome/lysosome system, reinforced the nano/membrane interplay which originally was rather weak, and induced lysosome stress through its high affinity with nanocarbons. The sequent membrane disturbance led to the leakage of acid hydrolases from phagosome/lysosome, resulted in the activations of CASP-8/CASP-3 and CASP-1 pathways, and finally facilitated the rupture of macrophages to death. During the process of nanocarbon-induced cell death, pyroptosis and apoptosis functioned simultaneously, although pyroptosis as one type of necrosis dominated, while the involvement of necroptosis, oxytosis/ferroptosis, autophagy, and MPT-dependent necrosis were basically excluded.

Importantly, the role of GPNMB was discovered here in the nano/protein interaction studies. Accordingly to previous reports, GPNMB could extensively express myeloid cells including monocyte, macrophagocyte, and myeloid-derived suppressor cells, and directly bind with SD-4 receptor in T cells to induce immunosuppressive effect^52,53^. Since nanocarbons could interact with GPNMB with high affinity as we found in this study, they might competitively block the binding of GPNBM with SD-4, consequently presenting or enhancing the immunological function of nanocarbons. In this regard, it is supposed that this type of membrane protein might become a potential inherent target for nanocarbon-based immunotherapy.

In conclusion, the difference between SNH and CNT was demonstrated here in a series of cascade cytotoxicity issues, and multiple investigations confirmed the improved safety and biocompatibility of single-walled nanohorns over four types of established carbon nanotubes. For the first time, a transmembane glycoprotein (GPNMB) was found to bind with nanocarbons and initiate the toxicity events. With distinct morphological features, SNH and CNT exhibited different nano-protein and nano-membrane interplay in pattern and degree, and eventually dissimilar nanotoxicity. Interestingly, such differences in nanocarbon-induced cytotoxicity were basically dependent on the geometry of their single-construct unit but not their dispersive forms in medium or intracellular concentration.

## Methods

### Materials and cells

Single-walled carbon-nanohorns (SNH) were obtained from Qingdajiguang technology (Beijing, China). SNH were prepared by arc discharge method. The fabricated nanohorn monomer was scaled with 2–5 nm in diameter and 10–20 nm in length. Multi-walled carbon nanotubes (MNT) and single-walled carbon nanotubes (SNT) were provided by DK nanotechnology (Beijing, China). Both MNT and SNT were prepared by chemical vapor deposition method. Two types of pristine nanotubes with different lengths for MNT and SNT respectively were chosen as comparisons in study. MNT1 and MNT2 had the same pipe diameter range of 20–30 nm but with distinct lengths (0.5–2 μm for MNT1 and 2–5 μm for MNT2). SNT1 and SNT2 also possessed the identical caliber in 1–2 nm whereas showed difference in lengths (0.5–2 μm for SNT1 and 2–5 μm for SNT2). Polyclonal antibodies used for immunofluorescence, immunoblotting, and immunoprecipitation, including caspase-3 p17 antibody (H-60, sc98785, 1:1000), caspase-8 p18 antibody (H-134, sc7890, 1:1000), caspase-9 p10 antibody (H-83, sc7885, 1:1000), RIP3 antibody (H-43, sc135170 1:1000), RIP antibody (H-207, sc7881, 1:1000), TLR-3 antibody (N-14, sc8691, 1:1000), TLR-4 antibody (M-300, sc30002, 1:1000), GPNMB antibody (K-16, sc47006, 1:1000) were purchased from Santa Cruz Biotechnology (Dallas, Texas, USA). Rabbit monoclonal antibody to HMGB1 (ab79823, 1:5000) was obtained from Abcam (Cambridge, MA, USA). Caspase-1 polyclonal antibody (AP51035, 1:1000), tubulin polyclonal antibody (AP50855, 1:1000), beta-actin monoclonal antibody (AM1021B, 1:1000), and mouse IL-1β ELISA kit (KT205507) were purchased from Abgent (San Diego, CA, USA). Bcl-2 polyclonal antibody (AB112, 1:1000), Bak polyclonal antibody (AB016, 1:1000), LC-3B Polyclonal antibody (AL221, 1:200), Cytochrome C monoclonal antibody (AC908, 1:200), and PARP polyclonal antibody (AP102, 1:1000) were obtained from Beyotime (Jiangsu, China). Pharmacological inhibitors including Z-DEVD-FMK (Catalog No 1009), Z-VAD-FMK (Catalog No 1010) were purchased from Biovision (Milpitas, CA, USA). Bovine serum albumin (BSA) as suspending agent and glycine were obtained from Ameresco (Framingham, MA, USA). Necrostatin-1 (NEC-1) was provided by Cayman Chemical (Ann Arbor, MI, USA). Bafilomycin A (Baf A) and trypsin (0.25%) were purchased from Keygen Biotech (Jiangsu, China). N-Acetyl-l-Cysteine (NAC), Cytochalasin D (Cyto D), cholesterol, DiI, Coumarin-6, Nile red, Pyrene, 1,6-Diphenyl-1,3,5-hexatriene (DPH) were obtained from Aladdin (Shanghai, China). Magic RedⓇ Cathepsin B Detetion kit (Catalog No 937) was obtained from ImmunoChemistry Technologies (Bloomington, MN, USA). Fluorescein isothiocyanate-dextran (FITC-dextran, average mol wt 150,000) was purchased from Sigma-Aldrich (Darmstadt, Germany). The organelle-specific probes, including MitoTracker Green FM (M7514) and LysoTracker Red DND-99 (L7528) were obtained from Invitrogen (Waltham, MA, USA). Lipoid E80 egg phospholipids, as the main components of ALMs, were purchased from Lipoid GmbH (Ludwigshafen, Germany). GPNMB Trilencer-27 Mouse siRNA (Locus ID 93695, Catalog No SR417607) was obtained from OriGene (Rockville, MD, USA). Three unique 27mer siRNA duplexes with different sequences were contained in product. In detail, No 1: 5′-GGUAGACAACUGGAUAAAUAUGATG-3′; No 2: 5′-AGCAGACCCUUAAUAGCAACCAAGT-3′; No 3: 5′-GCAAUAUCGUCUAUGAGAAGAACTG-3′. MTT Cell Proliferation and Cytotoxicity Assay Kit, LDH Cytotoxicity Assay Kit, Apoptosis/Necrosis Assay Kit, Annexin V-FITC Apoptosis Detection Kit, Caspase 1 Activity Assay Kit and Enhanced ATP Assay Kit were obtained from Beyotime (Jiangsu, China).

Mouse macrophage cell line J774A.1 was obtained from National Infrastructure of Cell Line Resource (Beijing, China). Cells were cultured in standard conditions by using DMEM medium (high glucose concentration, 4.5 g l^−1^) supplemented with 10% fetal bovine serum (FBS, Gibco) and 1% penicillin–streptomycin solution. Cells were tested and determined to be free of mycoplasma contamination. Before the incubation with nanocarbons, cells were firstly primed with 100 ng ml^−1^ lipopolysacchride (LPS) at 37 °C for 3 h.

### Physicochemical characterizations of nanocarbons

The structure compositions of nanocarbons were firstly verified by Raman spectrometer (RM-1000, Renishaw, UK). In brief, about 2 mg nanocarbons were weighed respectively and placed on the object stage. The wavelength of irradiation laser was set as 514 nm and the scanning was ranged from 500 to 4000 cm^−1^. After the laser scanning for 150 s, the spectrum data were recorded based on the scattered signals.

Fourier transform infrared spectroscopy (FTIR, NEXUS-470, Thermo Fisher Nicolet, USA) was used to analyze the surface oxidation of nanocarbons. About 2 mg nanocarbons were weighed for the KBr pellet fabrication. During the detection, the scanning numbers of sample and background were both 32. The sampling gain and resolution were defined as 8.0 and 4.00 cm^−1^, respectively. The whole scanning range of wavenumbers was established from 900 to 4000 cm^−1^.

Thermal gravity analysis (TGA, Q600SDT, TA, USA) was utilized to evaluate the purities of nanocarbons. In brief, at least 5 mg nanocarbons were placed in a platinum disk in TGA instrument. The environment gas was set as air atmosphere. During the detection, samples were heated from 15 to 900 °C with the rate of 10 °C min^−1^. The gravities of samples were detected with the temperature change, and the Thermo Fishergravimetric curves of different nanocarbons were obtained.

For the elemental analysis, X-ray photoelectron spectroscopy (XPS, Escalab 250Xi, Thermo Fisher Scientific, MA, USA) was used in this study. At least 20 mg nanocarbons were weighed for XPS detection. In the measurement, the monochromator was Al Ka (1253.6 eV) X-ray, the power was set as 150 W, and the beam spot was adjusted to 500 μm.

Inductively coupled plasma mass spectrometry (ICP-MS) was utilized to determine the contents of common metal catalysts, including Zn, Cu, Ni, Co, Fe. In brief, at least 5 mg nanocarbons were placed in a 500 μl solution of 1% HNO3 for digestion. The digested solution was introduced into mass spectrometrer (PE-Sciex DRC, PerkinElmer, USA) to detect the contents of different metal catalysts.

### Preparation and characterization of nanocarbon dispersions in aqueous medium

Five nanocarbons, including SNH, MNT1, MNT2, SNT1, and SNT2, were dispersed in aqueous medium before the cellular investigations. In brief, 5 mg nanocarbons were added into 0.5% (w/v) BSA contained phosphate buffer solution (PBS) of 2 ml, respectively. BSA was utilized here for improving the dispersibilities of nanocarbons. The obtained suspensions were then dispersed using probe ultrasonic method with 200 W for 10 min. After centrifugation with 2000 × *g* for 10 min, the undispersible nanocarbon aggregations in pellets were discarded and the supernatants were performed again via ultrasonic dispersive technology with 200 W for 10 min in sterile condition. The finally obtained nanocarbon dispersions were used for the physicochemical characterizations and cellular investigations.

Dynamic light scattering analyzer (DLS, Malvern, Zetasizer Nano ZS) was utilized to measure the hydrodynamic diameters of nanocarbons in PBS. The detection temperature was set as 25 °C, dispersant viscosity was defined as 0.8872 cP, and material reference was determined as polystyrene latex. Nanocarbon dispersion with the concentration of 100 μg ml^−1^ was measured with a detection angle of 173°. The laser wavelength was defined as 633 nm (He–Ne). After running 12 times for one sample and constructing the correlation function, the mean sizes based on intensity, volume, and number parameters were obtained respectively by using cumulants analysis and non-negatively constrained least squares (NNLS) fitting algorithm.

During the transmission electron microscopy (TEM) detection, 100 μg ml^−1^ nanocarbon dispersions were dropped on a copper grid and monitored by a transmission electron microscope (JEM1400PLUS, JEOL, Japan) with an acceleration voltage of 200 kV. The TEM images were obtained with at least 5000 times magnification. More than 100 individual nanocarbons for each type of samples were analyzed d by Image-Pro Plus 6.0 (IPP) software to calculate the cross-section diameters. The diameter distributions and corresponding histograms were analyzed by SPSS 22.0 software.

### Confocal imaging of nanocarbons based on laser refection technology

Before microscopy investigation, cells were incubated with different nanocarbons (100 μg ml^−1^) at 37 °C for 24 h. After washing and fixation, cells were detected by confocal laser scanning microscope (Leica, TCS, SP8, Germany) based on laser refection (LR) technology. The phase contrast images were first captured. Because of the obvious light-absorbing effect, it was shown sharp contrast for nanocarbons compared to the nonopaque cellular structures. Meanwhile, the reflection signals from nanocarbons were collected by setting 633 nm laser as the irradiation source. The detector channel was adjusted over the wavelength of the selected laser and the AOBS mode was set to allow 10–15% of laser into the collection channel. The laser reflection intensities of nanocarbons and corresponding gray-scale values under phase contrast condition were simultaneously detected to verify the feasibility of LR technology. Additionally, to evaluate the correlation of mass concentrations with LR signals, nanocarbon dispersions with different concentrations were directly detected by CLSM by fixing nanocarbons in solid-state acrylamide gel. In detail, 30% acrylamide solution (acrylamide: bisacrylamide = 29:1) was homogeneously mixed with nanocarbon dispersions. Ammonium persulfate and TEMED (tetramethylethylenediamine) were then added to induce the gel polymerization. The gel that dispersed with nanocarbons was detected by CLSM via LR technology and re-constructed in 3D mode by Imaris software. The obtained LR intensities based on 3D digital images were then compared with mass concentrations to evaluate the correlations.

### SEM imaging of cellular interactions with nanocarbons

The cell membrane interactions of nanocarbons during cellular uptake process were detected by SEM. In brief, cells were cultured on coverslips and incubated with different nanocarbons (100 μg ml^−1^) at 37 °C for 24 h. Cells were washed by PBS and fixed with 2.5% glutaraldehyde. Then the fixed cells were sequentially dehydrated in 25, 40, 60, 80, 90, 100% ethanol and finally dried in a desiccator. The coverslips were placed on SEM stage and sputter coated with gold. The obtained samples were finally viewed by SEM (JSM-5600LV, JEOL, Japan).

### Cellular uptakes of nanocarbons

The internalization of nanocarbons by cells was investigated by confocal laser Raman microscopy (CLRM), confocal laser scanning microscopy (CLSM) and transmission electron microscopy (TEM) respectively in study.

J774A.1 cells were cultured in a sterile glass bottom dish and incubated with five nanocarbon dispersions (100 μg ml^−1^) at 37 °C for 24 h. After incubations, cells were washed with PBS for three times to eliminate the adsorption of nanocarbons on cell surface and fixed with 3.7% paraformaldehyde. For CLRM investigation, cells were firstly detected via bright-field mode. By defining appropriate confocal plane, cells were then scanned with the laser of 633 nm in CLRM (HORIBA Jobin Yvon, France). The integrated Raman scattering signals ranged from 100 to 3000 cm^−1^ were collected and compared with control (cells without nanocarbon incubations). In terms of CLSM investigation (Leica, TCS, SP8, Germany), the nanocarbon-incubated cells were stained with DiI at 37 °C for 1 h for the labeling of lipid bilayers. After washing with PBS, the intracellular nanocarbons were detected under the laser irradiation of 633 nm condition based on LR technology and exhibited in green pseudo color. The digital 3D reconstruction images were also captured via X-Y-Z tomography scanning mode and analysis by Imaris software. For TEM observation, nanocarbon-incubated cells were digested by trypsin (0.25%) at 37 °C for 10 min and performed with cell scraper to capture single-cell suspension. After washing with PBS and fixation in 2.5% glutaraldehyde overnight at 4 °C, the collected cells were postfixed with 4% OsO_4_, dehydrated in an ethanol series (25, 40, 60, 80, 90, 100%), embedded in Epon and cut into slices. The obtained ultrathin sections were viewed by TEM (JEM1400PLUS, JEOL, Japan) with an acceleration voltage of 200 kV.

### Cytotoxicities of nanocarbons

For the cytotoxic invetigations of nanocarbons, J774A.1 cells were cultured in a 96-well plate and incubated with nanocarbons with different concentrations at 37 °C for 24 h. The modified MTT assay was utilized to evaluate the toxic effects of nanocarbons. In brief, different nanocarbon dispersions were aspirated after incubations. Cells were washed by PBS and re-cultured with fresh culture mediums of 200 μl. 20 μl MTT solution (5 mg ml^−1^, DMSO solvent) was added into each well and further incubated with cells for 4 h at 37 °C. After the formation of sufficient formazan in cells, the culture medium was discarded and 150 μl DMSO was added to fully dissolve formazan at 25 °C for 10 min under shaking condition. To eliminate the interference of residual nanocarbons to absorption spectrometry, the obtained formazan solutions in DMSO were further centrifuged in 12,000 × *g* for 30 min. The supernatants were transferred into a new 96-well plate and the absorbance of each well was detected by microplate reader (Thermo Fisher Multiskan™ FC, USA) at 570 nm wavelength.

Lactate dehydrogenase (LDH) release assay kit was also used to detect the cytotoxicities of nanocarbons. As the manufacturers’ instructions, nanocarbons with different concentrations were incubated with cells that cultured in 96-well plates at 37 °C for 24 h. Then the nanocarbon contained culture mediums were aspirated and centrifuged in 12000 × *g* for 30 min to remove the residual nanocarbons in medium. An aliquot of 120 μl supernatant was transferred to a new 96-well plate and added with 60 μl LDH detection solution. After incubation at room temperature for 30 min, the absorbance of each well was detected by microplate reader (Thermo Fisher Multiskan™ FC, USA) at 490 nm wavelength.

The difference of cell deaths caused by five nanocarbons was evaluated by flow cytometry based on PI labeling. In the investigation, cells were incubated with nanocarbons (100 μg ml^−1^) at 37 °C for 24 h. After washing with PBS, cells were performed by cell scraper to capture single-cell suspensions, labeled by PI solution (2 μg ml^−1^) at 4 °C for 20 min, and finally detected by flow cytometry system (FACS Calibur, BD, NJ, USA) at the PI channel. The data were performed by FlowJo 7.6 software.

The DNA damage was detected by comet assay (DNA damage detection kit) based on single-cell gel electrophoresis method (SCGE). In brief, cells were incubated with nanocarbons (100 μg ml^−1^) at 37 °C for 24 h. Single-cell suspensions with the volume of 100 μl (1 × 10^5^ ml^−1^) were collected and added to 100 μl of 0.5% agarose gel. The agarose was then dropped onto a comet slide in melting condition, stored at 4 °C for 10 min and immersed in lysis buffer at 4 °C for 2 h. After washing with alkaline solution for three times, gel electrophoresis was performed at 25 V for 30 min. The slide was washed with Tris-HCl (pH 7.5) for three times, stained with PI solution (2 μg ml^−1^) in dark for 10 min to label DNA and finally detected by fluorescence microscope (IX 71, Olympus, Japan). The obtained images were further digitally enhanced by Image-Pro Plus 6.0 (IPP) software.

### Influence evaluations of different inhibitors on cytotoxicities

Different pharmacological inhibitors were utilized in study to detect the cell death mechanism caused by nanocarbons. In these investigations, cells were incubated with nanocarbons (100 μg ml^−1^) at 37 °C for 24 h. Different pharmacological inhibitors, including cytochalasin D (10 μM), bafilomycin A (100 nM), Z-VAD-FMK (20 μM), Z-DEVD-FMK (100 μM), Necrostatin-1 (100 μM), KCl (5 mM), glycine (10 μM), NAC (5 mM) were added into culture mediums during nanocarbon incubations. LDH release assay was used to detect the influences of different inhibitors on the cytotoxicities of five types of nanocarbons.

### Immunoblotting and immunofluorescence analyses of cell death-associated proteins

The cleavages of PARP, activations of caspase-1, caspase-3, caspase-8, caspase-9, and the expressions of RIP (RIP1, RIP3), Bak, Bcl-2 were all tested by immunobloting method. In brief, cells were incubated with nanocarbons (100 μg ml^−1^) at 37 °C for 24 h. After incubations, cells were then performed to capture single-cell suspensions. By centrifugation in 600 × *g* and washing with PBS for three times, cell pellets were lysed by RIPA lysis buffer (containing 1% PMSF (phenylmethylsulfonyl fluoride)) at 4 °C. The lysate proteins in supernatant were then obtained by centrifuging in 12,000 × *g* for 10 min. 10–20 μg lysate proteins were separated through 10% sodium dodecyl sulfate-polyacrylamide gel electrophoresis (SDS-PAGE) and transferred to polyviinglidene difluoride (PVDF) membranes by Mini-PROTEAN^Ⓡ^ Tetra system (Bio-Rad, Hercules, CA, USA). The obtained PVDF membranes that loaded with proteins were blocked with 5% BSA in TPBS (PBS plus 0.05% Tween-20) and incubated with primary antibodies at 4 °C overnight with slow shaking. After washing with TPBS, membrane was then blotted by horseradish peroxidase (HRP) conjugated secondary antibodies at room temperature for 2 h. The resulted protein bands were illuminated by chemiluminiscence kit (BeyoECL Plus, Beotime, Jiangsu, China) and detected by Gel Illuminator (ChemiDoc XRS, Bio-Rad, Hercules, CA, USA).

The intracellular distributions and locations of HMGB1, Cytochrome C, LC-3B, and GPNMB were detected via immunofluorescence method. Cells were cultured on coverslips and incubated with different nanocarbons (100 μg ml^−1^) at 37 °C for 24 h. After washing by PBS, fixation with 3.7% paraformaldehyde, permeabilization using 0.1% Triton-PBS and blocking with 5% BSA-PBS, cells were incubated with primary antibodies at 4 °C overnight, and then treated by matching fluorescent secondary antibodies at 37 °C for 2 h. The obtained cells were further stained with Hoechst series or DAPI dyes to label cell nuclei. The coverslips after whole treatment were placed on object stage and viewed by CLSM (Leica, TCS, SP8, Germany). The fluorescence channels were adjusted to match the spectrum of probes for protein detection, and LR signals were captured to label the intracellular nanocarbons.

### Comparison analyses of necrosis and apoptosis

The quantitative comparisons of necrosis and apoptosis caused by nanocarbons were evaluated via Annexin V-FITC Apoptosis Detection Kit and Apoptosis/Necrosis Assay Kit respectively. Cells were firstly incubated with nanocarbons (100 μg ml^−1^) at 37 °C for 24 h. As the instructions of Annexin V-FITC Apoptosis Detection Kit, cells were washed with PBS and scraped from culture plates to capture single-cell suspensions after nanocarbon incubations. The obtained cell suspensions were added with Annexin V-FITC-binding solution for 30 min at room temperature and further incubated with PI staining solution for 5 min before detection. The labeled cells were then analyzed by flow cytometry system (FACS Calibur, BD, NJ, USA). At least 10,000 cells were collected and tested for one sample. Data were treated by FlowJo 7.6 software.

Apoptosis/Necrosis Assay Kit was performed based on fluorescence microscopy analysis. Cells were cultured on coverslips and incubated with different nanocarbons (100 μg ml^−1^) at 37 °C for 24 h. After washing, cells were then incubated with Hoechst 33342/PI staining solution in living condition for 20 min at 4 °C. The double-labeled cells were carefully rinsed with PBS for three times to remove the unbound dyes and fixed via 3.7% paraformaldehyde. By placing coverslips on object stage, cells were viewed by fluorescence microscope (IX 71, Olympus, Japan). The obtained images were further digitally enhanced by Image-Pro Plus 6.0 (IPP) software. For quantitative comparisons, three independent experiments were performed and at least 1000 cells in each investigation were selected and analyzed by IPP.

### Caspase-1 and ATP activities assay

Intracellular caspase-1 activities were detected by specific assay kit based on the catalysis function of activated caspase-1 on substrate Ac-YVAD-*p*NA. As the manufacturers’ instructions, cells were firstly incubated with different nanocarbons (100 μg ml^−1^) at 37 °C for 24 h, and then treated with lysis buffer for 15 min in ice-bath after PBS washing to release intracellular caspase-1. The obtained lysates were centrifuged in 12,000 × *g* for 5 min. 50 μl supernatants were aspirated and mixed with detection buffer (40 μl) and Ac-YVAD-*p*NA solution (10 μl). The mixtures were incubated at 37 °C overnight and detected for absorbance at 405 nm wavelength by microplate reader (Thermo Fisher Multiskan™ FC, USA).

Intracellular ATP activities were detected based on the catalysis of firefly luciferases in assay kit. In brief, cells after nanocarbon incubations were lysed as the instructions and centrifuged in 12,000 × *g* for 5 min to capture supernatants. The detection solutions that contained firefly luciferases were added into supernatants for 5 min incubation at room temperature. Then the fluorescence intensities based on chemiluminiscence of the mixed solutions were measured by luminometer (FlexStation 3, Molecular Devices, CA, USA).

### Extracellular IL-1β determination and inflammation antibody array assay

Extracellular IL-1β was determined via ELISA (enzyme-linked immunosorbent assay). In brief, cells were incubated with different nanocarbons (100 μg ml^−1^) at 37 °C for 24 h. As the manufacturers’ instructions, the culture mediums after incubations were captured and transferred with the volume of 100 µl to ELISA plate. After incubations at 4 °C overnight with gentle shaking, each well was washed with PBS, added with 100 µl of biotinylated antibody for 1 h incubation, and further incubated with 100 µl of streptavidin solution for another 45 min at room temperature. After the last washing carefully, the ELISA plate was finally incubated with TMB one-step substrate reagent (100 µl) for 30 min and blocked by adding stop solution. The absorbance of sample in each well was detected by microplate reader (Thermo Fisher Multiskan™ FC, USA) at 450 nm wavelength.

Mouse inflammation antibody array C1 (RayBio@C-Series, RayBiotech, GA, USA) was utilized in study for the semi-quantitative detection of 40 inflammation related proteins in cell culture medium. According to the standard protocol, the obtained culture mediums (1 ml) after nanocarbon incubation were firstly added into each well of Incubation Tray that had been placed with Antibody Arrays. After the incubation at 4 °C overnight, the Array was washed with corresponding buffers. Then, 1 ml of prepared Biotinylated Antibody Cocktail were added into each well and incubated at room temperature for 2 h. After washing, 2 ml of 1 × HRP-Streptavidin was added into each well and incubated at room temperature for another 2 h. The finally Array membranes were washed again and transferred on a sheet of blotting paper. By adding developing buffer and incubating for 2 min, the obtained Array was then detected by a chemiluminescence imaging system.

### Mitochondrion and lysosome location analyses of nanocarbons

The location analyses of nanocarbons in mitochondria and lysosomes were performed by CLSM. Cells were cultured on coverslips and incubated with different nanocarbons (100 μg ml^−1^) at 37 °C for 24 h. After washing to remove extracellular nanocarbons, cells were stained with MitoTracker (1 nM) or LysoTracker (10 nM) respectively in living conditions as the manufacturers’ instructions. The LR signals were also detected to label intracellular nanocarbons. Based on the obtained confocal images, the co-localozation parameters (Pearson’s correlation and overlap coefficient) were measured via LAS AF Lite software (Leica, TCS, SP8, Germany) to evaluate the location features of nanocarbons.

### Lysosome membrane integrity analysis

Cathepsin B (CB) and FITC-dextran (FD) were selected as intrinsic and extrinsic content markers separately for lysosome labeling. Lysosome stress triggered membrane penetration, and caused the content leakage. Cells were first cultured on coverslips and incubated with different nanocarbons (100 μg ml^−1^) at 37 °C for 24 h. After that, cells were washed by PBS and added with CB-labeling reagent (10 μM) or FD (100 μg ml^−1^) for another 1 h incubations at 37 °C. Then the cells were immediately viewed by CLSM (Leica, TCS, SP8, Germany) in living condition. The fluorescence and LR signals were simultaneously collected in study and the surface plots based on heat maps were obtained via IPP analysis.

### Fabrication and labeling of artificial lipid membranes

Artificial lipid membranes (ALMs) were fabricated to detect the interactions of nanocarbons with lipid membrane. In preparation, 15 mg Lipoid E80 and 5 mg cholesterol were dissolved in chloroform. The obtained solution was evaporated in vacuum at 37 °C to form lipid film in flask. Then 2 ml distilled water was added and the obtained lipid suspension was vortexed intensively for 10 min to achieve the homogeneous dispersion. The obtained lipid suspension was placed in liquid nitrogen for 5 min to be frozen, and then transferred into water bath of 60 °C for 5 min to melt immediately. The freeze-thawing cycle was continuously operated for five times. After that, the lipid suspension was placed into a squeezer and extruded through a PTFE membrane with pores of 100 nm diameter. The squeeze was conducted back and forth for at least 20 times. The finally prepared liposomes were used for the subsequent investigations. During the fabrication of fluorescent ALMs, four probes, including nile red, pyrene, DPH and coumarin-6 was used to label ALMs. The mass ratio of fluorescent probes with lipid materials was set as 1:10,000. In preparation, both fluorescent probes and lipid materials were dissolved in chloroform to form lipid film. The subsequent procedure was the same as blank ALMs as described above.

### Interactions of ALMs with nanocarbons by spectrum analysis

Keeping the identical contact areas was the precondition in the interaction comparisons of different nanocarbons with ALMs. The mass concentrations of nanocarbons had to be adjusted and converted to the same surface areas for five types of nanocarbons. However, the inherent surface areas of nanocarbons could not truly reflect the membrane interactions, since the obvious accumulation characteristics of nanocarbons in aqueous medium blocked the contact of part materials with ALMs. By comparison, the apparent contact areas that calculated based on hydrodynamic radius (*R*_H_) of nanocarbon aggregations in medium might be more suitable for the interaction analysis. Although *R*_H_ measurement was based on the hypothesis that the particle should be uniform sphere, it could be an approximate value to characterize the diffusion and movement behaviors of nanocarbon aggregations in medium. Notably, it was the diffusion and movement that induced the interactions of nanocarbon aggregations with ALMs.

The hydrodynamic radiuses for five types of nanocarbons in medium were detected by DLS (Malvern, Zetasizer Nano ZS), in which the autocorrelation functions were measured by determining the light scattering fluctuations of nanocarbons. The intensity autocorrelation was indicated as below.

$$G_2\left( \tau \right) = \int\nolimits_0^\infty I\left( t \right)I\left( {t + \tau } \right)d\tau $$

In DLS, the field autocorrelation *G*_1_ was always first determined and then converted to intensity autocorrelation

$$G_2\left( \tau \right) = 1 + \gamma G_1(\tau )^2$$

To evaluate the consistency of surface area conversion based on *R*_H_, two different deconvolution algorithms, including cumulants analysis and non-negatively constrained least squares (NNLS) fitting algorithm, were performed in study.

In terms of cumulants analysis, which assumes an ideal field correlation function of identical diffusing spheres and fits the measured intensity correlation curve to a single exponential, the intensity autocorrelation was fitted as below.

$$G_2\left( \tau \right) = A[1 + B{\rm {exp}}( - 2{T}\tau + \mu _2\tau ^2)]$$

where *A* is the amplitude or intercept of the correlation function, *B* is the baseline, and Γ is the correlation decay rate. Thus the hydrodynamic radius (*R*_H_) was calculated using the Stokes-Einstein relationship.

$$R_{\rm{H}} = \frac{{kT}}{{6\pi \eta D}} = \left( {\frac{{kT}}{{6\pi \eta }}} \right)\left( {\frac{{q^2}}{{\mathrm{\Gamma }}}} \right)$$

And the approximate value of surface area was converted.

$$S = 4\pi (R_{\rm{H}})^2$$

Distinct from cumulants analysis, NNLS was represented as a summation of single exponential decays, where the factor *A*_i_ is the area under the curve for each exponential contribution, and represents the strength of that particular *i*th exponential function.

$$G_1\left( \tau \right) = {\sum} {A_i{\mathrm{exp}}\left( {{\mathrm{\Gamma }}_i\tau } \right)} $$

In NNLS algorithm, there was no single constant *R*_H_ value, which was weighted by multiple *R*_H_ values based on *Τ*_i_ calculation. So the approximate value of surface area was converted as below.

$$S = {\sum} {B_{i4\pi }\left( {R_{\rm{Hi}}} \right)^2}$$

According to cumulants and NNLS analyses, the mass concentrations of different nanocarbons were adjusted to keep identical apparent contact areas (S) during the membrane interaction investigations. The mass ratios were shown in Supplementary Table 1, e.g. the concentration of MNT1 need changing to 74 μg ml^−1^ to possess the same contact area with SNH in 100 μg ml^−1^ based on cumulants analysis.

During the spectrum analysis, different fluorescent ALMs (100 μg ml^−1^, 200 μl) were fabricated and incubated with five nanocarbons (200 μl) under the identical contact area condition at 37 °C for 2 h. The fluorescence emission spectrums for different probes were then detected by fluorospectrophotometer (FlexStation 3, Molecular Devices, CA, USA). The excitation wavelengths for nile red and pyrene-labeled ALMs were set as 552 nm and 334 nm separately. DPH-labeled ALMs were analyzed via fluorescence polarization technology to measure the lipid polarization (*P*). In the polarization mode, the excitation and emission wavelengths were set as 362 nm and 454 nm, respectively.

### Cryo-TEM imaging of interactions between ALMs and nanocarbons

Cryo-TEM was used to directly detect the interactions of ALMs with nanocarbons. ALMs with the concentration of 100 μg ml^−1^ were incubated with different nanocarbons (100 μg ml^−1^) at 37 °C for 2 h. The suspensions were then dropped on support grid and stand for 10 min at room temperature. After aspiration of excessive suspensions, the grid was immediately placed into liquid ethane that cooled by liquid nitrogen. The frozen grid was then transferred into sample holder immersed in liquid nitrogen and detected by Cryo-TEM in 200 kV condition.

### Fluorescence correlation spectroscopy analysis

FCS was used in study to explore the interaction features of ALMs with different nanocarbons. In brief, counarin-6 labeled ALMs (100 μg ml^−1^) were prepared and incubated with nanocarbons (100 μg ml^−1^) at 37 °C for 2 h. The mixed dispersions were then aspirated into a capillary tube with thickness of 0.17 mm. By placing on object stage, the capillary tubes were directly viewed and analyzed by CLSM (Leica, TCS, SP5 FCS, Germany) equipped with FCS detector. In the FCS investigation, the object lens was set as ×40 1.2NA water immersion and the laser was excited at 488 nm. The confocal plane was set to the center of capillary tube. After capturing the confocal images, the photons emitted from fluorophores were further detected by Avalanche photo diodes. During the signal acquisition, the sampling frequency was set as 20 kHz and the data were collected in time mode for 10 s irradiation. The obtained dada were loaded in ISS VistaFCS software (Leica Edition) for the fitting of autocorrelation curves and calculations of diffusion time (*t*_D_) and diffusion coefficients (*D*). In the fitting process, the fluorescence fluctuations were recorded in real time and the normalized autocorrelation was indicated as below.

$$G\left( \tau \right) = \frac{{ \langle \delta F(t) \times \delta F(t + \tau ) \rangle }}{{ \langle F(t) \rangle ^2}}$$

In this investigation, the autocorrelation curves of fluorescent ALMs were fitted in 3D diffusion mode of one or two components without non-fluorescing state by using equation as described below.

$$G_{3D}\left( \tau \right) = {\sum} {\frac{\rho }{{1 + \left( {\frac{\tau }{{\tau _D}}} \right)}} \cdot \sqrt {\frac{1}{{1 + \left( {\frac{\tau }{{\tau _D \cdot \kappa ^2}}} \right)}}} } $$

*ρ* represented the amplitude contributed by a molecular species. *τ*_D_ was the diffusion time and *κ* denoted the structure parameter defined as the ratio of the axial beam size *z* and radius *w* of the effective volume.

### Separation, extraction, and verification of high-affinitive proteins with nanocarbons

Separation and extraction of high-affinitive proteins with nanocarbons were utilized via two strategies. First, the cultured cells were lysed by RIPA lysis buffer (containing 1% PMSF) at 4 °C. The obtained cell lysate (protein content 10 mg ml^−1^) was then incubated with nanocarbons (100 μg ml^−1^) at 4 °C for 12 h to induce the protein binding. Second, cells were incubated with nanocarbons (100 μg ml^−1^) at 37 °C for 24 h beforehand. After washing, cells were lysed to release the intracellular nanocarbons. No matter through which method, the obtained mixtures that contained nanocarbons and proteins were separated by centrifuging in 4000 × *g* for 10 min and washed with PBS for three times. After aspirating the supernatant, the nanocarbon pellets were added with 1% SDS/PBS and intensively shaken for 1 h to desorb the surface binding proteins. By centrifuging in 12,000 × *g* for 10 min, the eluted proteins in supernatant were then blotted by SDS-PAGE (Bio-Rad, Hercules, CA, USA).

Like immunoprecipitation, certain proteins that bond on nanocarbon surface could be “pulled down” during the separation by centrifugation. To verify the affinities, specific antibodys were used to detect the bound proteins. As the separation and extraction method described, 10–20 μg desorbed proteins were separated by SDS-PAGE (10% acrylamide concentration), transferred to PVDF membranes, and blocked with 5% BSA in TPBS. After washing, the PVDF membranes were incubated with primary antibodies at 4 °C overnight with slow shaking and blotted by HRP conjugated secondary antibodiess at room temperature for another 2 h. The resulted protein bands were illuminated by chemiluminiscence kit (BeyoECL Plus, Beotime, Jiangsu, China) and detected by Gel Illuminator (ChemiDoc XRS, Bio-Rad, Hercules, CA, USA).

### Proteomics identification of high-affinitive proteins with nanocarbons

To identify the desorbed proteins in eluent through proteomics, these proteins were first digested using tube-gel method (2 μl sample, 14.25 μl water, 45 μl of Tris-HCl buffer (pH 8.8), 35 μl of 30% acrylamide solution, 1 μl 10% SDS, and 2.5 μl of 10% ammonium persulfate). The gel was then fixed with 50% methanol, 12% acetic acid for 30 min at room temperature and cut into small pieces. After dehydration with acetonitrile (ACN), reduction with TCEP, alkylation with iodoacetamide and washing with 50% ACN /50 mM NH_4_HCO_3_ buffer. The gel was rehydrated with 100 μl trypsin (10 ng μl^−1^) in 25 mM NH_4_HCO_3_ buffer at 4 °C for 2 h and then incubated at 37 °C overnight. The obtained peptides mixture was extracted with ACN/5% trifluoroacetic acid (TFA) (v/v, 2/1) twice and lyophilized for subsequent identification.

In the LC-MS/MS investigation based on LFQ method, the peptides (10 µl) were loaded on a C18 pre-column (Easy-column C18-A1, 100 μm I.D. × 20 mm, 5 μm, Thermo Fisher Scientific, MA, USA) and separated by nano-LC-MS/MS using an Easy-LC nano-HPLC (Thermo Fisher Fisher Scientific, MA, USA). During the separation, H_2_O/TFA was set as mobile phase A and ACN/TFA was mobile phase B. The flow rate was adjusted to 300 nl per min, and the gradient was set as from 5 to 30% for 90 min, from 30 to 50% for 10 min, then from 50 to 100% for 10 min, and held at 100% for 10 min. Mass spectrometric analysis was performed by using an LTQ Orbitrap Velos pro or Q-Exactive HF (Thermo Fisher Scientific, MA, USA). MS/MS spectra were obtained in a data-dependent collision-induced dissociation (CID) model or high energy collision dissociation (HCD) model, and the full MS was acquired from *m/z* 350 to 2000 with resolution 60,000. The top 15 most intense ions were selected to for MS/MS.

Raw data were analyzed using Proteome Discoverer v1.4.1.14 (Thermo Fisher Scientific, MA, USA) or MaxQuant software (version 1.5.6.0). MS/MS spectra were searched against UniProt Mouse database (released march 2015, 53289 sequences) using the Andromeda search engine with the following settings: trypsin cleavage; fixed modification of carbamidomethylation of cysteine; variable modifications of oxidation of methionine; a maximum of two missed cleavage. The false discovery rate was calculated by decoy database searching. LFQ was performed in MaxQuant or Proteome Discoverer. The Minimum ratio count for LFQ was set to 2, and the match-between-runs option was enabled. Other parameters were set as default. Up- and downregulated proteins were defined based on a significantly altered protein ratio (*p*-values < 0.01). Significance p-values were calculated using Perseus software (version 1.6.0, http://www.perseus-framework.org). A two fold change in expression and a *p*-value of 0.01 were used as a combined threshold to define biologically regulated proteins.

### Proteomics determination of total proteins after cellular incubation of nanocarbons

Before the proteomics analysis, cells were first incubated with nanocarbons at 37 °C for 24 h. After incubations, cells were then performed to capture single-cell suspensions. By centrifugation in 600 × *g* and washing with PBS for three times, cell pellets were lysed by RIPA lysis buffer (containing 1% PMSF (phenylmethylsulfonyl fluoride)) at 4 °C. The lysate total proteins in supernatant were obtained by centrifuging in 12,000 × *g* for 10 min. Protein samples (200 μg) from each group were then performed as the manufacturer’s protocol for FASP (Filter Assisted Sample Preparation, Mann). First, samples were added into Vivacon@500 filtrate tube (Cat No. VNO1HO2, Sartorius Stedim Biotech) and mixed with 200 μl of 8 M urea dissolved in 0.1 M Tris-HCl (pH 8.5,). After centrifugation at 14,000 × *g* for 15 min at room temperature, 10 μl of 0.05 M TCEP in water was added to the filter and incubated at 67 °C for 10 min. The obtained sample was then incubated with 10 μl of 20 mM IAA for 30 min in darkness. After washing twice with 50 mM NH_4_HCO_3_, 4 μg of trypsin (Promega, Madison, WI, USA) was added. The protein/enzyme ratio was 50:1. The total protein sample was incubated overnight at 37 °C and the released peptides were collected by centrifugation and lyophilized.

To improve the resolution and sensitivity for MS/MS determination, the digested peptides were separated via high pH reverse phase chromatography before LC-MS/MS detection using the Dionex Ultimate 3000 Micro Binary HPLC Pump system. The peptide sample was dissolved in with buffer A (60 μl, 0.1% NH_3_.H_2_O in water) and loaded onto a 2.1 × 150 mm Waters XBridge BEH130 C18 column containing 3.5 μm particles (Waters, Milford, MA, USA). The flow rate was set as 230 μl per min and the gradient was modulated as 5% buffer B (0.1% NH_3_.H_2_O in 80% ACN, pH = 10) for 5 min, 1–15% buffer B for 15 min, 15–25% buffer B for 10 min, 25–55% buffer B for 10 min, and finally 55–95% buffer B for 5 min. The system was then maintained in 95% buffer B for 5 min before equilibrating with 1% buffer B for 8 min prior to the next injection. Elution was monitored by measuring the absorbance at 214 nm, and fractions were collected every 2.2 min. A total of 27 fractions containing eluted peptides were pooled into 10 fractions based on peptide density and vacuum-dried for nano-LC-MS/MS analysis (Thermo Scientific, MA, USA). The peptide solution was injected (5 μl) at a flow rate of 5 μl min^−1^ onto a pre-column (Easy-column C18-A1, 100 μm I.D. × 20 mm, 5 μm, Thermo Fisher Scientific, MA, USA) and then performed on a C18 column (Easy-column C18-A2, 75 μm I.D. × 100 mm, 3 μm, Thermo Fisher Scientific) at a flow rate of 300 nl per min with a 60 min gradient of 2 to 40% ACN in 0.1% formic acid. Mass spectrometric analysis was performed by using an LTQ Orbitrap Velos pro or Q-Exactive HF (Thermo Fisher Scientific, MA, USA). MS/MS spectra were obtained in a data-dependent CID model or high energy collision dissociation (HCD) model, and the full MS was acquired from *m/z* 350 to 2000 with resolution 60,000. The top 15 most intense ions were selected to for MS/MS. Then the subsequent MS analysis was performed via the same strategy as described above in “Proteomics identification of high-affinitive proteins with nanocarbons” section.

### Bioinformatics analysis

For the obtained protein data, the correlation between two independent investigations and the Venn diagrams for different types of nanocarbon groups were analyzed by Perseus (Version 1.6.0, http://www.perseus-framework.org) software. The functional classification and statistical overrepresentation test based on GO analysis were performed using Panther software (Version 12.0, http://www.pantherdb.org/). *p*-values were corrected for multiple testing using Bonferroni procedure. The gene-annotation enrichment analysis and functional annotation clustering were performed by DAVID Bioinformatics Resources 6.8 (https://david.ncifcrf.gov/home.jsp). The protein–protein interaction network based on GPNMB was obtained via String (Version 10.5) and the interaction scores beyond 0.400 were uploaded to Cytoscape (Version 3.5.0) for visualization. The topology analyses of GPNMB and LRCCP were performed via UnitProKB and drawn by DNAstar software. The hierarchical clustering of identified proteins from different nanocarbon groups was performed by Perseus (Version 1.6.0) software.

### Influences of GPNMB silence on cytotoxcities caused by nanocarbons

GPNMB knockdown was achieved by specific siRNA transfection. In brief, cells were cultured to reach 60–80% confluence. Before the transfection, siRNA against GPNMB was diluted in Opti-MEM Medium (Invitrogen, Waltham, MA, USA) and mixed with Lipofectamine RNAiMAX Reagent (Invitrogen, Waltham, MA, USA) as the instructions to prepare siRNA-lipid complex. The complex with the final siRNA concentration of 10 pmol was then added into culture medium and incubated with cells for 24–48 h at 37 °C. The verification of GPNMB silence was detected by immunoblotting method as described above. After validation, the most efficacious siRNA was selected for the subsequent cytotoxic investigation. In brief, cells were silenced by GPNMB siRNA and incubated with nanocarbons (100 μg ml^−1^) at 37 °C for 24 h. After incubation, the cytotoxic effects of different nanocarbons were detected using MTT Cell Proliferation and Cytotoxicity Assay Kit and LDH Cytotoxicity Assay Kit.

### Statistics analysis

All data in study were shown in mean ± s.d. The Kolmogorov–Smirnov (K–S) statistical testing was first utilized to assess the normality of data. If the test met the demand, the unpaired Student’s *t*-test (two-tailed) was used for two-group comparison. If not, the Wilcoxon test was then utilized for comparison.

### Data availability

The mass spectrometry proteomics data have been deposited to the ProteomeXchange Consortium (http://proteomecentral.proteomexchange.org) via the PRIDE partner repository^54^ with the dataset identifier (PXD009628) and PXD009635. The authors in this manuscript declare that all data supporting the findings of this study are available within the article and its supplementary information and data files.

## Electronic supplementary material

Supplementary Information

Description of Additional Supplementary Files

Supplementary Data 1

Supplementary Data 2

Supplementary Data 3

Supplementary Data 4

Supplementary Data 5
