# Supplementary material for: Single-walled carbon-nanohorns improve biocompatibility over nanotubes by triggering less protein-initiated pyroptosis and apoptosis in macrophages
Source: Nat Commun. 2018 Jun 19;9:2393. doi: 10.1038/s41467-018-04700-z (PMC6008334; doi:10.1038/s41467-018-04700-z)
Supplement: Supplementary file 1 — Supplementary Information [file 41467_2018_4700_MOESM1_ESM.pdf]

Supplementary Information

Single-walled carbon-nanohorns improve biocompatibility over nanotubes by triggering less protein-initiated pyroptosis and apoptosis in macrophages

Bing He[1,2,3], Yujie Shi[,2,4], Yanqin Liang[1,2,3], Anpu Yang[1,2,3], Zhipu Fan[1,2,3], Lan Yuan[5], Xiajuan Zou[5], Xin Chang[5], Hua Zhang[1,2,3], Xueqing Wang[1,2,3], Wenbin Dai[1,2,3], Yiguang Wang[1,2,3], Qiang Zhang[1,2,3]

Corresponding Author: Qiang Zhang
E-mail address: zqdodo@bjmu.edu.cn

1. Department of Pharmaceutics, School of Pharmaceutical Sciences, Peking University, Beijing 100191, China
2. State Key Laboratory of Natural and Biomimetic Drugs, Beijing 100191, China
3. Beijing Key Laboratory of Molecular Pharmaceutics and New Drug Delivery Systems, School of Pharmaceutical Sciences, Peking University, Beijing 100191, China
4. Department of Chemical Biology, School of Pharmaceutical Sciences, Peking University, Beijing 100191, China
5. Centre of Medical and Health Analysis, Peking University, Beijing 100191, China

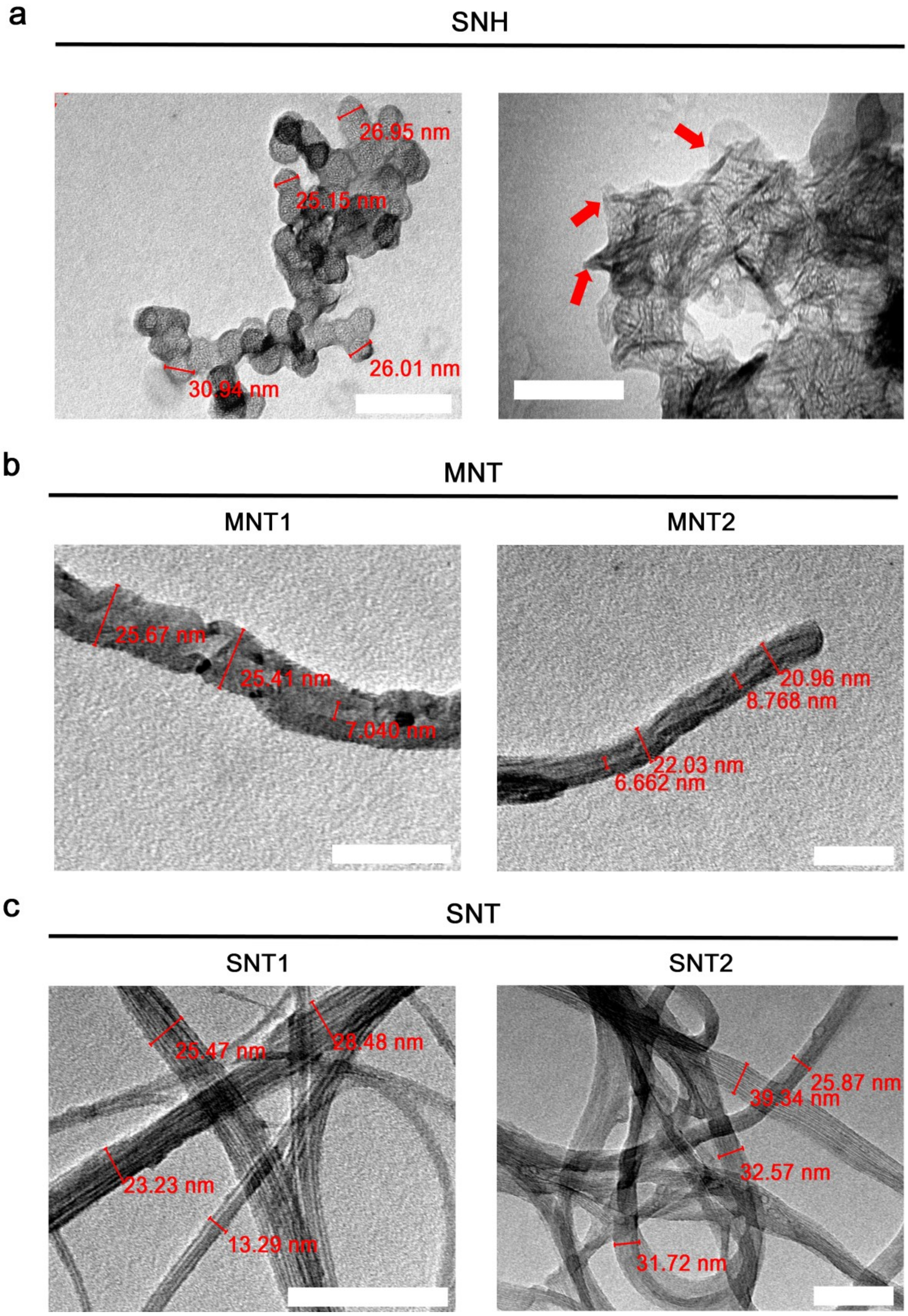

Supplementary Figure.1 Transmission electron microscopy (TEM) images of SNH (a) and four types of nanotubes ((b) and (c)) in cultural medium. Red arrows indicated the cone-like structures of SNH. The cross-section diameters of CNT were measured and shown in red color. Scale bar in (a): 100 nm. Scale bar in (b) and (c): 50 nm.

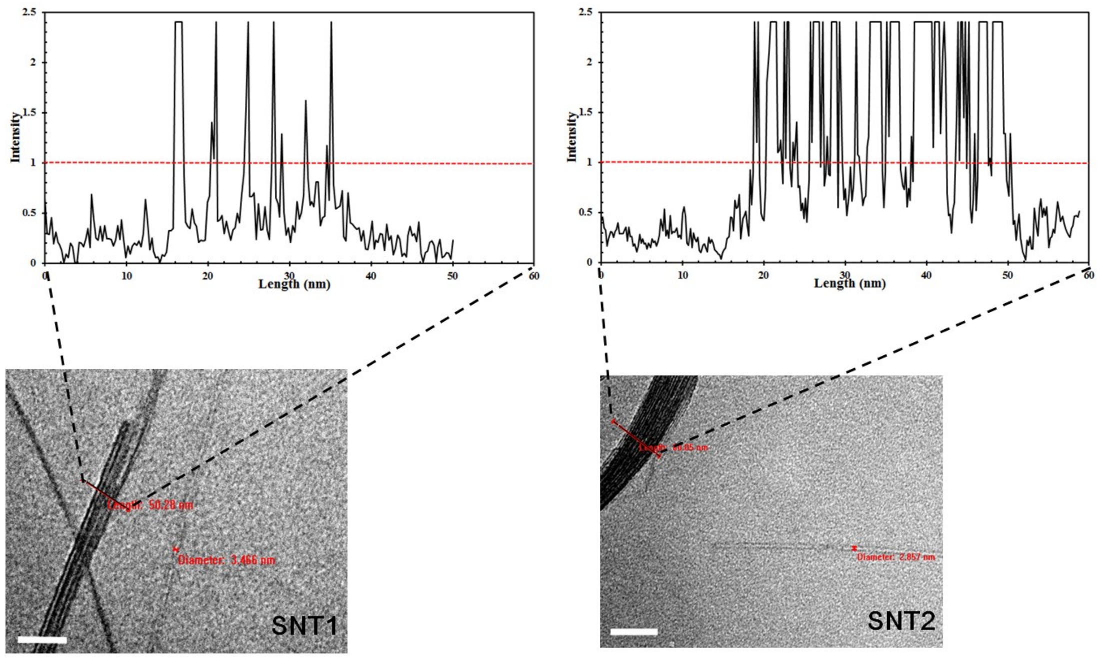

Supplementary Figure.2 TEM images with high magnifications of SNT1 and SNT2, showing the parallel assembly of nanotubes in medium. The corresponding grayscale intensity distributions exhibited that about 10-30 single nanotubes attracted each other in parallel to become bigger nanotube bundles. Scale bar: 50 nm.

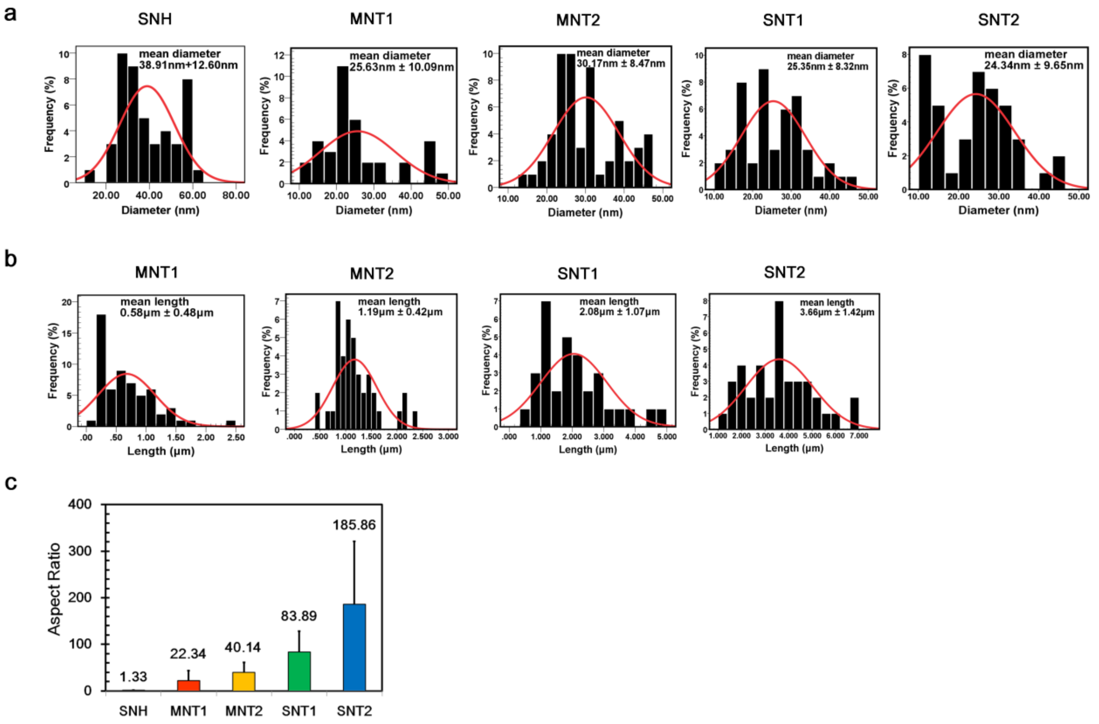

Supplementary Figure.3 (a) Distributional histograms of the cross-section diameters of nanocarbons based on transmission electron microscopy investigations. (b) Distributional histograms of the lengths of CNT based on transmission electron microscopy investigations. (c) Aspect ratio comparison of different nanocarbons. In (a) to (c), at least 30 individual nanocarbons were measured according to transmission electron microscopy results. The mean values in (a) to (c) were presented as means ± s.d.

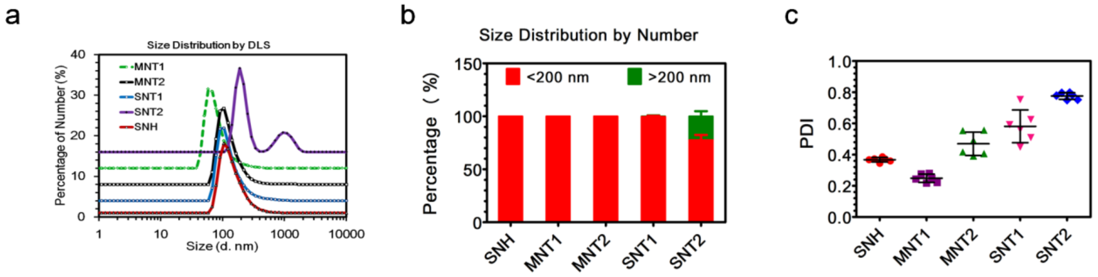

Supplementary Figure.4 (a) Size distributions of nanocarbons in cultural medium measured by dynamic light scattering (DLS) technique based on number percentage. (b) The size distribution characteristics of five types of nanocarbons based on quantity occupation.(c) The polydispersity index (PDI) of five types of nanocarbons in aqueous medium detected by DLS technique. In (b) and (c), six independent experiments were performed and the data were presented as means ± s.d.

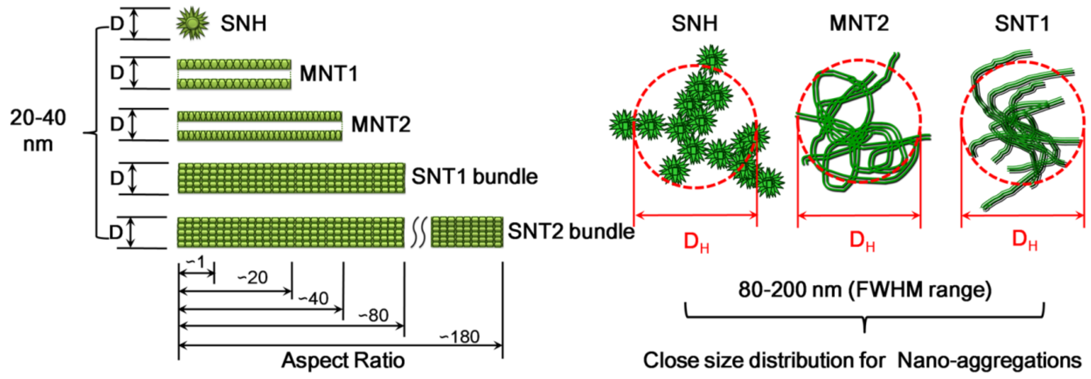

Supplementary Figure.5 Schematic of nanoscale comparison of different nanocarbons based on structure units and aggregation forms in cultural medium. Five nanocarbons showed similar distribution features on the cross-section diameters (D). SNH, MNT2 and SNT1 further exhibited aggregative characteristics with close size range (D_H: hydrodynamic diameter) based on FWHM (full width at half maximum) measurement.

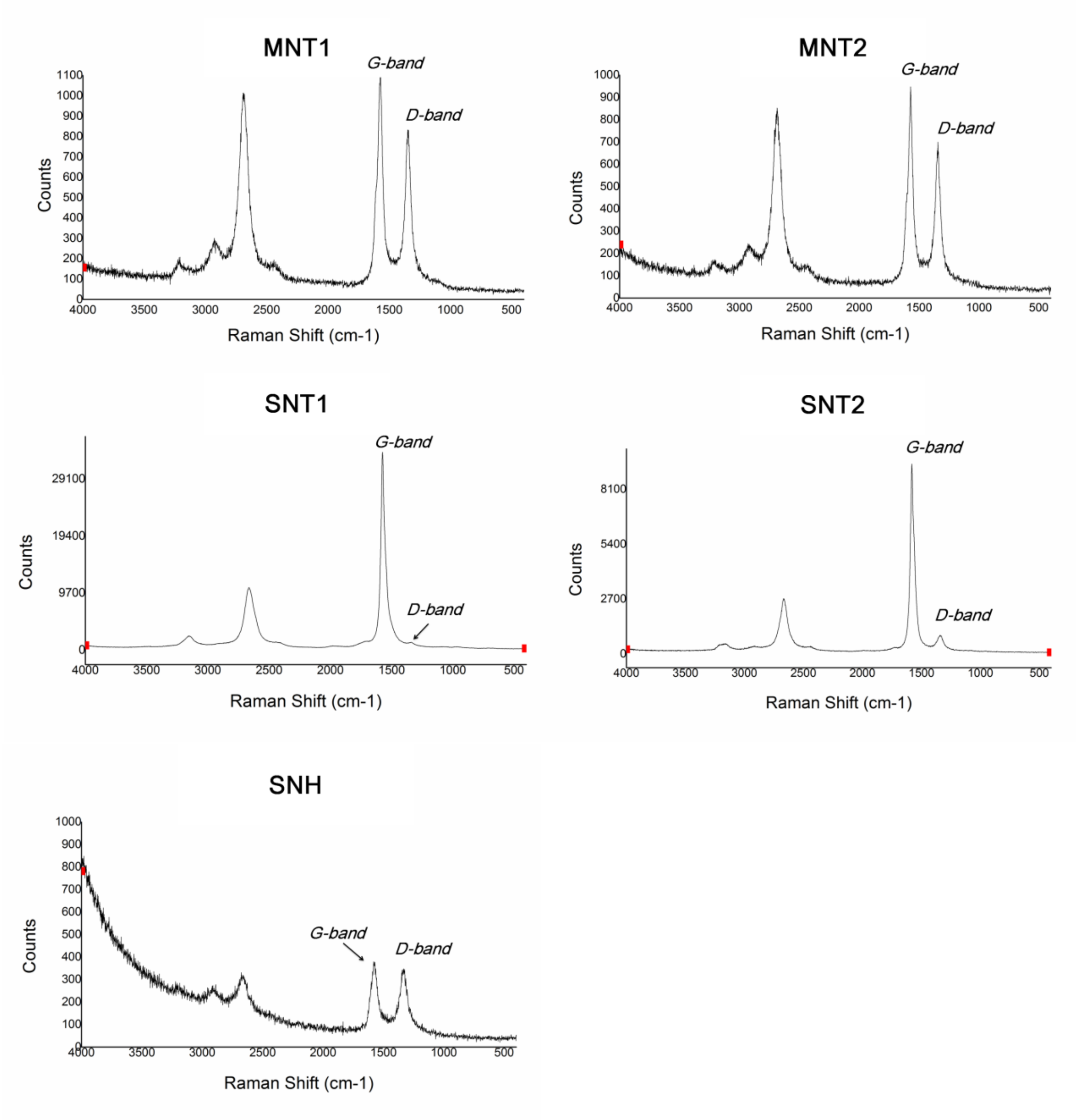

Supplementary Figure.6 Raman spectrums of different nanocarbons.

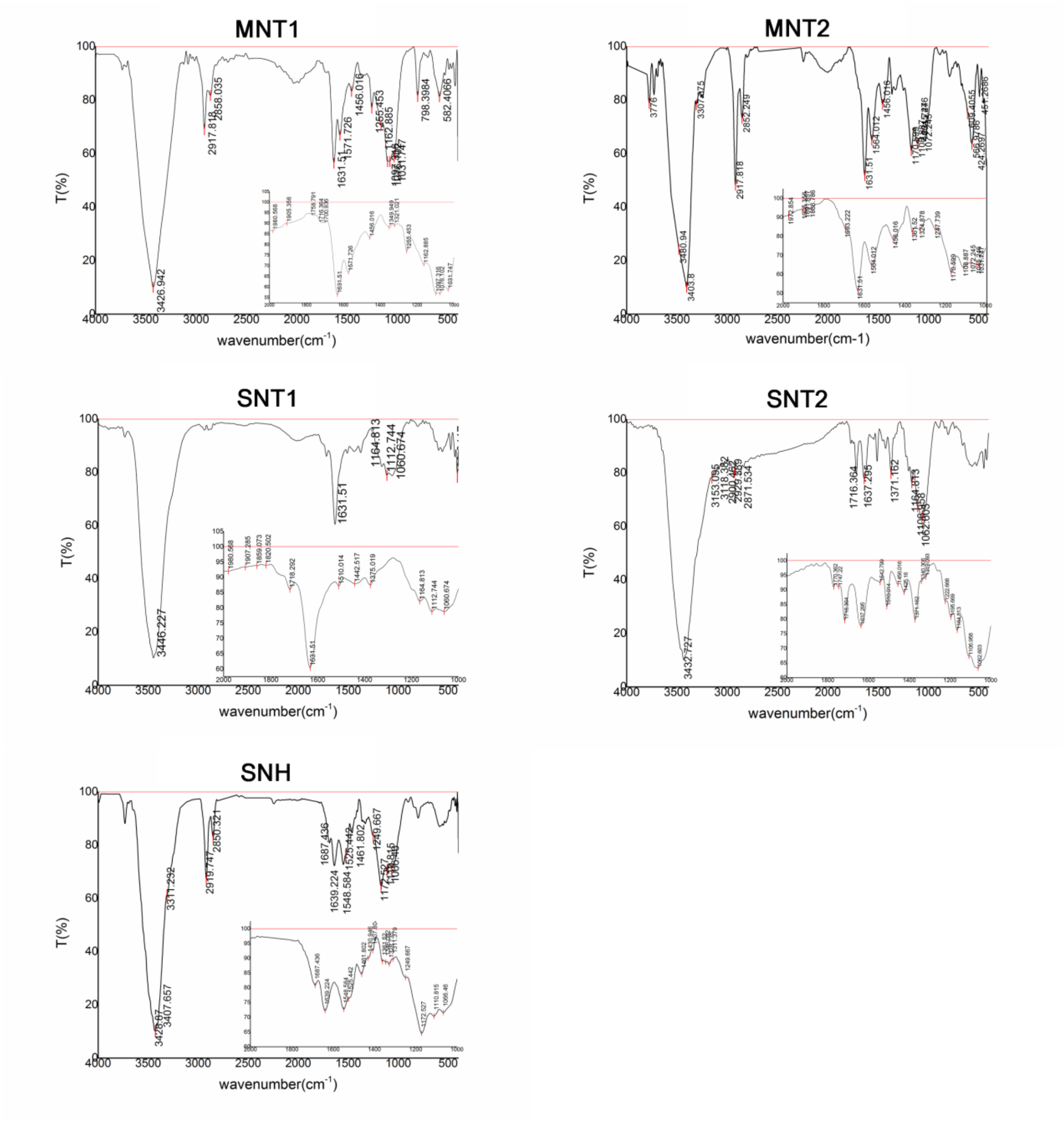

Supplementary Figure.7 Infrared spectrums of different nanocarbons based on Fourier transform infrared spectroscopy (FTIR).

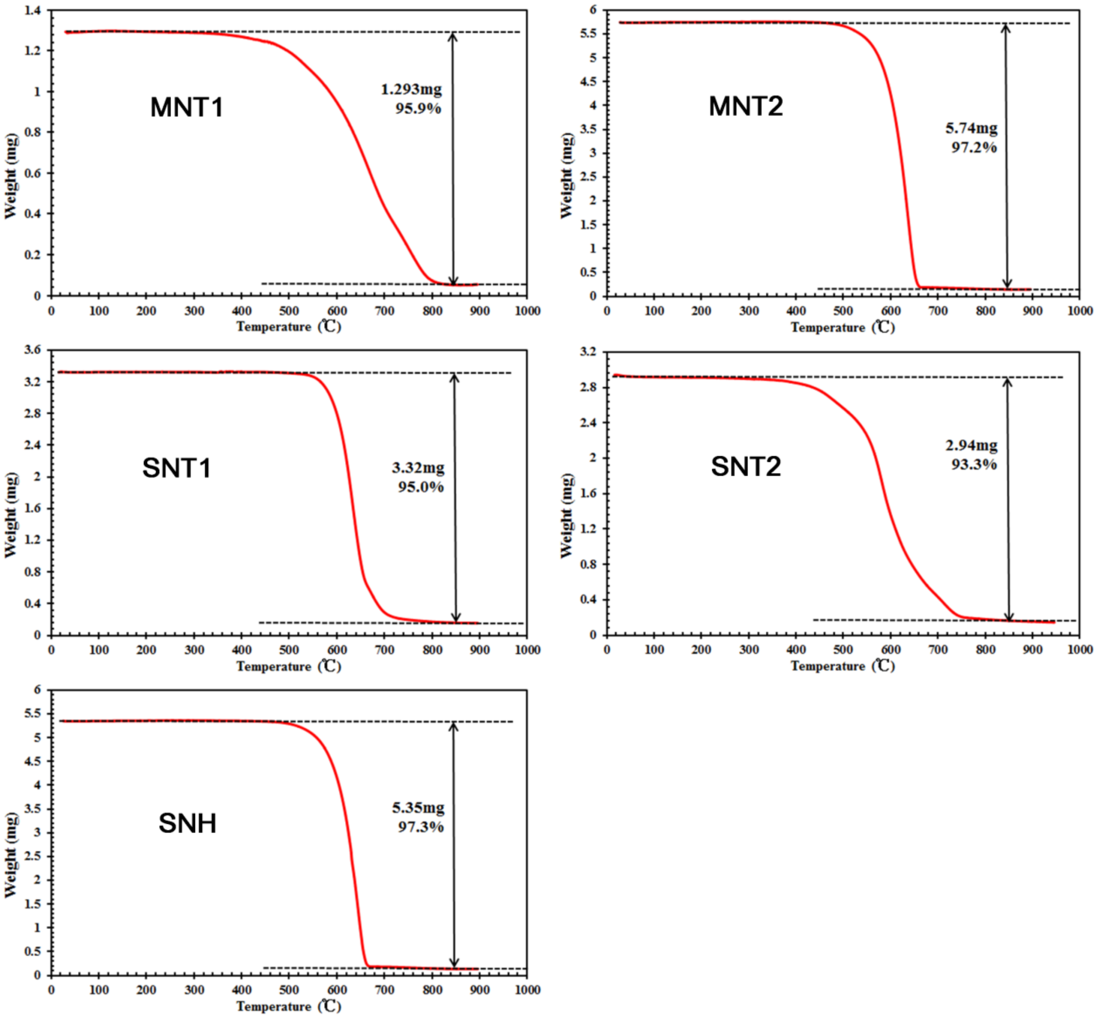

Supplementary Figure.8 Thermogravimetric (TGA) analysis of nanocarbons. The weight losses of SNH and nanotubes under air atmosphere were all above 93%, indicating the high purity for all five nanocarbons.

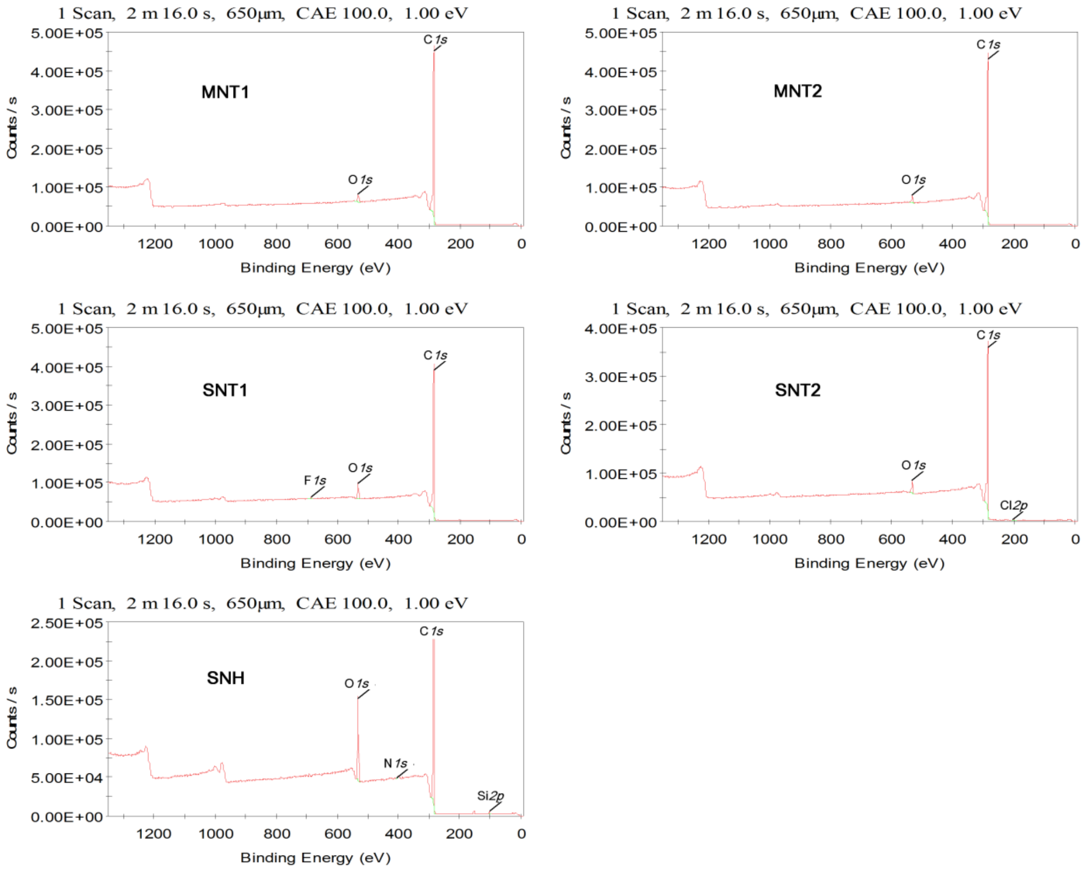

Supplementary Figure.9 Elemental analysis of nanocarbons based on X-ray photoelectron spectroscopy (XPS).

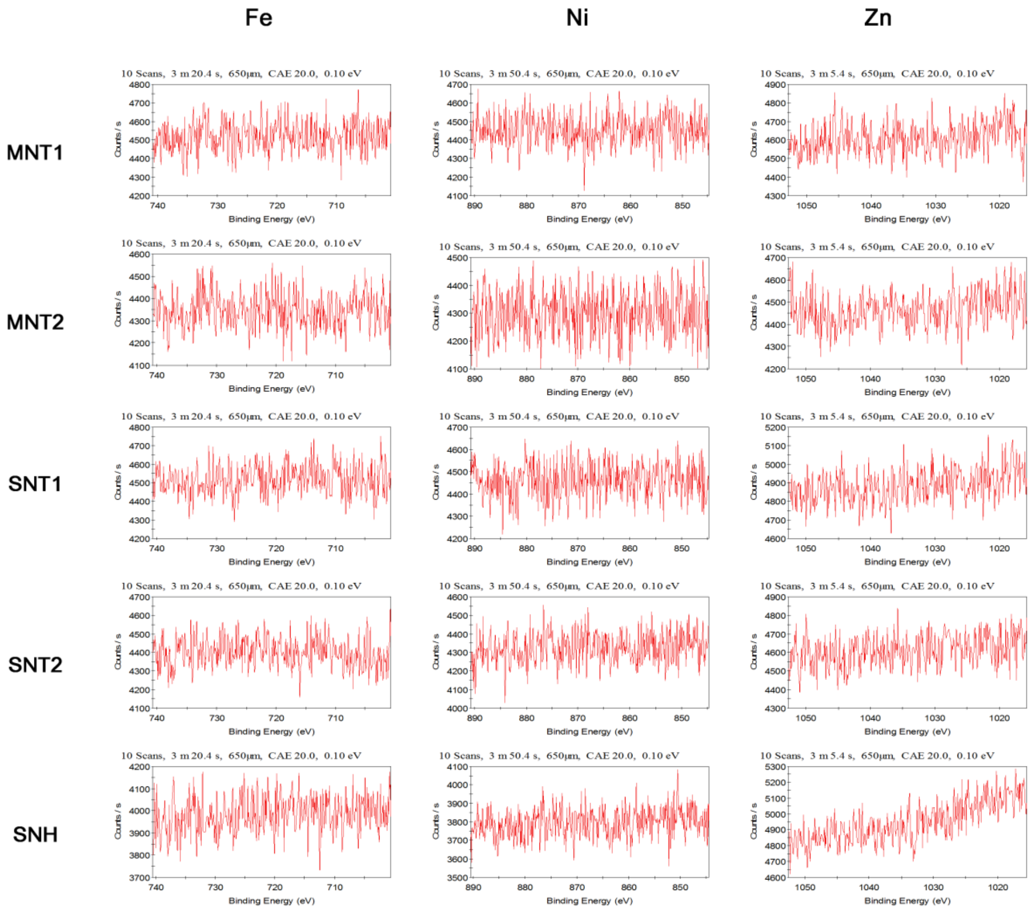

Supplementary Figure.10 Residual metal catalyst detection (Fe, Ni, Zn) of nanocarbons based on X-ray photoelectron spectroscopy (XPS).

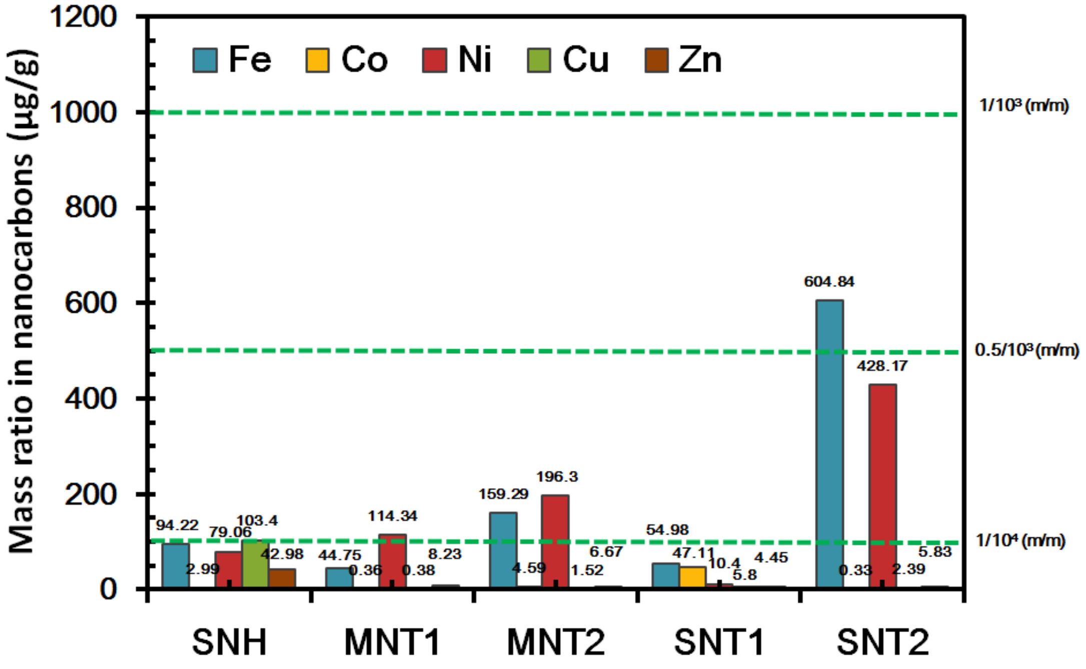

Supplementary Figure.11 Inductively coupled plasma mass spectrometry (ICP-MS) for metal catalyst detection. The mass ratios of usual metal catalyst components (Zn, Cu, Ni, Co, Fe) in SNH and nanotubes were all below 1‰, some were even below 0.1‰.

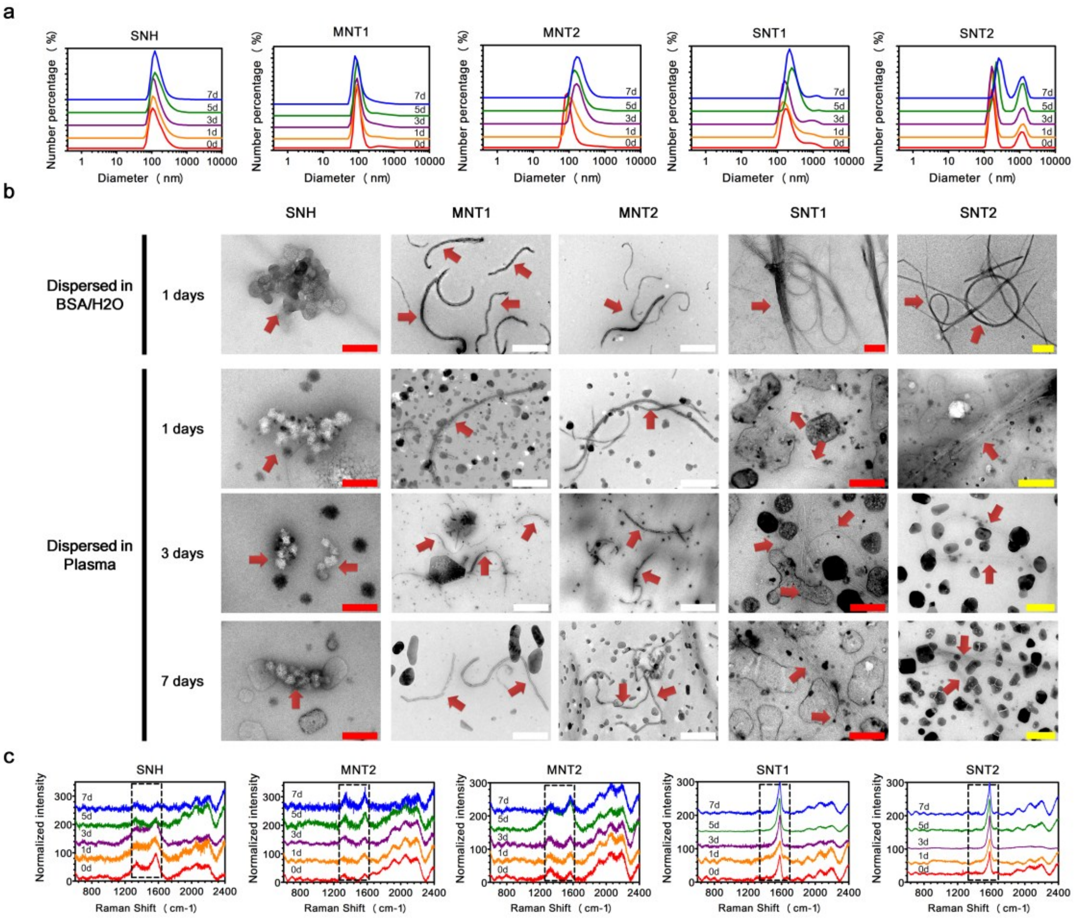

Supplementary Figure.12 The dispersity and stability analyses of nanocarbons in mouse plasma. (a) The particle size distributions of nanocarbons in mouse plasma detected by DLS at room temperature for 7 days. (b) TEM images of different nanocarbons dispersed in plasma for 7 days. The nanocarbon dispersions in BSA/H_2O were monitored as control by TEM. Scale bar in SNH and SNT1 groups: 100 nm (red color). Scale bar in MNT1 and MNT2 groups: 500 nm (white color). Scale bar in SNT2 groups: 200 nm (yellow color). (c) The Raman spectrums of different nanocarbons in mouse plasma detected by CLRM at room temperature for 7 days. The characteristic peaks (D and G bands) of nanocarbons were shown in dotted line diagrams.

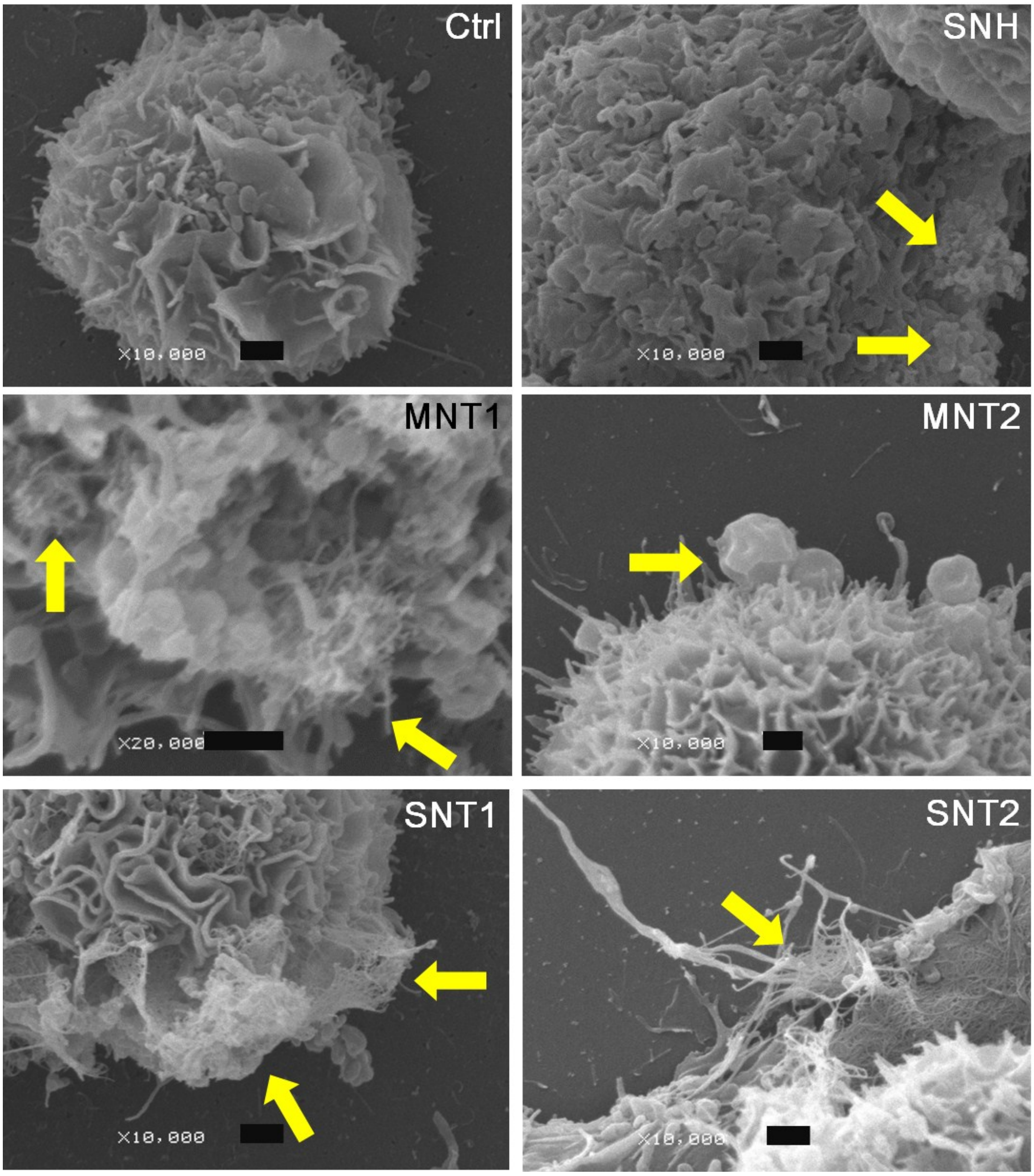

Supplementary Figure.13 SEM images of the surface adsorptions of different nanocarbons on cell membrane. Yellow arrows in images showed that all five nanocarbons adhered to cell membrane and assembled to form aggregations. Scale bar (black color): 1 μm.

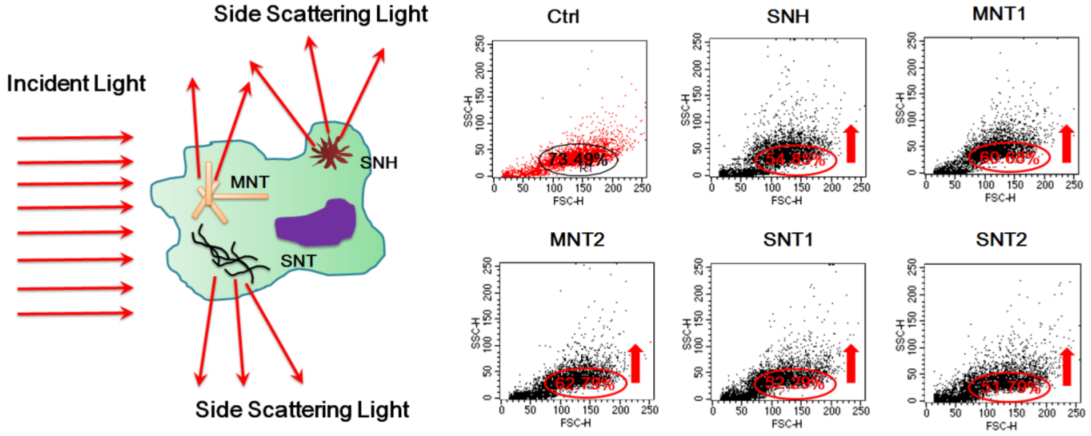

Supplementary Figure.14 Flow cytometry analysis of cells after nanocarbon incubations based on scattering light signal detection, indicating the cellular entry capabilities of nanocarbons.

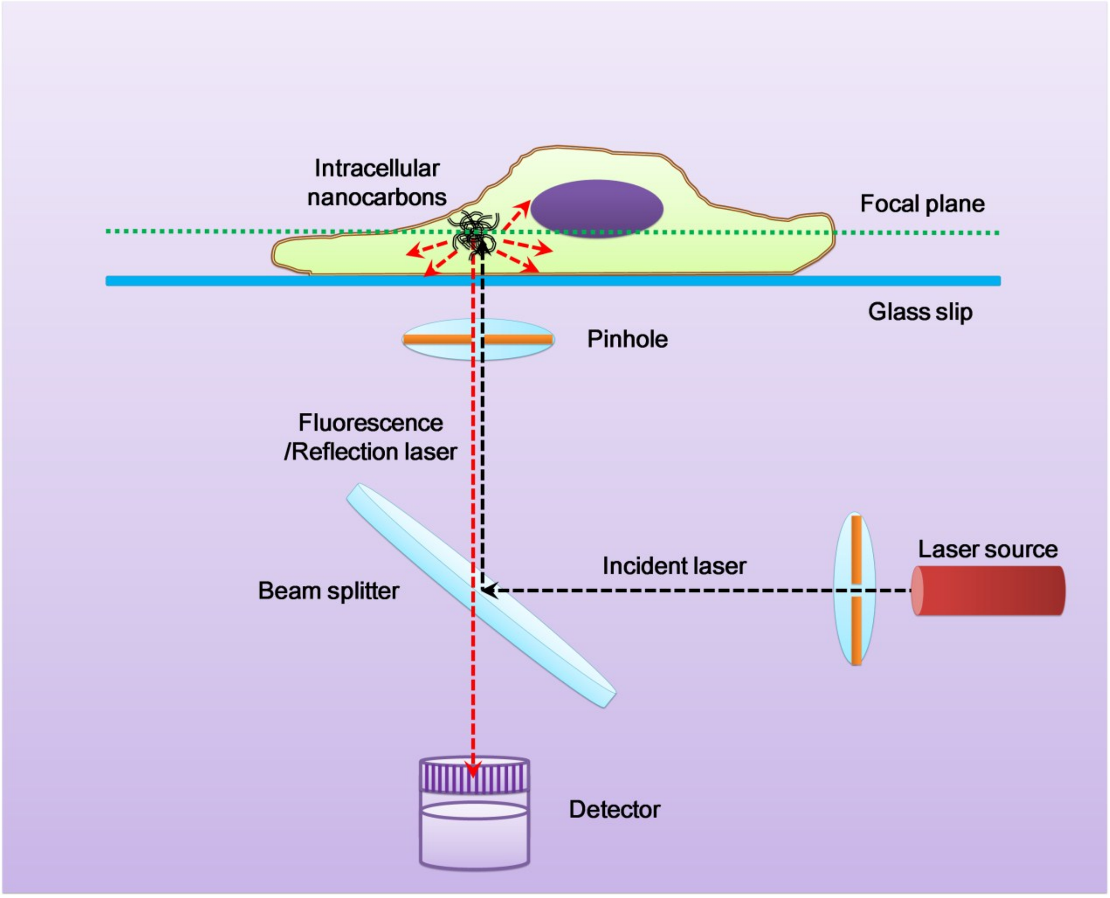

Supplementary Figure.15 Schematic of laser reflection (LR) technology based on CLSM for the detection of intracellular nanocarbons.

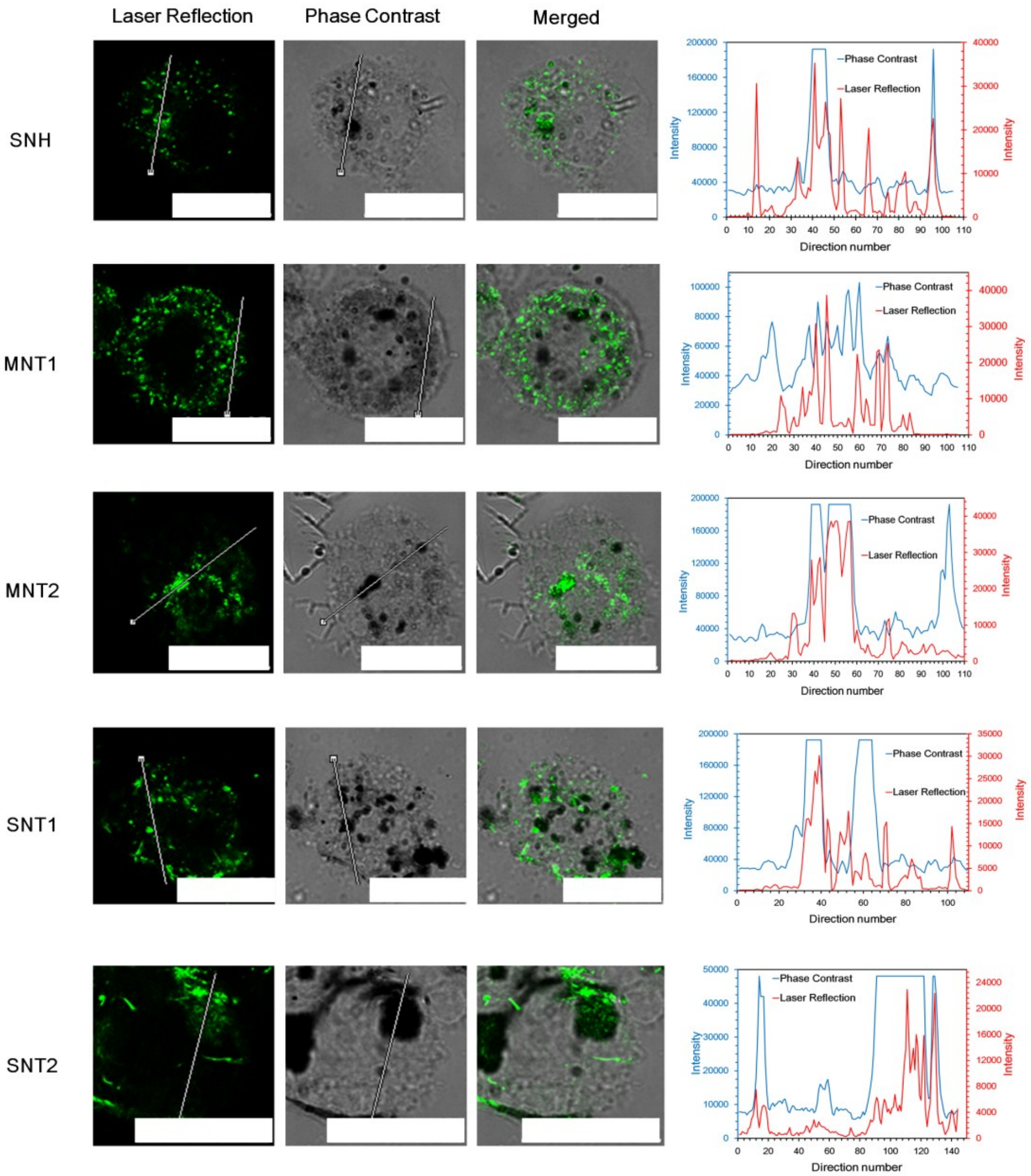

Supplementary Figure.16 Confocal images of intracellular nanocarbons based on LR detection. Intracellular nanocarbons were marked with green pseudo color. Scale bar (white color): 25 μm. The intensity distributions along the vector lines showed that LR signals exhibited good consistency with the corresponding gray scale intensities of nanocarbons, demonstrating the feasibility of LR technology.

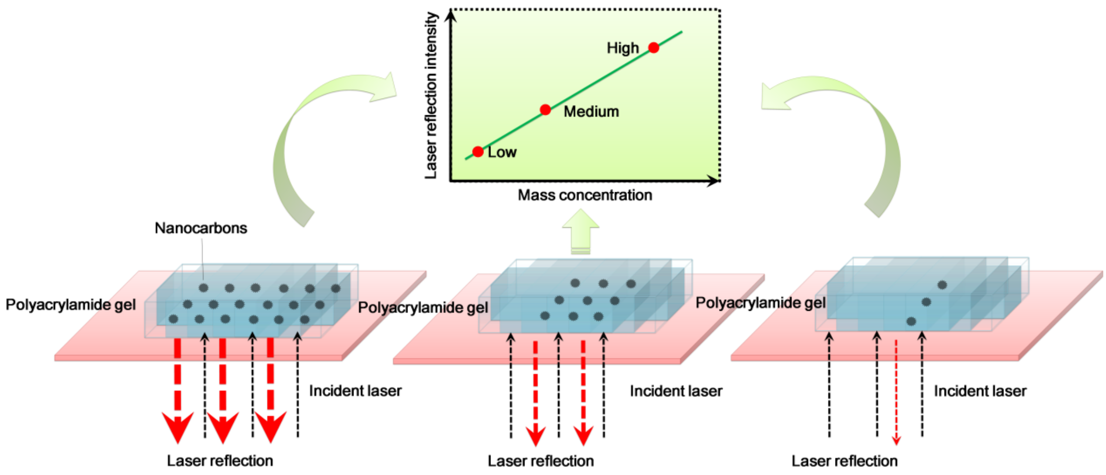

Supplementary Figure.17 Schematic of the linear correlation of LR signals with the real mass concentrations. Nanocarbons with different concentrations were fixed in polyacrylamide gel. LR signal intensities were detected meanwhile and compared with corresponding mass concentrations.

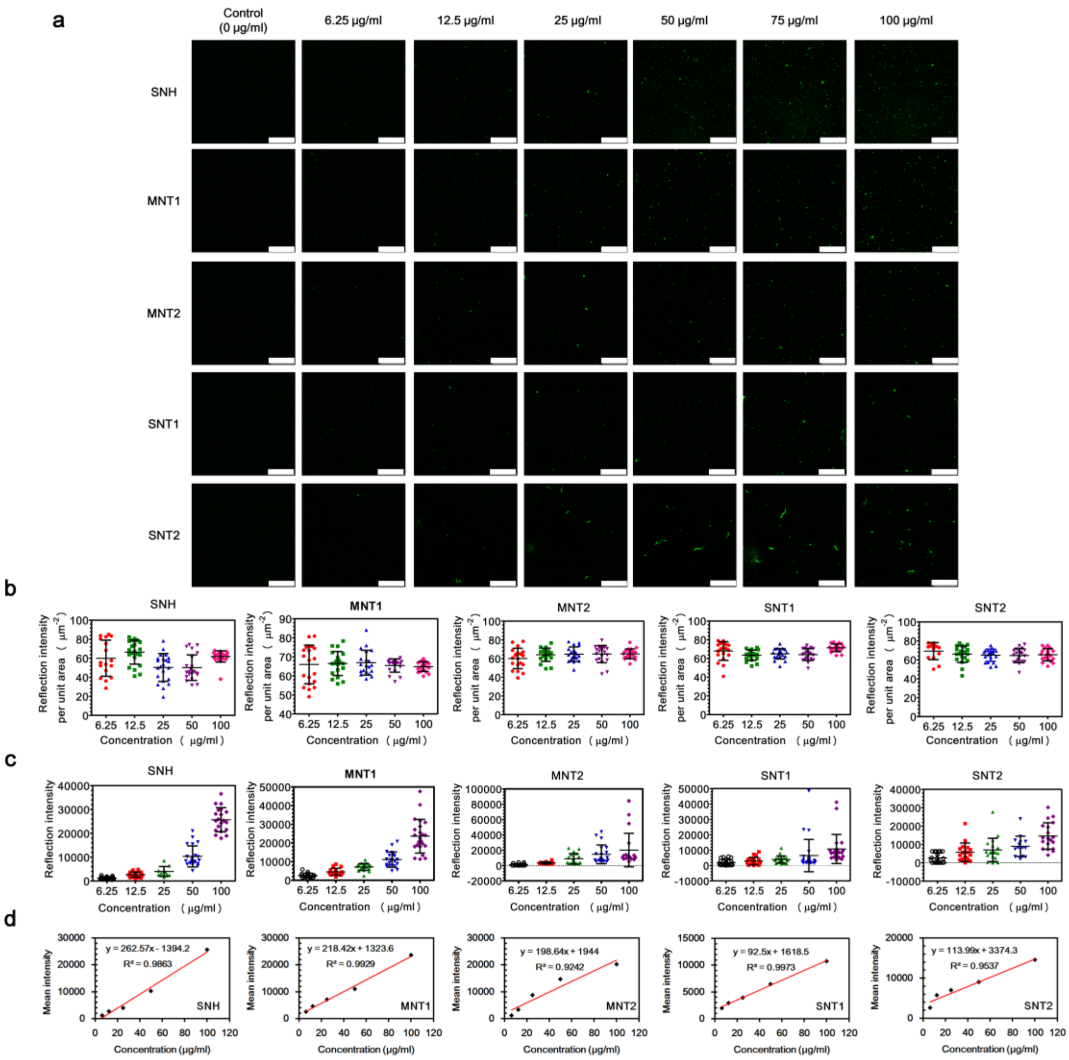

Supplementary Figure.18 The correlation analysis of LR intensity with the concentration of different nanocarbons in acellular model. (a) LR images of different nanocarbons with various concentrations immobilized in polyacrylamide gel. Scale bar: 25 μm. (b) The correlation diagrams of LR intensity per unit area of different nanocarbons with concentration. (c) The correlation diagrams of LR intensity of different nanocarbons with concentration. In (b) and (c), at least 25 independent fields (25 μm X 25 μm) in CLSM detection were randomly selected for LR intensity measurement. Data were presented as means ± s.d. (d) The linear correlations between the mean intensity of laser reflection and the incubation concentration of different nanocarbons via the linear regression method based on the mean value determination in (c).

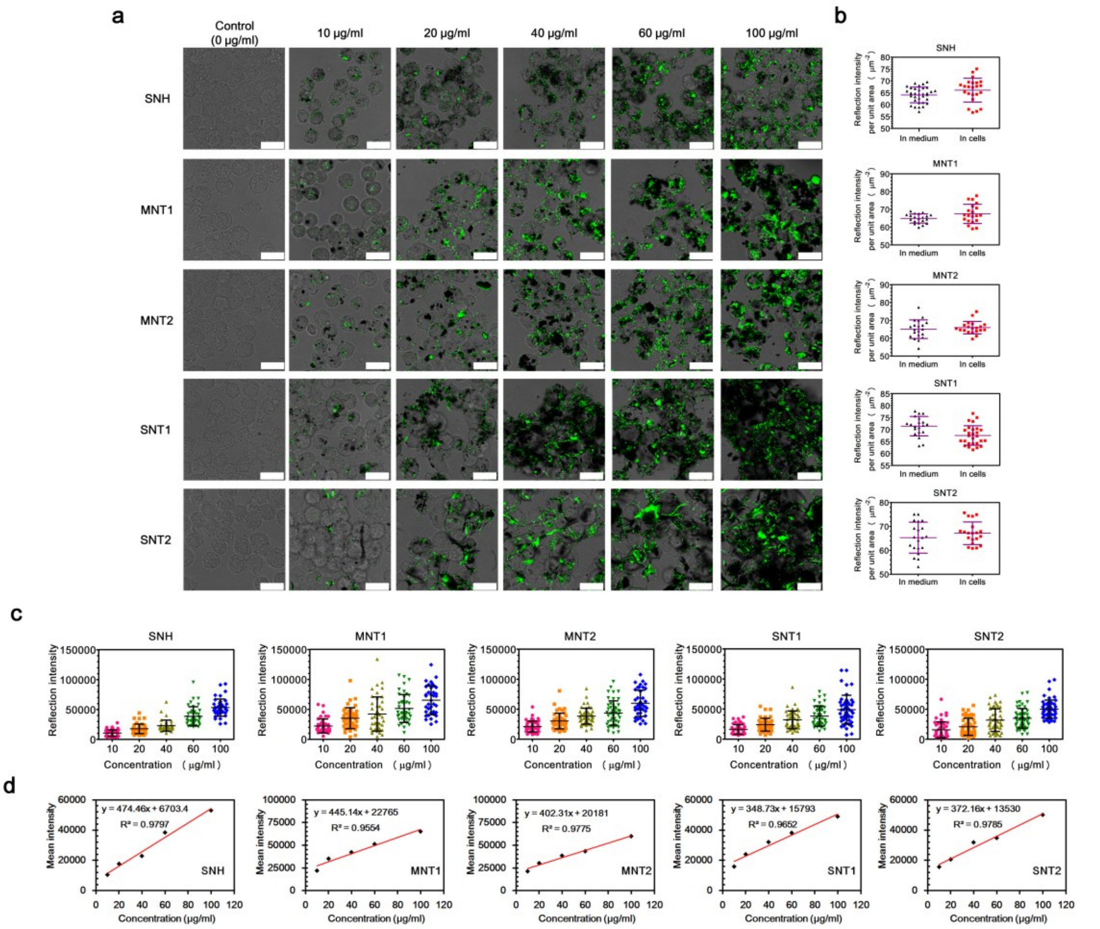

Supplementary Figure.19 The correlation analysis of LR intensity with the concentration of different nanocarbons in J774A.1 cell model. (a) LR images of J774A.1 cells detected by CLSM after the incubation of nanocarbons with different concentrations. Scale bar: 25 μm. Scale bar: 25 μm. (b) The comparison of LR intensity per unit area of nanocarbons between acellular and cellular models in the case of 100 μg ml^{-1} incubation. (c) The correlation diagrams of LR intensity of different nanocarbons with the incubation concentration. In (b) and (c), at least 25 independent fields (25 μm X 25 μm) in CLSM detection or over 100 cells were randomly selected for LR intensity measurement. Data were presented as means ± s.d. (d) The linear correlations between the mean intensity of laser reflection and the incubation concentration of different nanocarbons via the linear regression method based on the mean value determination in (c).

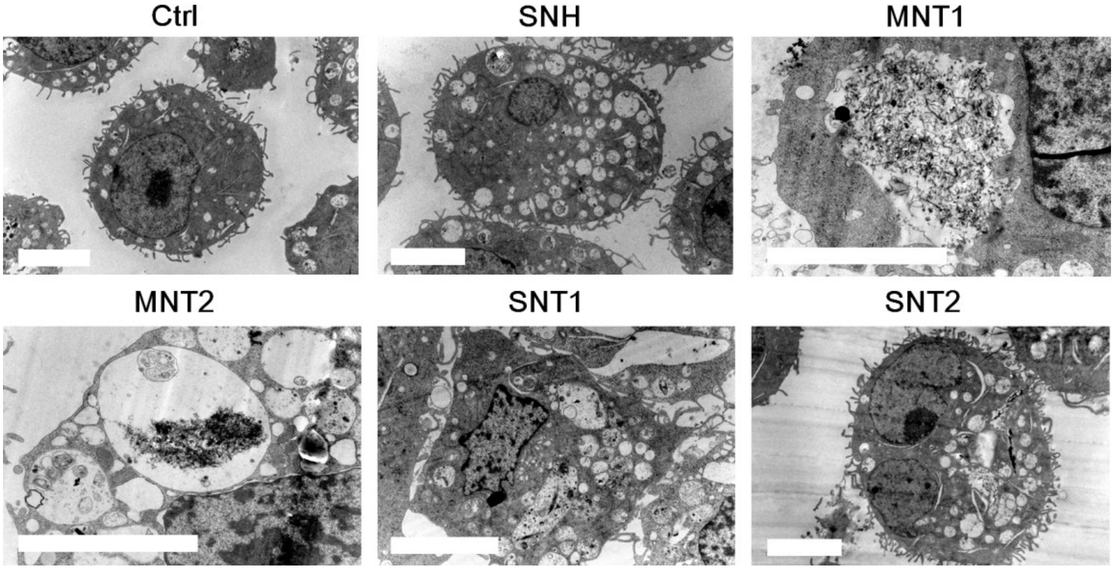

Supplementary Figure.20 TEM images of cells with low magnification after nanocarbon incubations. Scale bar: 5 μm. The internalizations of nanocarbons caused the obvious increases of intracellular vesicles and the deformation of cell membranes, indicating the phagocytosis mechanism for SNH and nanotubes.

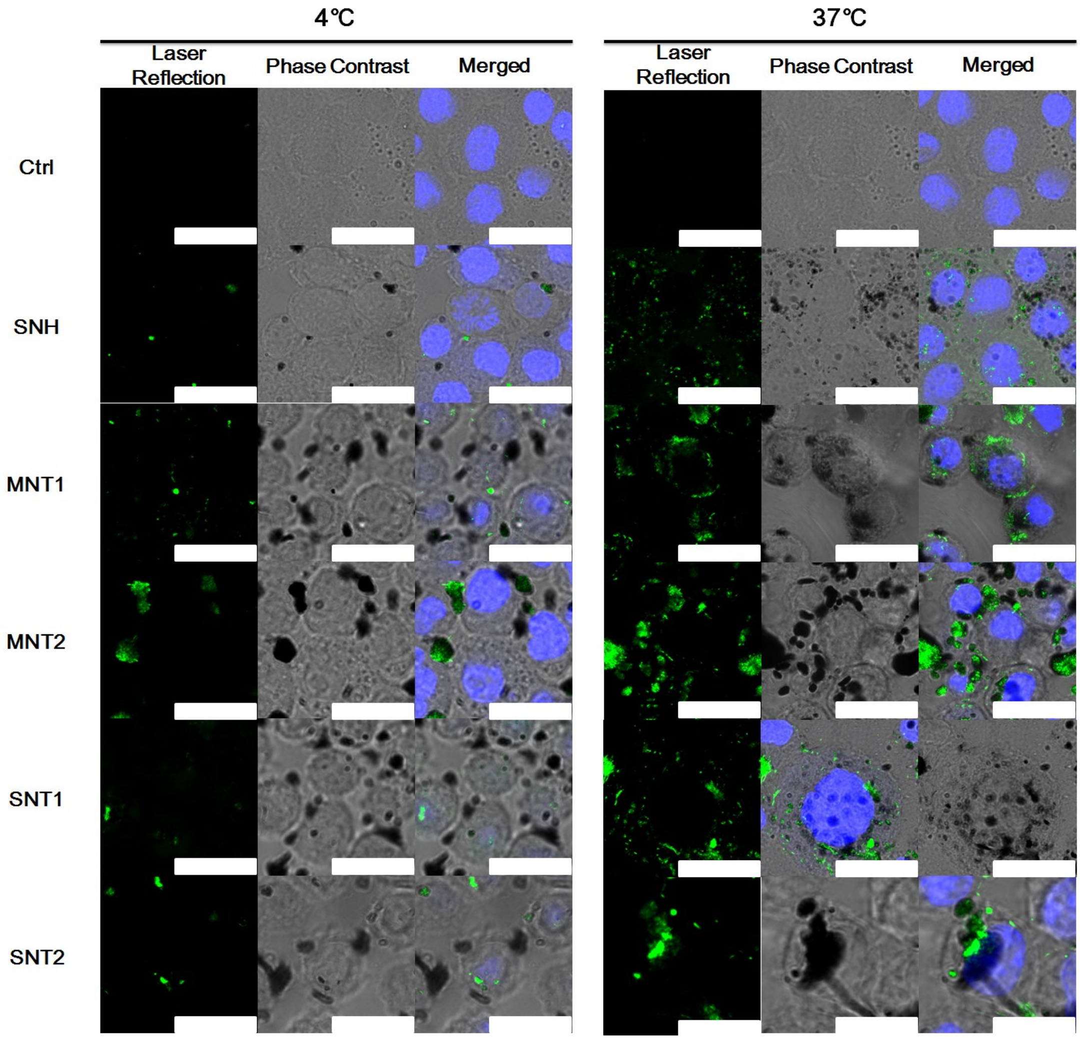

Supplementary Figure.21 Confocal images of cellular uptakes in different incubation temperatures. Scale bar: 25 μm. When nanocarbons were incubated with cells at 4 ℃ for 24 h, it was obviously to find the decreases of intracellular materials (LR signals, green pseudo color) compared to 37 ℃ incubation groups. Nuclei were stained with Hoechst 33258 in the investigation.

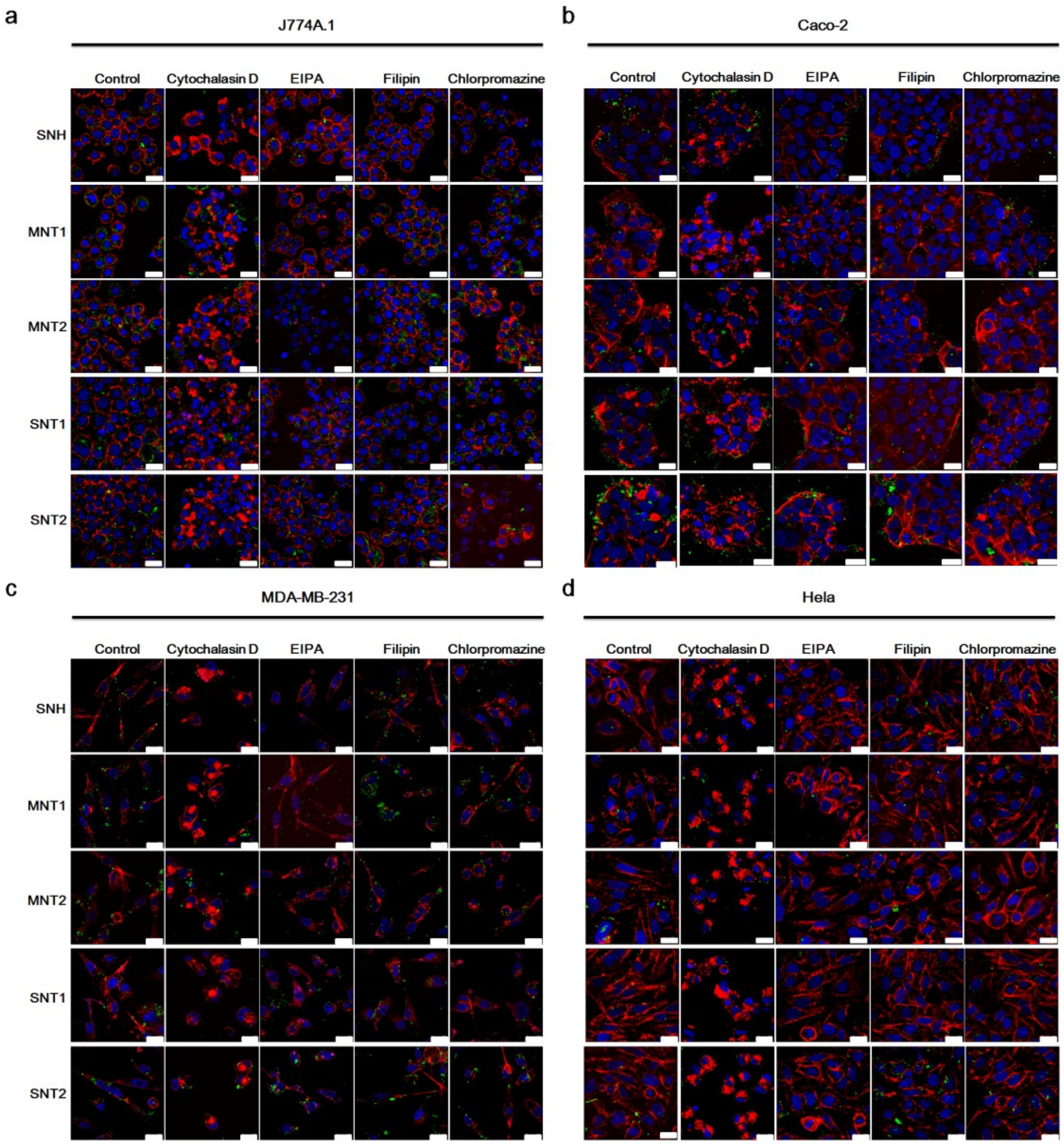

Supplementary Figure.22 The confocal images of Caco-2, MDA-MB-231, Hela and J774A.1 cell lines after treatment with different nanocarbons, with or without different endocytosis inhibitors. Cell nuclei were labeled with Hoechst 33258, and the microfilaments were stained with rhodamine-phalloidine (red pseudo color). Intracellular nanocarbons were detected via LR technology and shown with green pseudo color. Scale bar: 25 μm.

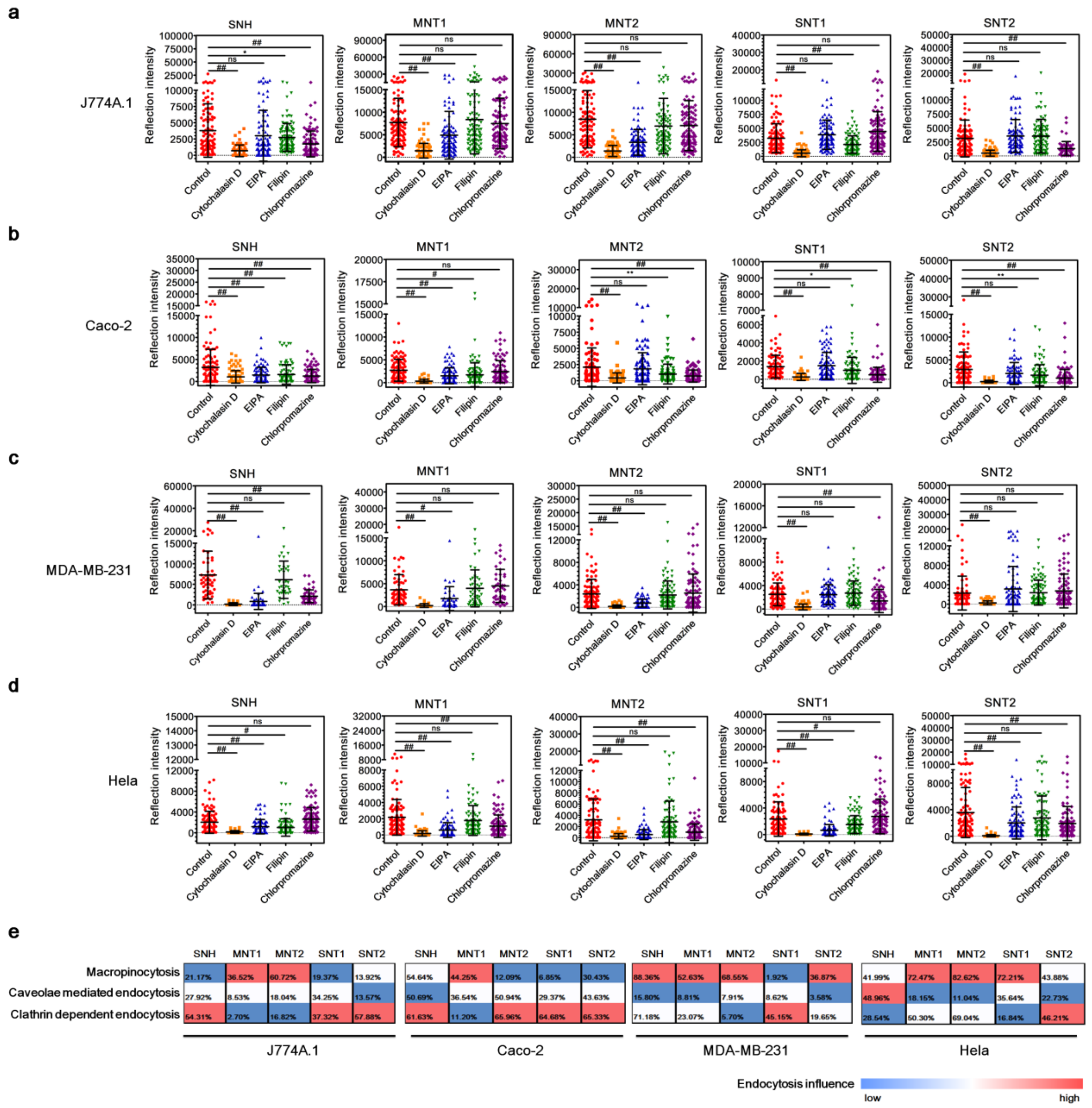

Supplementary Figure.23 The quantitative cell uptake analysis of different nanocarbons based on laser reflection imaging in (a) J774A.1, (b) Caco-2, (c) MDA-MB-231 and (d) Hela cell lines, with or without different endocytosis inhibitors. In (a) to (d), over 100 cells in CLSM detection were randomly selected for LR intensity measurement. Data were presented as means ± s.d. Statistical significances were compared with control group in each experiment and calculated by Student's t-test: * $p<0.05$, ** $p<0.01$, # $p<0.005$, ## $p<0.001$. (e) The heat maps based on the differences in pathway and mechanism of endocytosis between SNH and CNT in four types of cells. The percentages presented the inhibition ratios of different endocytosis inhibitors for the cellular uptakes of five nanocarbons compared to control group in four types of cells.

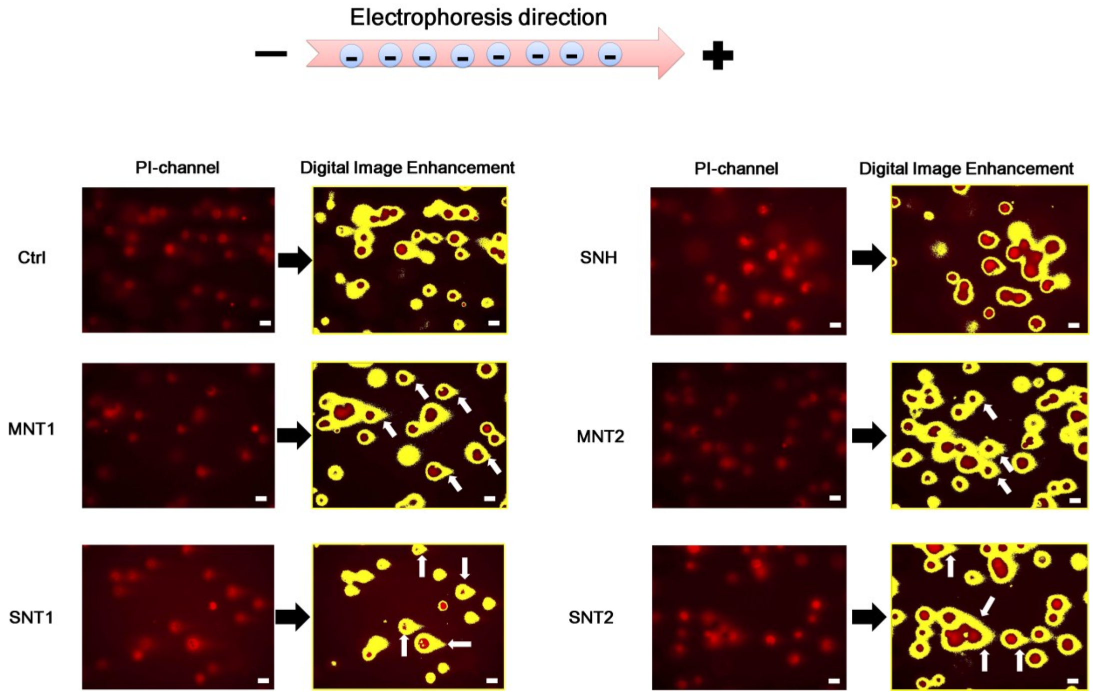

Supplementary Figure.24 Single cell gel-electrophoresis (comet assay) based on fluorescence microscopy. SNH incubation did not change the DNA distribution feature compared to Ctrl group. However, all nanotube treatments triggered the obvious comet characteristics (white arrows), which meant the productions of gene fragments. Scale bar: 10 μm.

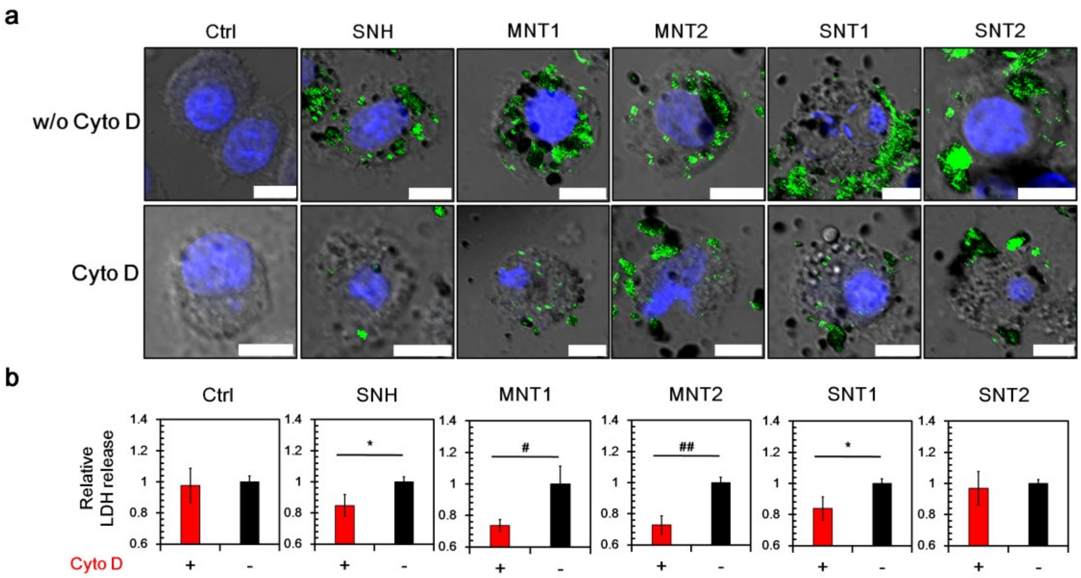

Supplementary Figure.25 (a) Confocal images of cells after nanocarbon incubations with and without Cyto D addition. Intracellular nanocarbons were detected by laser reflection (LR) technology and shown with pseudo green color. Scale bar: 10 μm. (b) Cytotoxic analysis of different nanocarbons based on LDH release assay with and without Cyto D addition (n=4). Data were presented as means ± s.d. Statistical significances were calculated by Student's t-test: * $p<0.05$, # $p<0.005$, ## $p<0.001$.

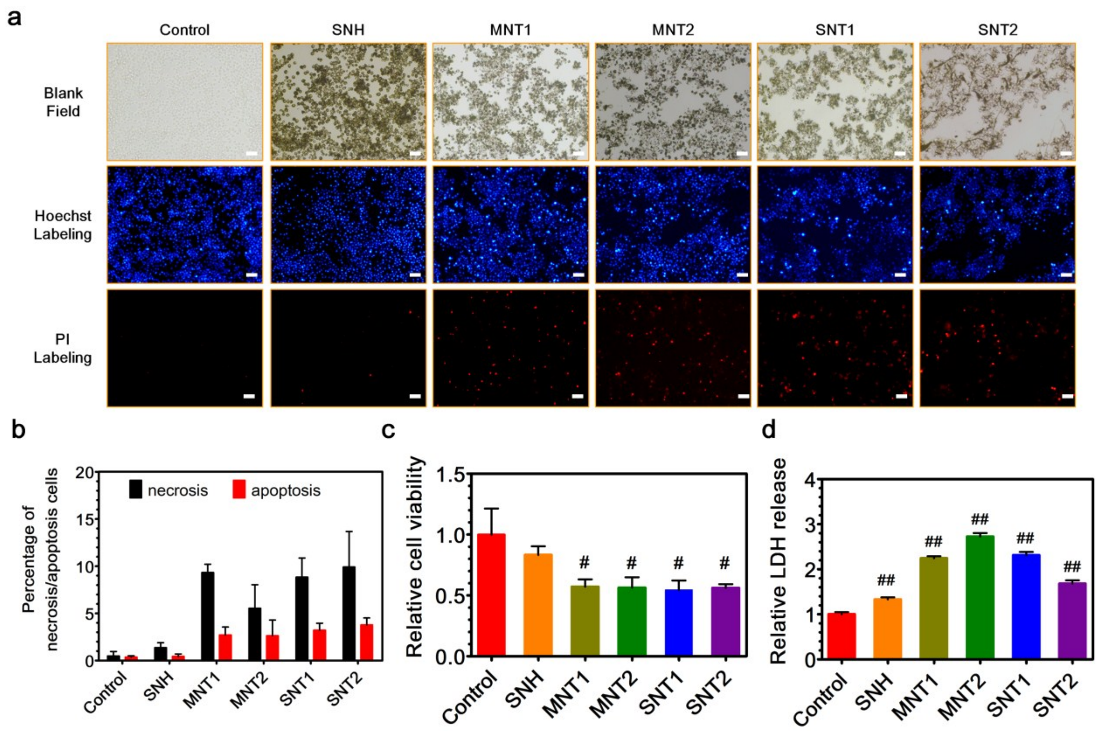

Supplementary Figure.26 The evaluations of cytotoxicity and cell death mechanism induced by different nanocarbons on the basis of normalization of cell uptake level. (a) Microscopy images of dead cells labeled by propidium iodide (PI) after treatment of different nanocarbons. Cells were also detected via microscopy in blank field model and labeled with Hoechst 33258 as reference. Scale bar: 50 μm. (b) The proportional comparison between necrosis and apoptosis after the cellular incubation with different nanocarbons via the apoptosis/necrosis assay detection (n=4). (c) The relative cell viability after treatment of nanocarbons detected by the MTT assay (n=3). (d) The relative LDH release after treatment of nanocarbons detected by LDH assay (n=3). In (c) and (d), data were compared with control group in each experiment and presented as means ± s.d. Statistical significances were calculated by Student's t-test, # $p<0.005$, ## $p<0.001$.

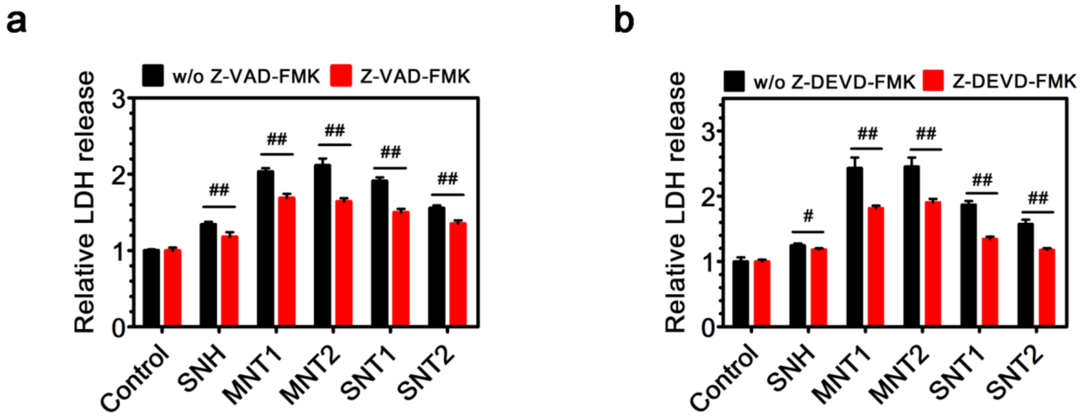

Supplementary Figure.27 The mechanism analysis on nanocarbon-induced cell death through apoptosis related pharmacological inhibition strategy detected by LDH release assay on the basis of normalization of uptake level of nanocabons. (a) Z-VAD-FMK as the pan-caspase inhibitor and (b) Z-DEVD-FMK as the specific caspase-3 inhibitor were selected to evaluate the death mechanism caused by nanocarbons. Data were compared with no inhibitor adding groups for each type of nanocarbons and presented as means ± s.d (n=3). Statistical significances were calculated by Student's t-test: # $p<0.005$; ## $p<0.001$.

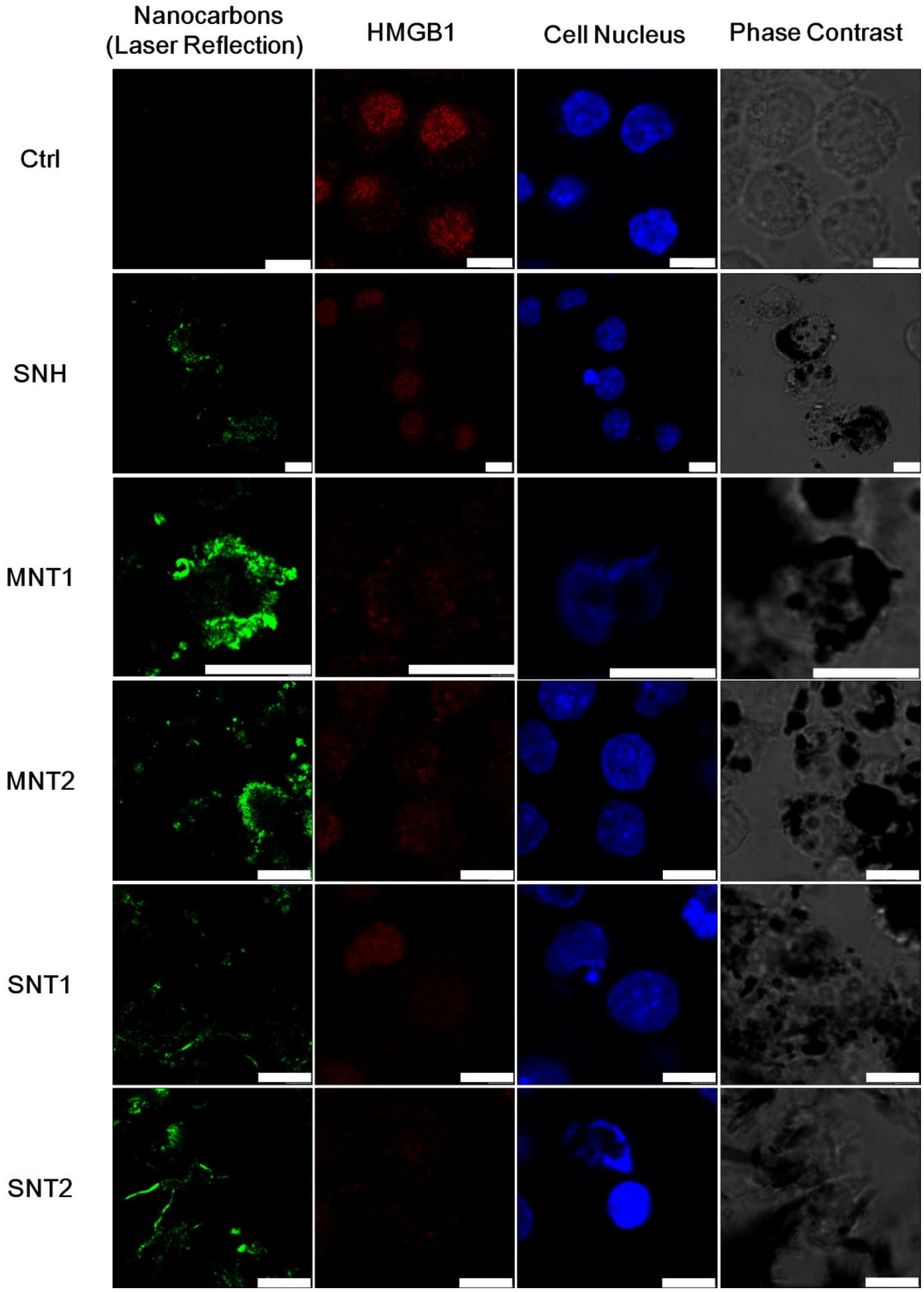

Supplementary Figure.28 Confocal images of intracellular HMGB1 after nanocarbon incubations. Scale bar: 10 μm. Notably, the fluorescence intensities of HMGB1 in cell nuclei were apparently decreased. Nuclei were also stained with Hoechst 33258 as reference in the investigation.

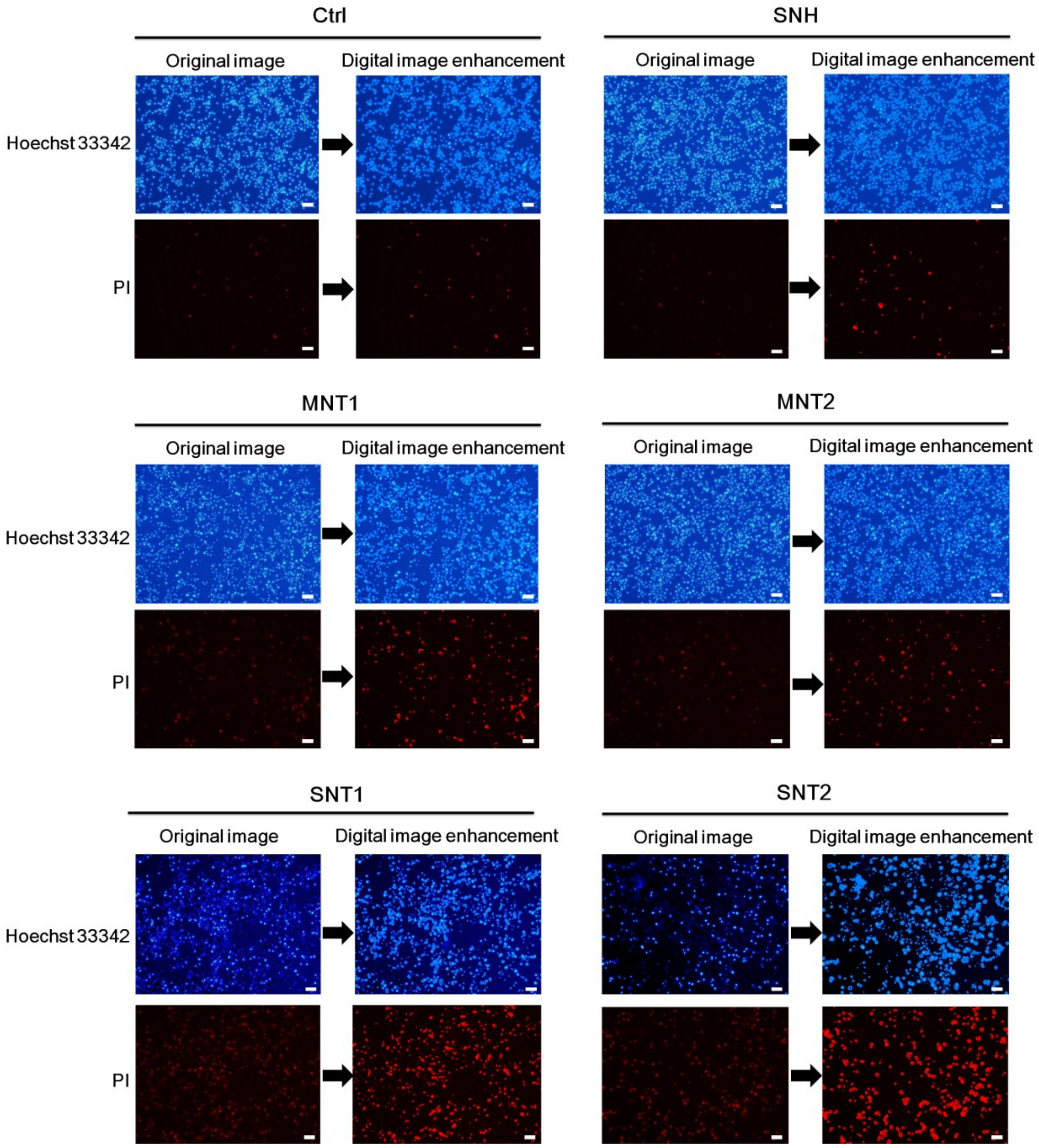

Supplementary Figure.29 Fluorescence micrographs of cells after nanocarbon incubations detected by apoptosis/necrosis assay kit. Cells were stained with Hoechst 33342 and PI respectively. The obtained graphs were digital enhanced by IPP software to highlight the necrosis and apoptosis cells. Scale bar: 50 μm.

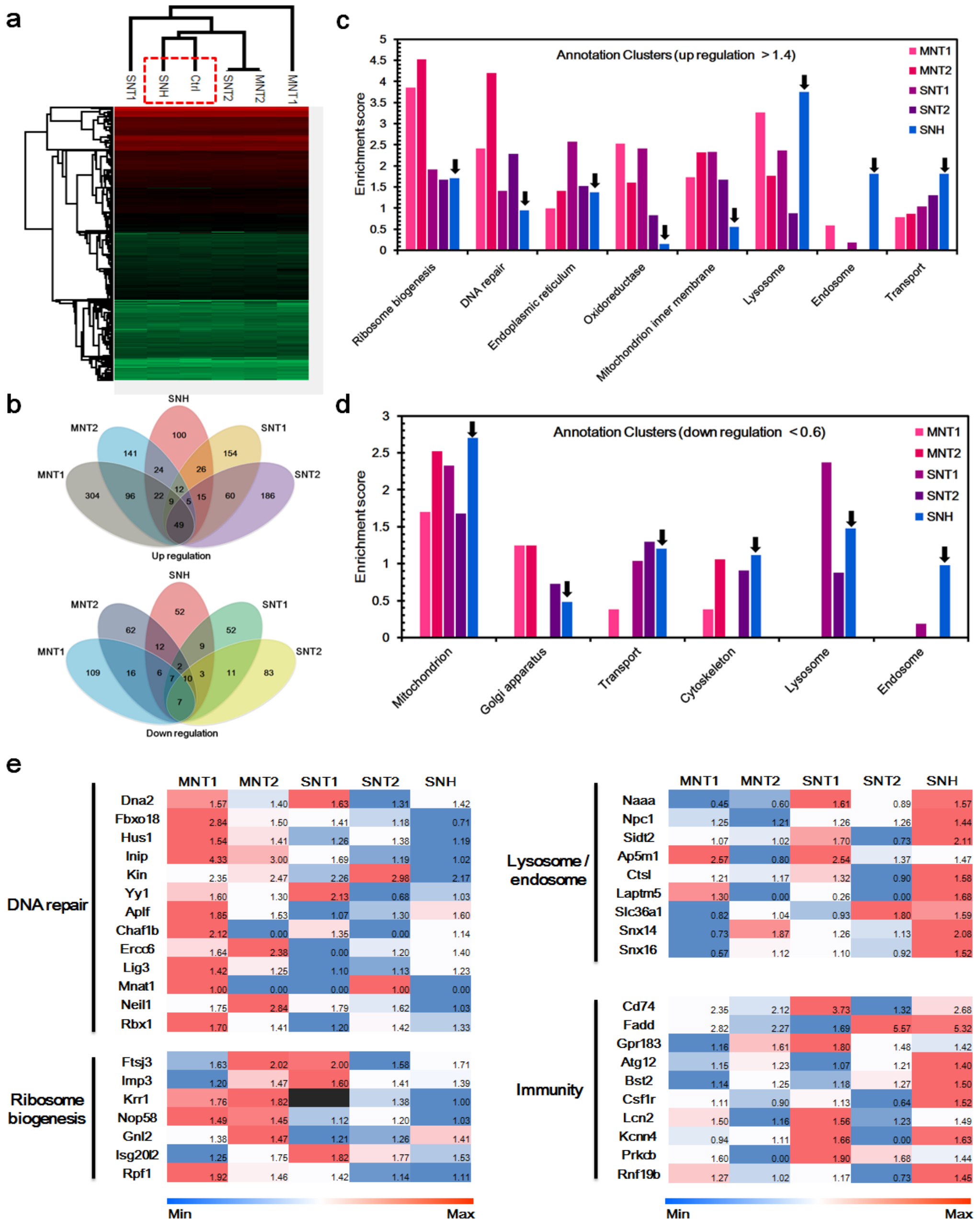

Supplementary Figure.30 The total protein proteomic analysis on the basis of normalization of cell uptakes for different nanocarbons. (a) Hierarchical clustering analysis on the changes of cellular proteins investigated by LFQ proteomics after incubation of nanocarbons with cells based on the uptake normalization (b) The Venn diagrams of cellular proteins with more than 40% expression change over control group after treatment of nanocarbons. (c,d) The gene functional annotation clustering of (c) up-regulated and (d) down-regulated proteins based on enrichment analysis after the incubation of different nanocarbons (e) The heat maps on the expression comparison of changed proteins that involved in part of key cellular processes after the incubation of nanocarbons. The values in heat maps represented the expression ratios of identified

proteins compared to control group.

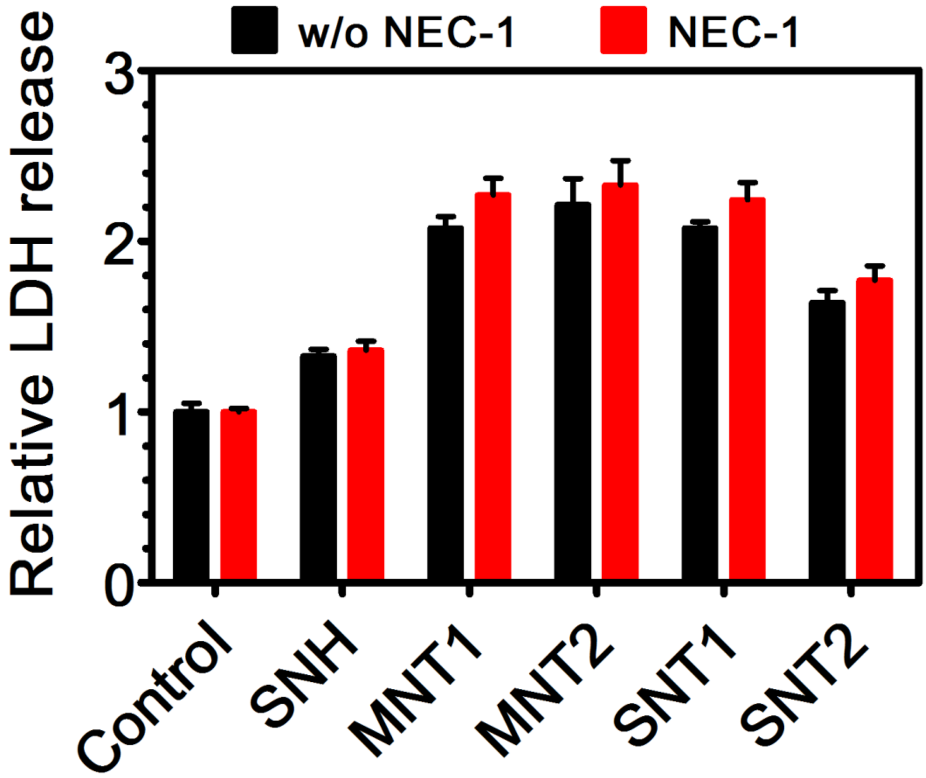

Supplementary Figure.31 Cytotoxicity detections of different nanocarbons with and without NEC-1incubation on the basis of normalization of uptake level of nanocabons. Data were compared with no inhibitor adding groups for each type of nanocarbons and presented as means ± s.d (n=3). Statistical significances were calculated by Student's t-test.

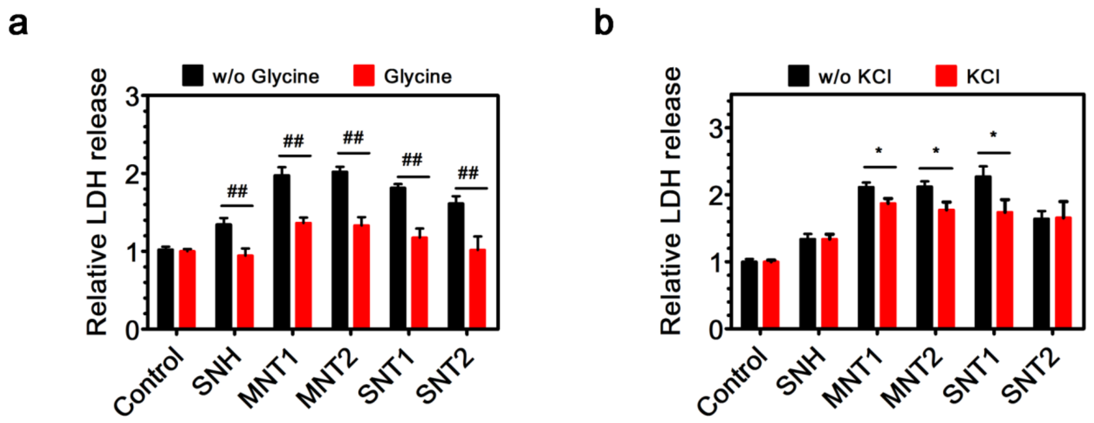

Supplementary Figure.32 Cytotoxicity detections of different nanocarbons with and without (a) glycine and (b) KCl as the inhibitors of pyroptosis on the basis of normalization of uptake level of nanocabons. Data were compared with no inhibitor adding groups for each type of nanocarbons and presented as means ± s.d (n=3). Statistical significances were calculated by Student's t-test: * $p<0.05$, ## $p<0.001$.

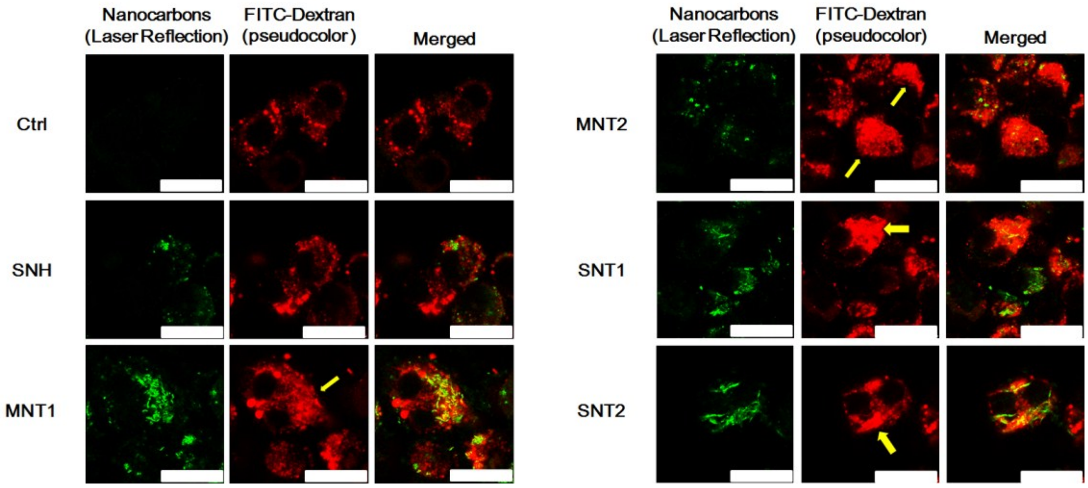

Supplementary Figure.33 Confocal images of intracellular lysosomes labeled with FITC-Dextran (FD) after nanocarbon incubations. Compared to the spot-like distribution of lysosomes (red pseudo color) for Ctrl and SNH group, CNT incubations caused the obvious leakages and cytoplasmic diffusion of FD (yellow arrows). Scale bar: 25 μm.

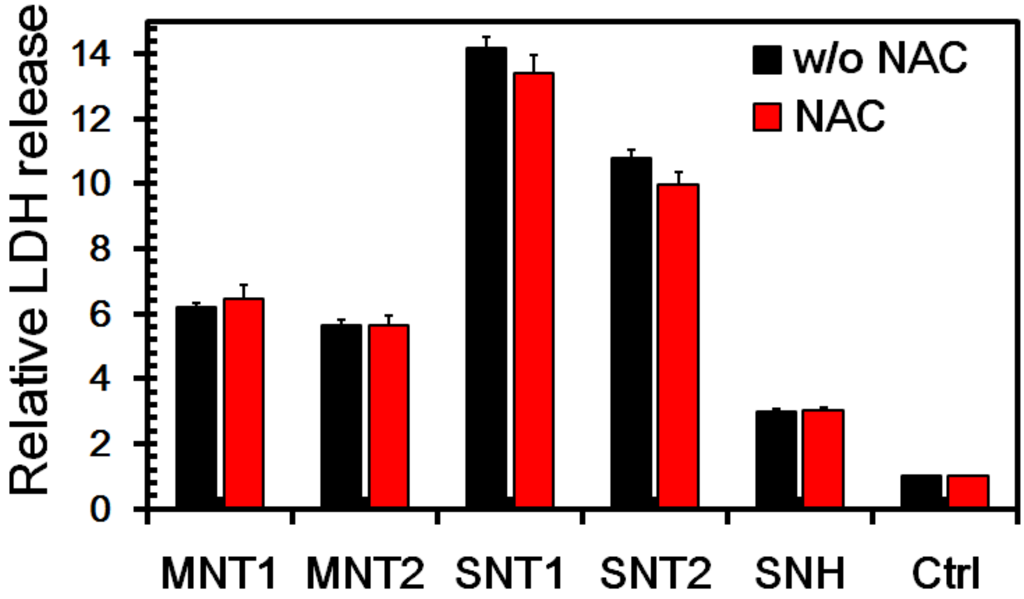

Supplementary Figure.34 Cytotoxicity evaluation of different nanocarbons after the treatment of specific ROS inhibitor NAC. Data were compared with no inhibitor adding groups for each type of nanocarbons and expressed as mean ± s.d. (n=3).

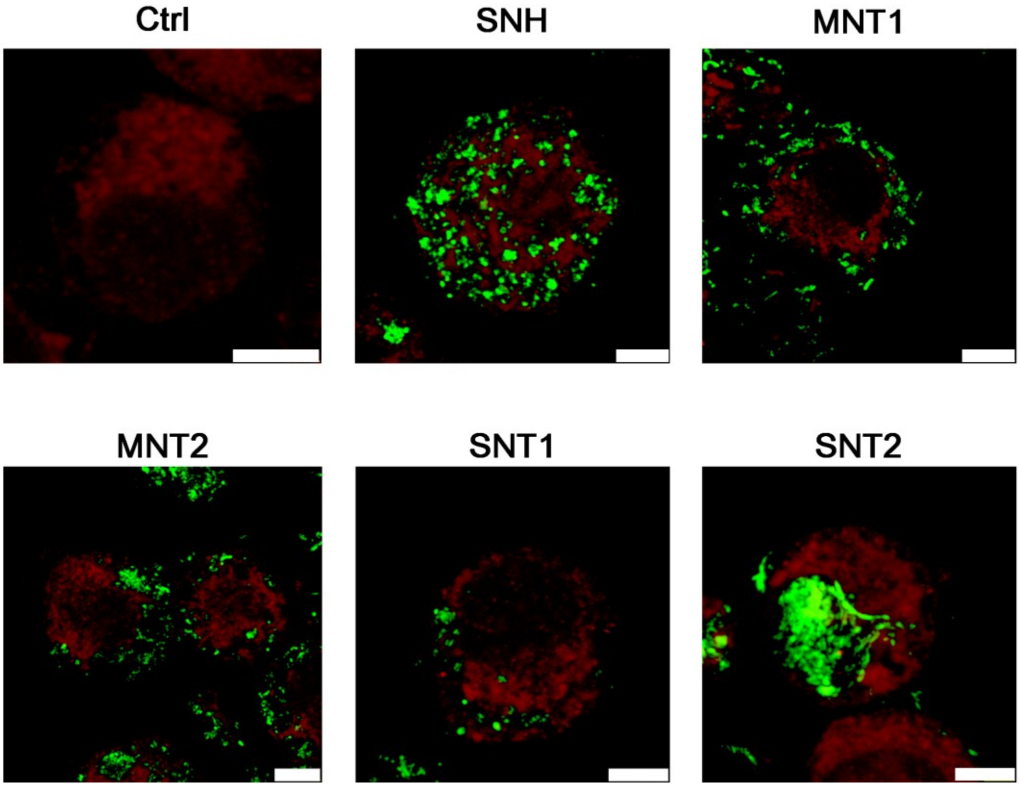

Supplementary Figure.35 Confocal images of autophagy after nanocarbon incubations. LC-3B (red color) as the marker of autophagosomes was labeled with specific antibody. Intracellular nanocarbons (green color) were detected by LR technology. No obvious co-localization feature was observed for five nanocarbon groups. Scale bar: 5 μm.

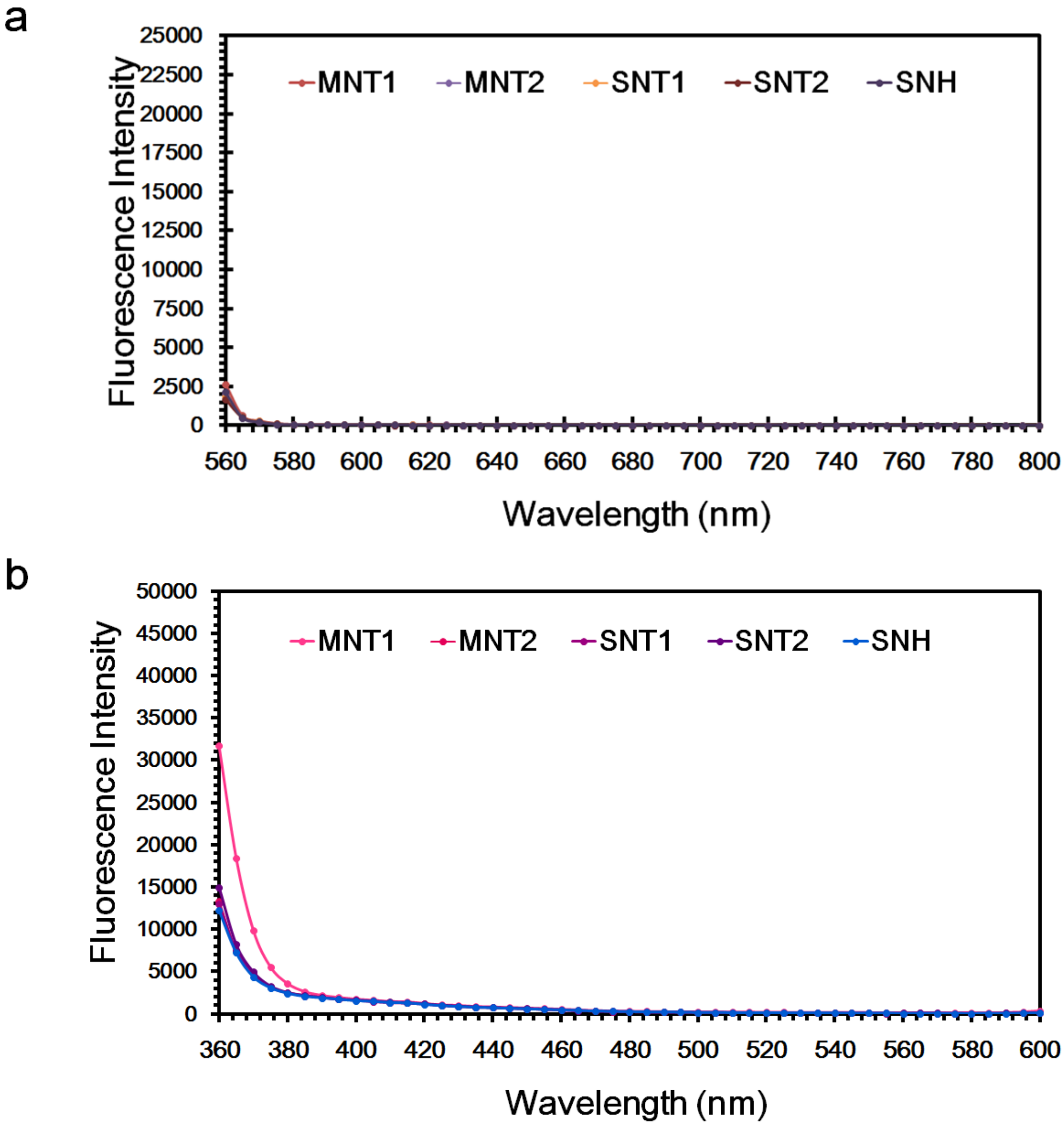

Supplementary Figure.36 Fluorescence spectrums of different nanocarbons under nile red (a, EX 552nm) and pryene (b, EX 334nm) detection conditions.

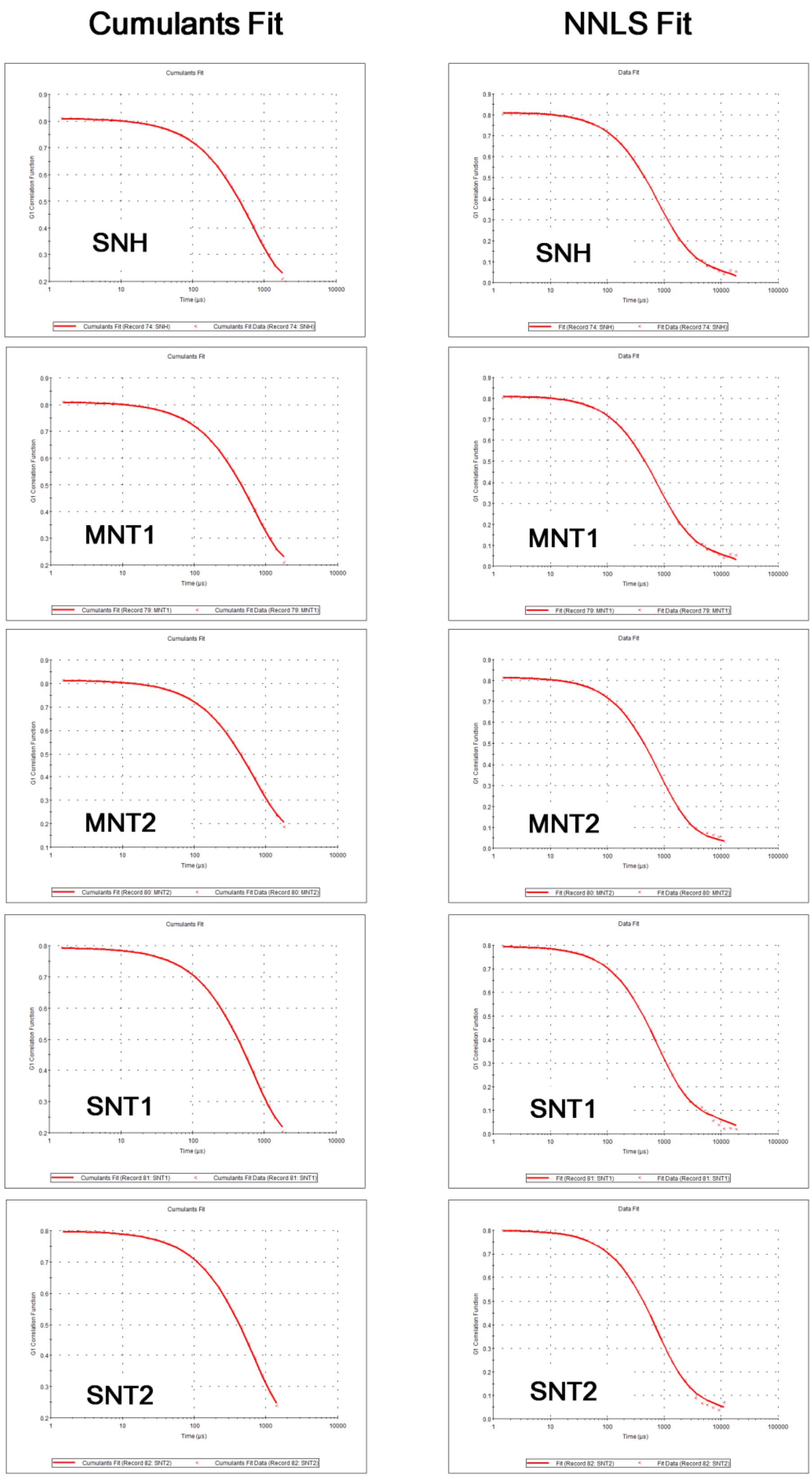

Supplementary Figure.37 Autocorrelation fitting of light scattering intensities of nanocarbons in aqueous medium detected by DLS. Two different deconvolution algorithms, including cumulants analysis and non-negatively constrained least squares (NNLS) fitting algorithm, were performed in study to calculate the hydrodynamic diameters.

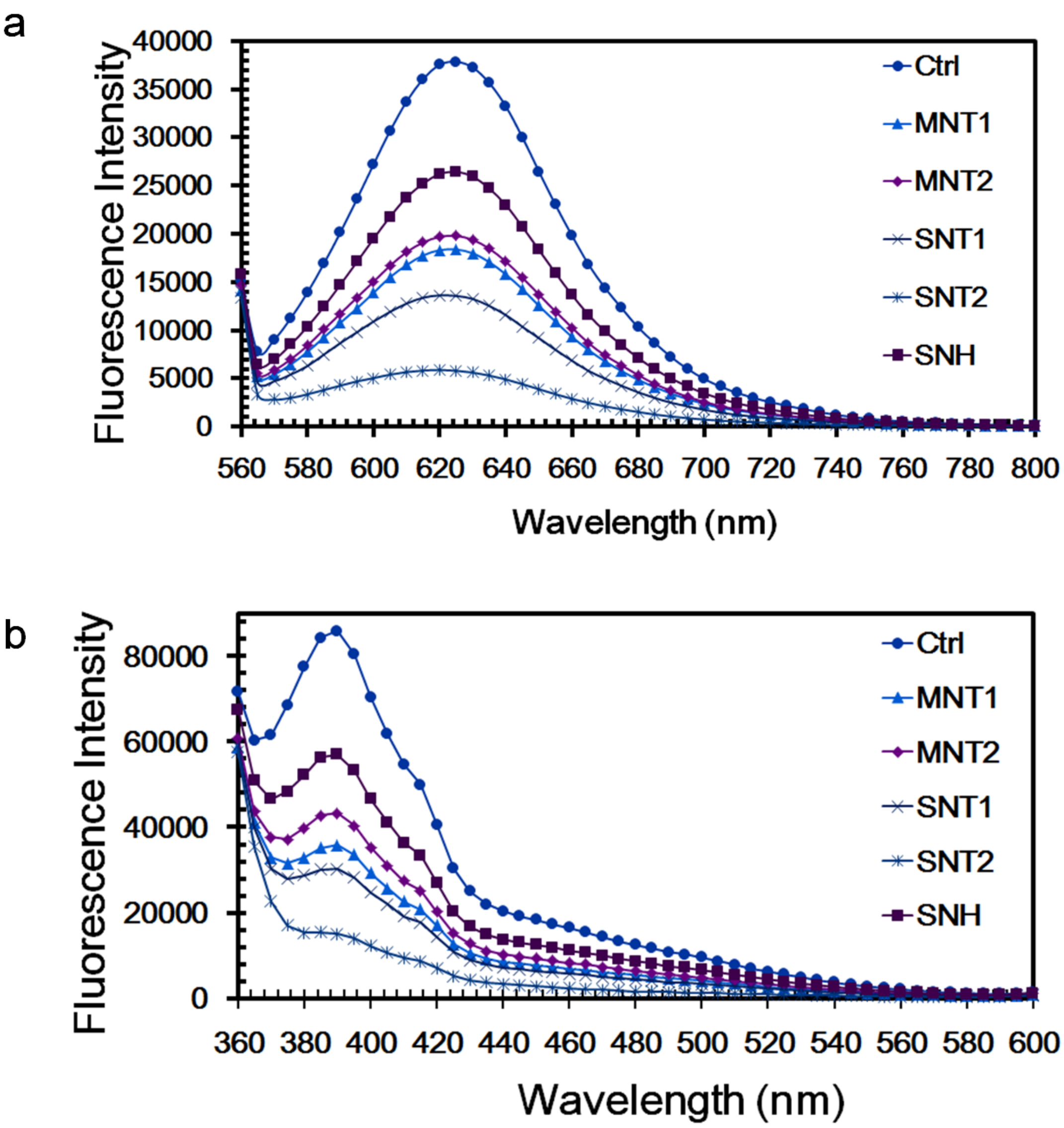

Supplementary Figure.38 Fluorescence spectrums of ALMs labeled with nile red (a) and pyrene (b) respectively after nanocarbon incubations. The concentations of different nanocarbons were adjusted based on NNLS algorithm data conversion to assure different nanocarbons possessed the identical apparent contact areas with ALMs during the investigation.

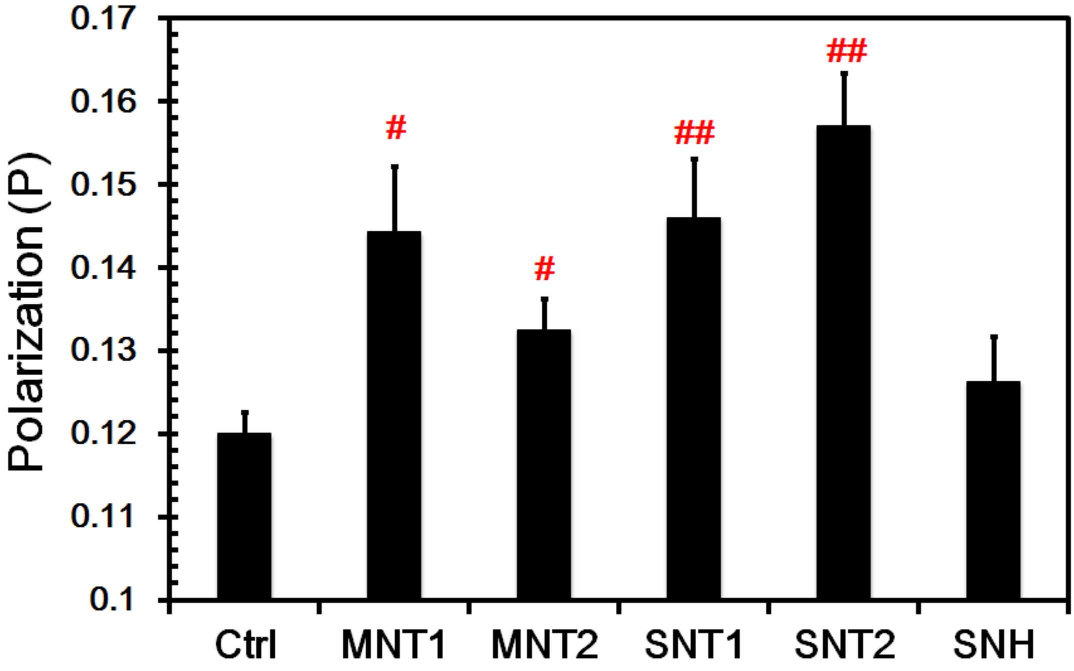

Supplementary Figure.39 Polarization (P) measurements of ALMs labeled with DPH after the incubations of nanocarbons, exhibiting the obvious changes of membrane fluidity caused by nanotubes. The concentations of different nanocarbons were adjusted based on NNLS algorithm data conversion to assure different nanocarbons possessed the identical apparent contact areas with ALMs during the investigation. Data are expressed as mean ± s.d. (n=3) and compared with Ctrl. #p<0.005; ##p<0.001.

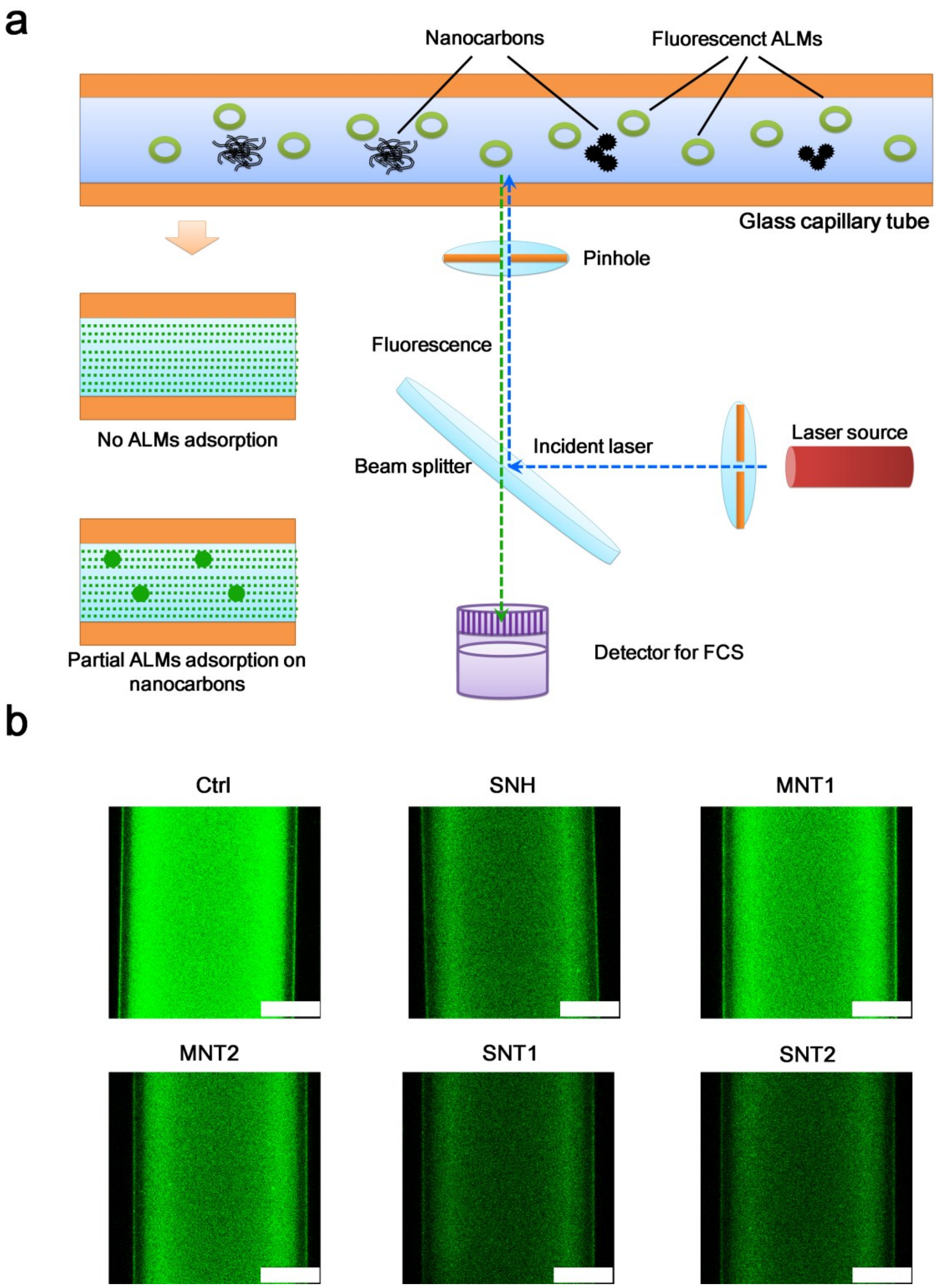

Supplementary Figure.40 Schematic diagrams of fluorescence correlation spectroscopy (FCS). (a) Monodispersed fluorescent ALMs were incubated with different nanocarbons in capillary. If ALMs had affinity with nanocarbons, they would bind on the material surface and assemble in aggregative forms, which could be detected by FCS based on the difference of diffusions. (b) Confocal images of the mixed dispersions that contained fluorescent ALMs and nanocarbons in the FCS detection. Scale bar: 100 μm.

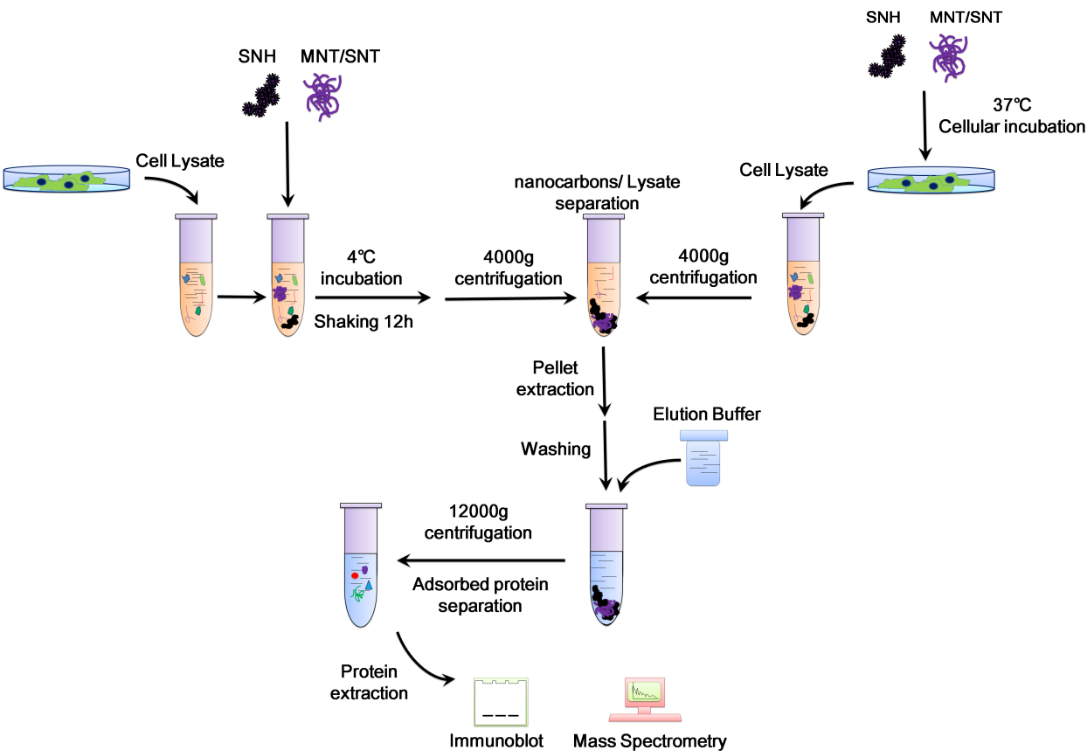

Supplementary Figure.41 Schematic of nanocarbon-protein interaction analysis. The flow chart show the separation and extraction process of high affinitive proteins with nanocarbons.

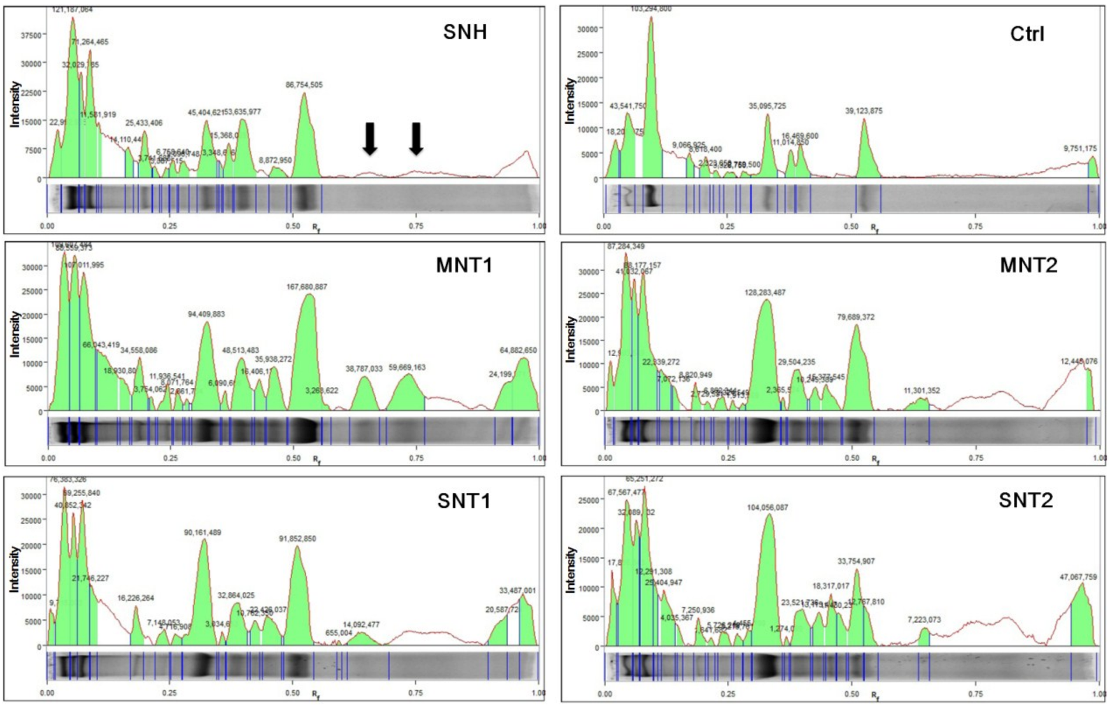

Supplementary Figure.42 Molecular weight distributions of affinitive protein with nanocarbons detected by SDS-PAGE. The intensity histograms based on gel strips were obtained via Image Lab software. Black arrows indicated that some stripes of bonded proteins in SNH group were different from CNT groups in distributions and intensities.

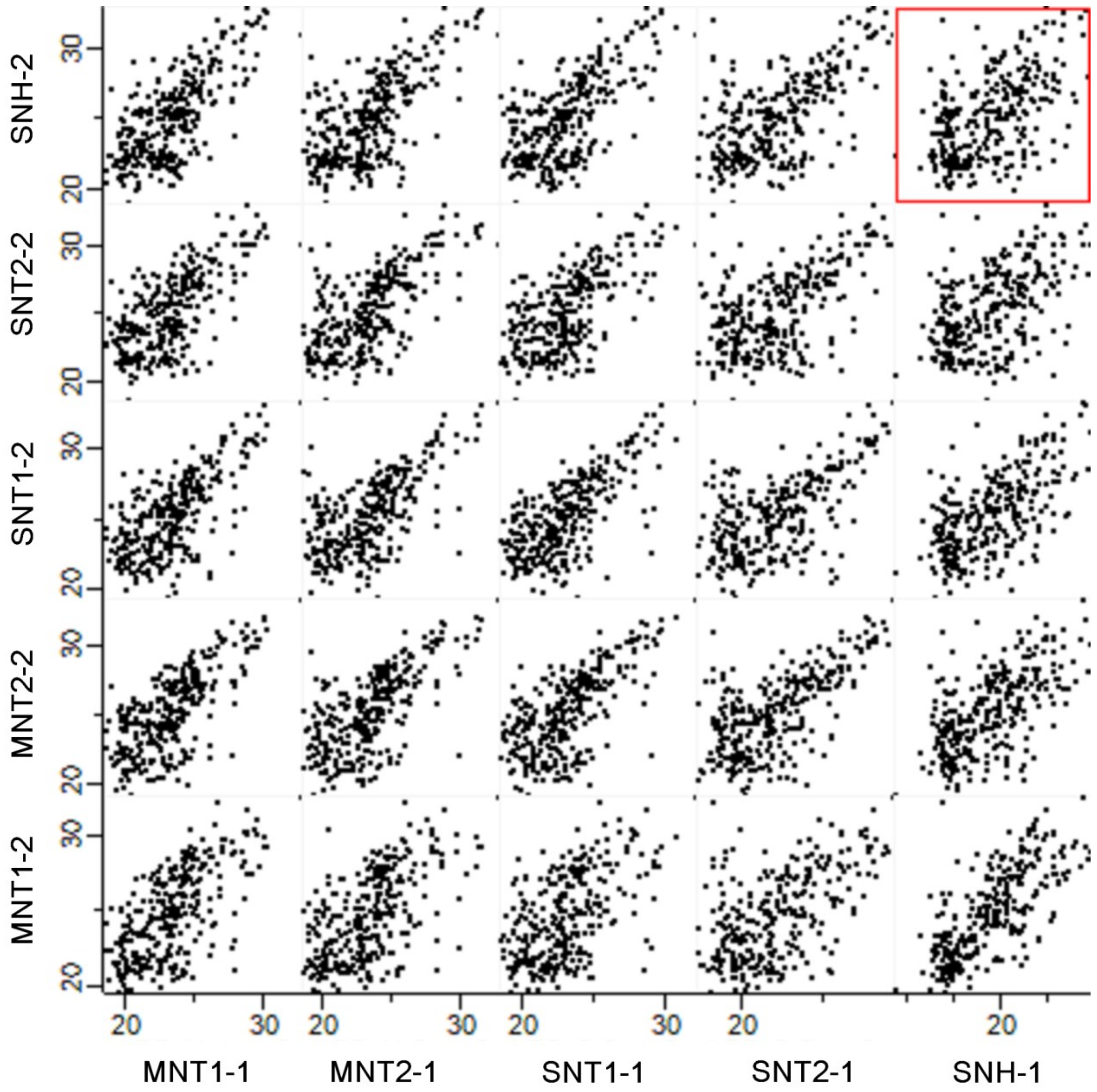

Supplementary Figure.43 The correlation analysis of two biological duplicative mass spectrometry (MS) experiments based on label-free quantification (LFQ) proteomics technology.

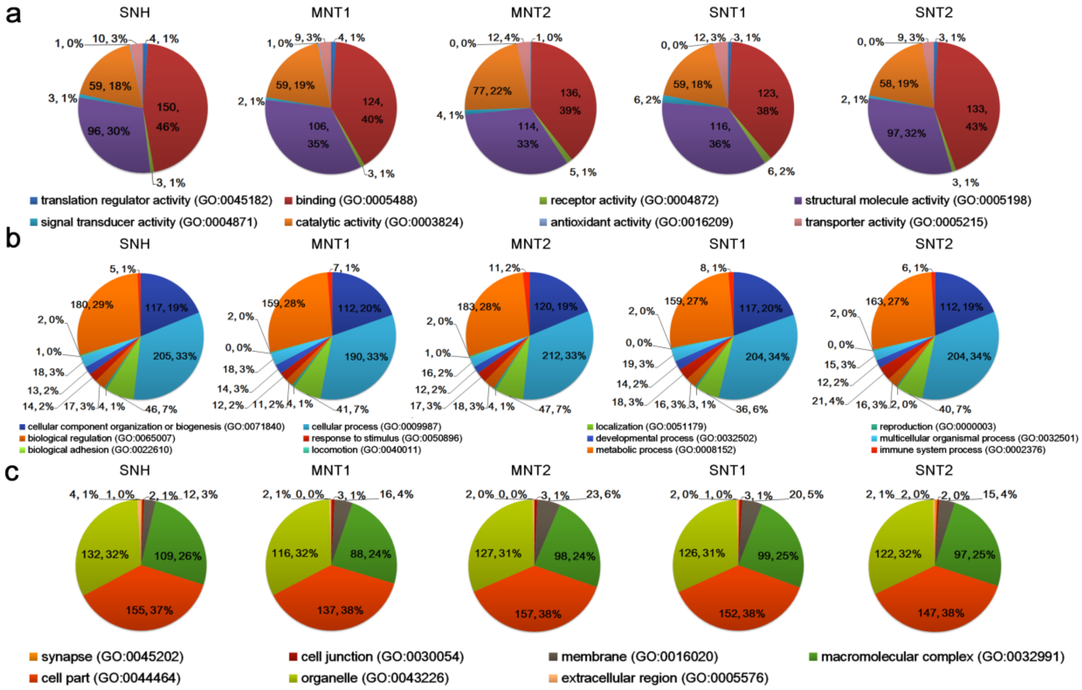

Supplementary Figure.44 Gene ontology (GO) analysis of affinitive proteins for different nanocarbon groups. The pie charts showed similar and distinct proportions of proteins based on (a) molecular function, (b) biological process and (c) cellular component classifications.

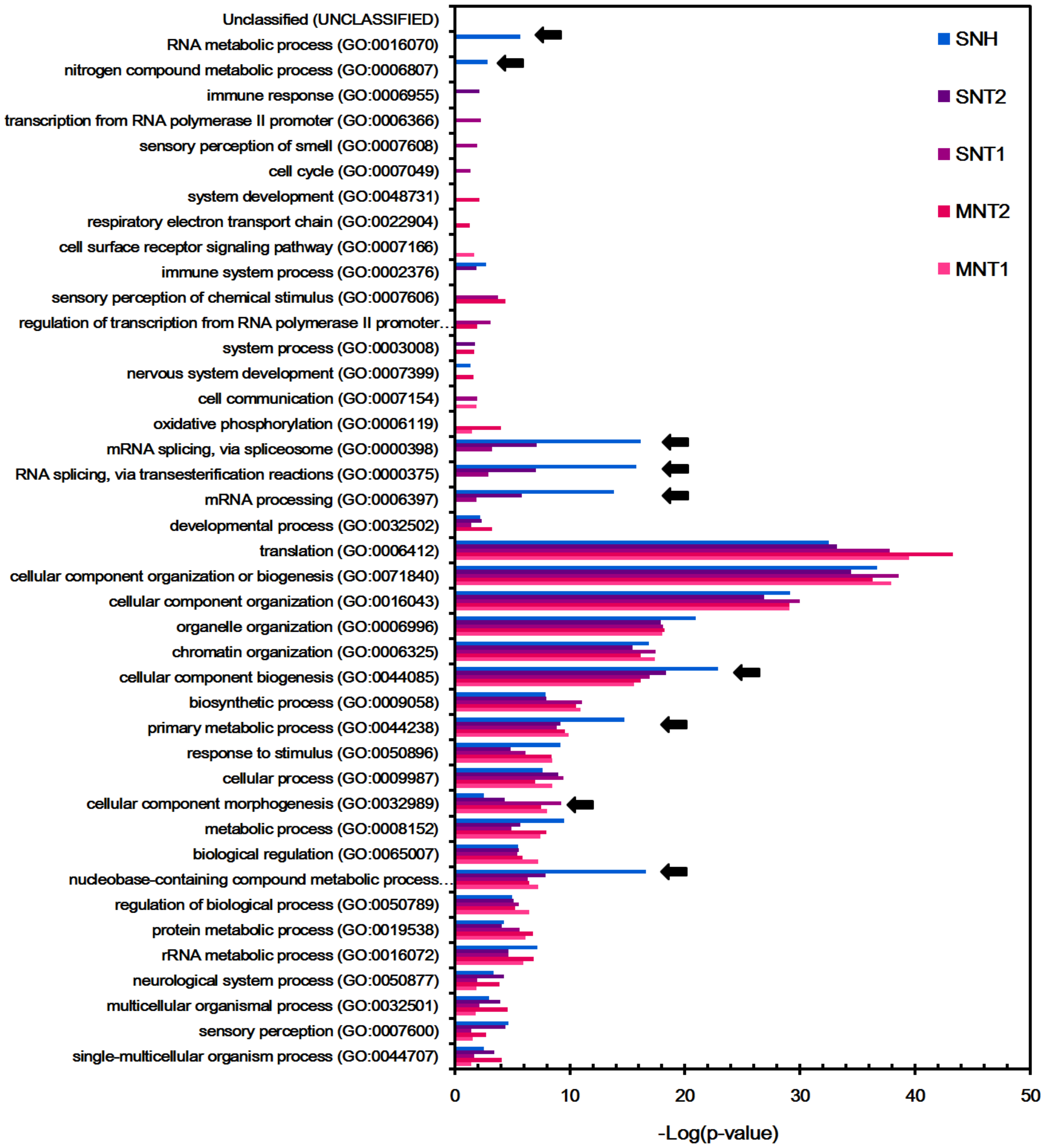

Supplementary Figure.45 Overrepresentation (OR) test of the affinitive proteins based on biological process (BP) classification. Black arrows showed the obvious distinctions in protein functions for SNH group compared to nanotubes.

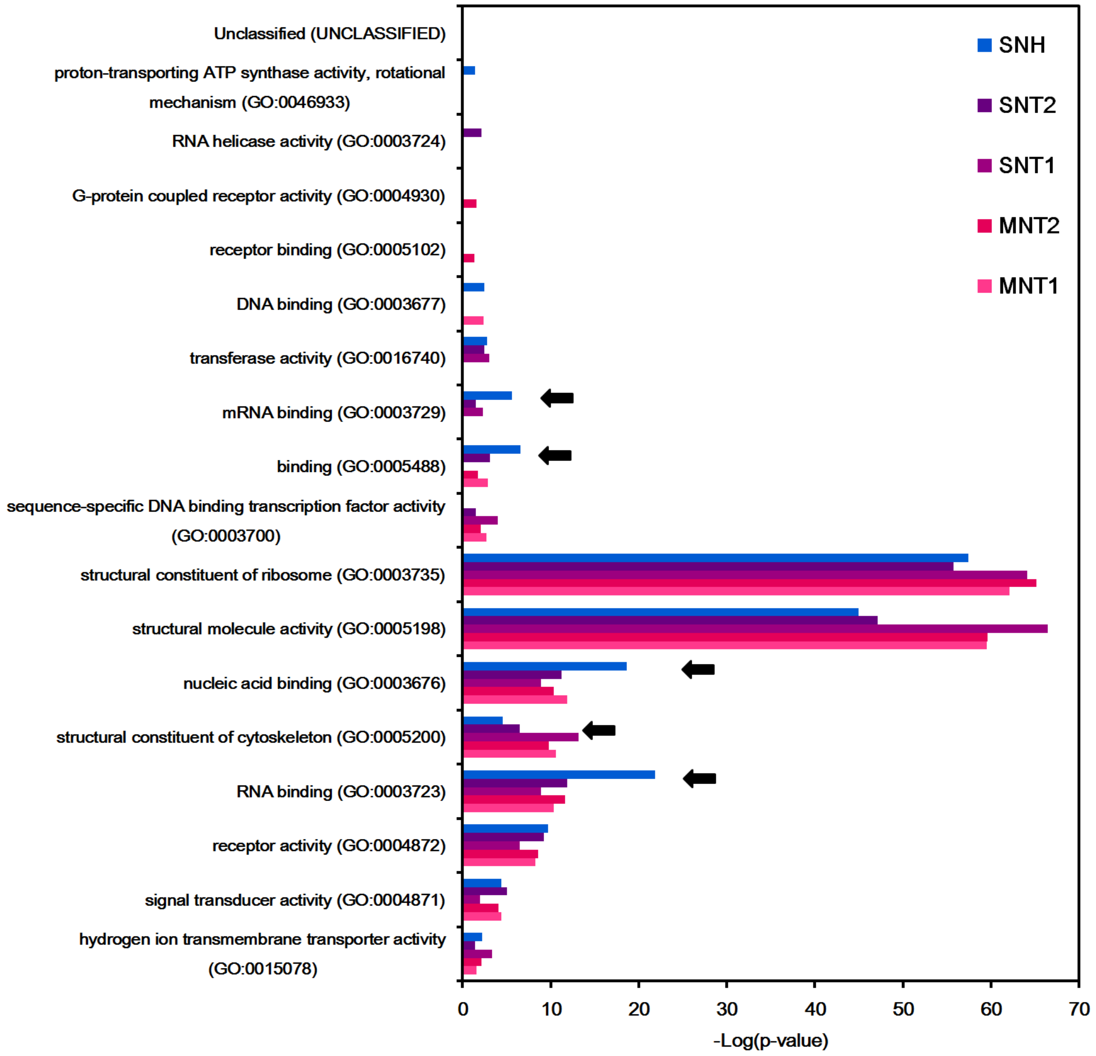

Supplementary Figure.46 Overrepresentation (OR) test of the affinitive proteins based on molecular function (MF) classification. Black arrows showed the obvious distinctions in protein functions for SNH group compared to nanotubes.

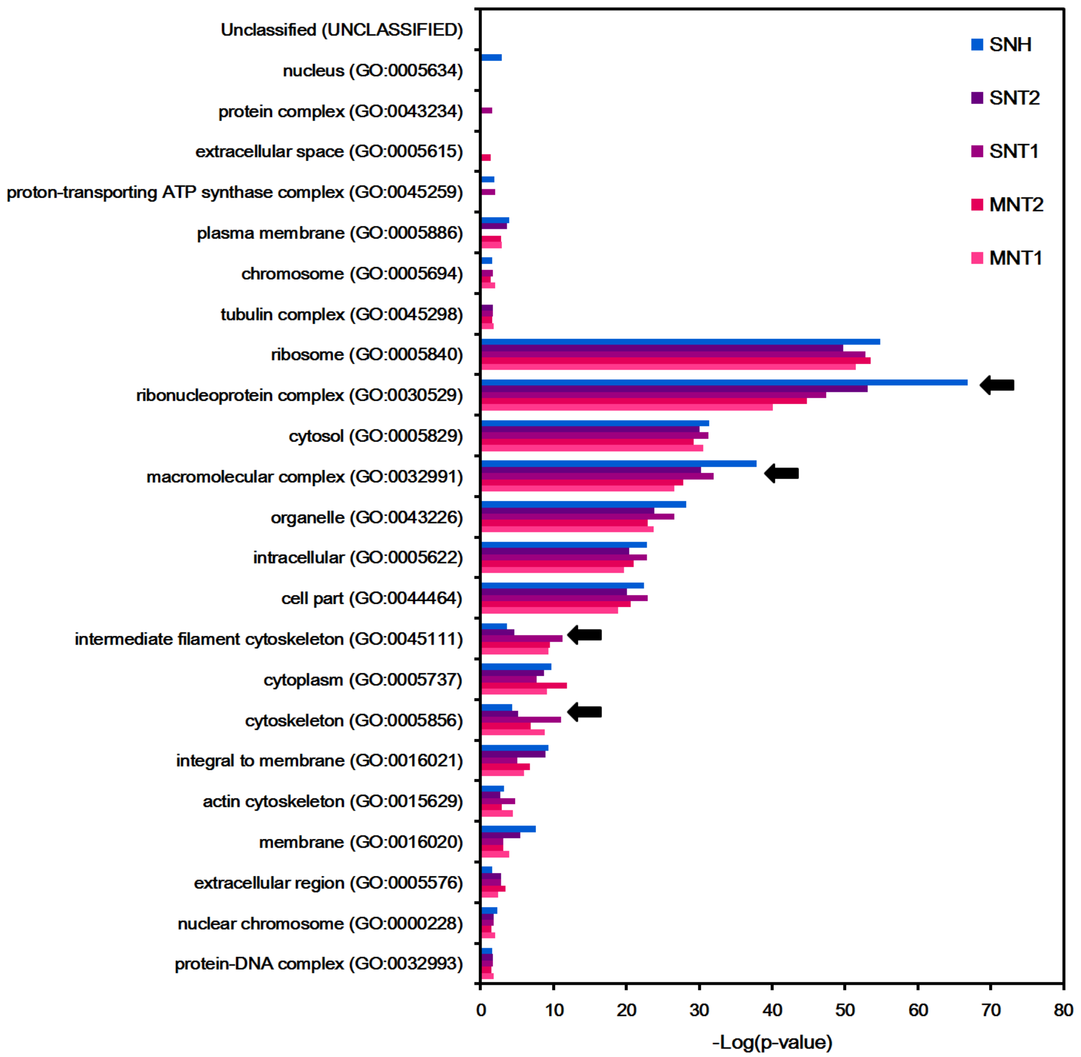

Supplementary Figure.47 Overrepresentation (OR) test of the affinitive proteins based on cellular component (CC) classification. Black arrows showed the obvious distinctions in protein locations for SNH group compared to nanotubes.

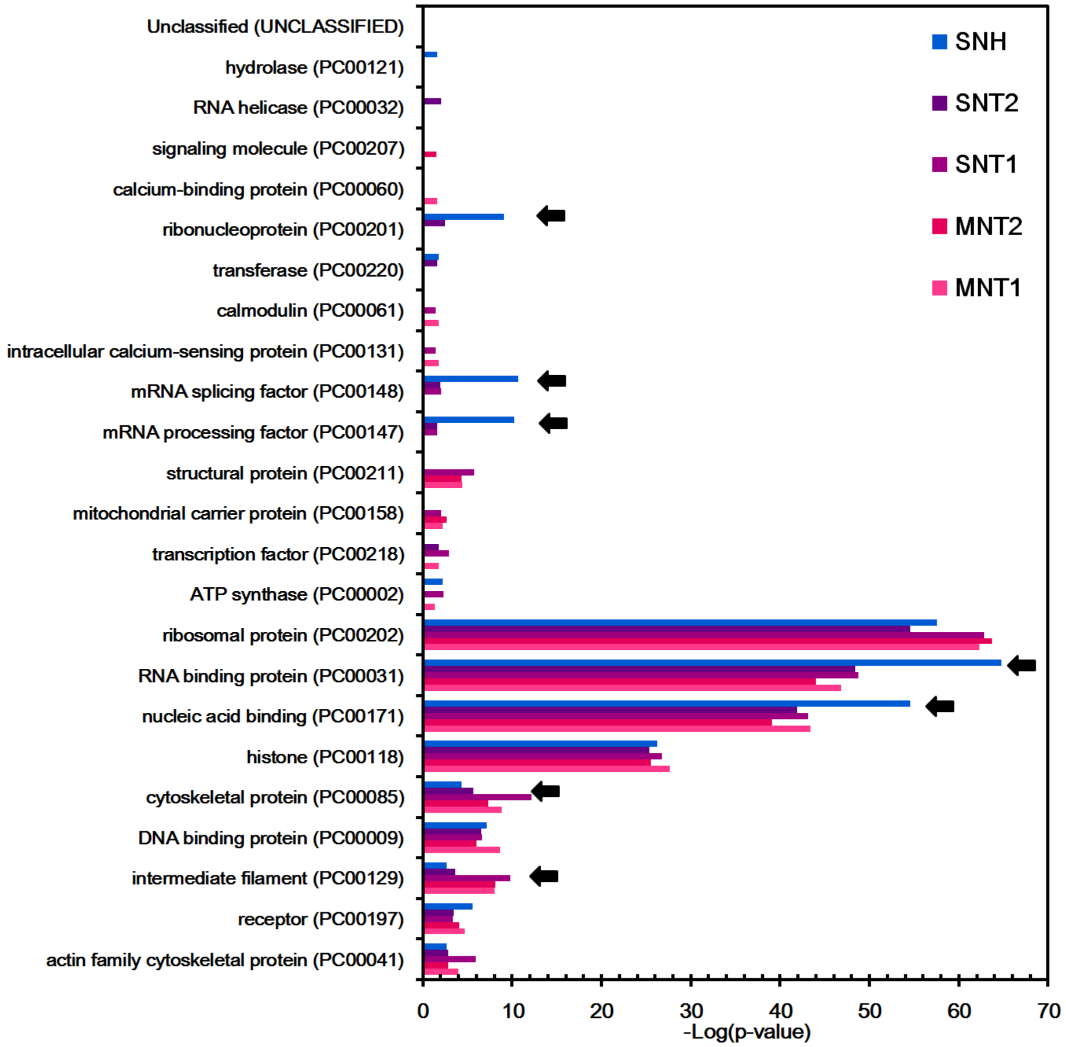

Supplementary Figure.48 Overrepresentation (OR) test of the affinitive proteins based on protein class (PC) classification. Black arrows showed the obvious distinctions in protein functions for SNH group compared to nanotubes.

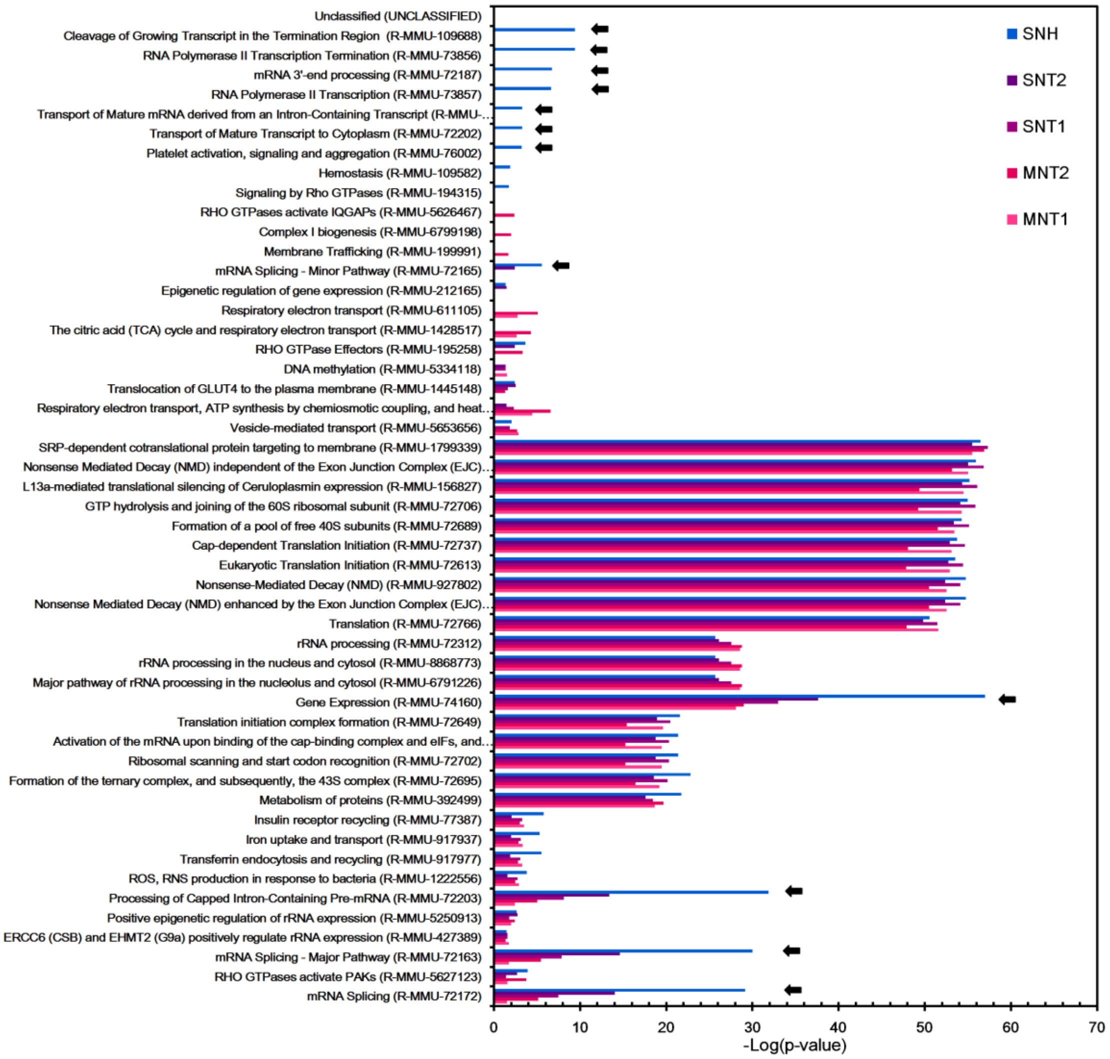

Supplementary Figure.49 Overrepresentation (OR) test of the affinitive proteins based on reactome pathway classification. Black arrows showed the obvious distinctions in protein functions for SNH group compared to nanotubes.

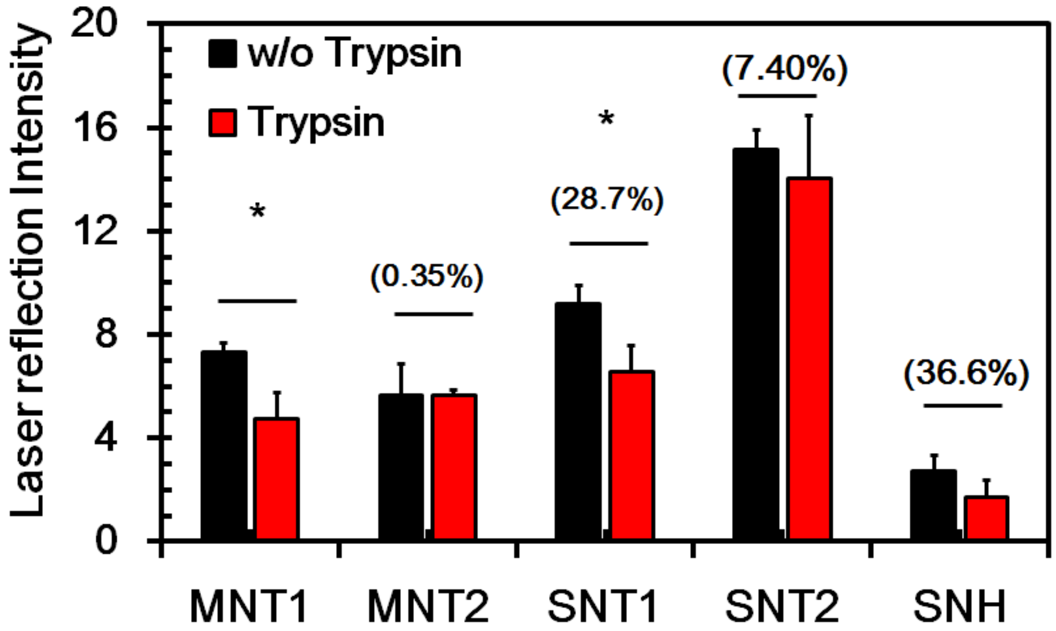

Supplementary Figure.50 Pre-treatment of trypsin decreased the internalizations of nanocarbons detected by LR technology via CLSM. Three independent CLSM detections were performed in study. The mean LR intensities of cells were recorded and compared with no trypsin adding groups for each type of nanocarbons. The values in brackets denoted the reduced percentages of cellular uptake for different nanocarbons. Data were expressed as mean ± s.d. (n=3) and the statistical significances were calculated by Student’st-test. * $p<0.05$.

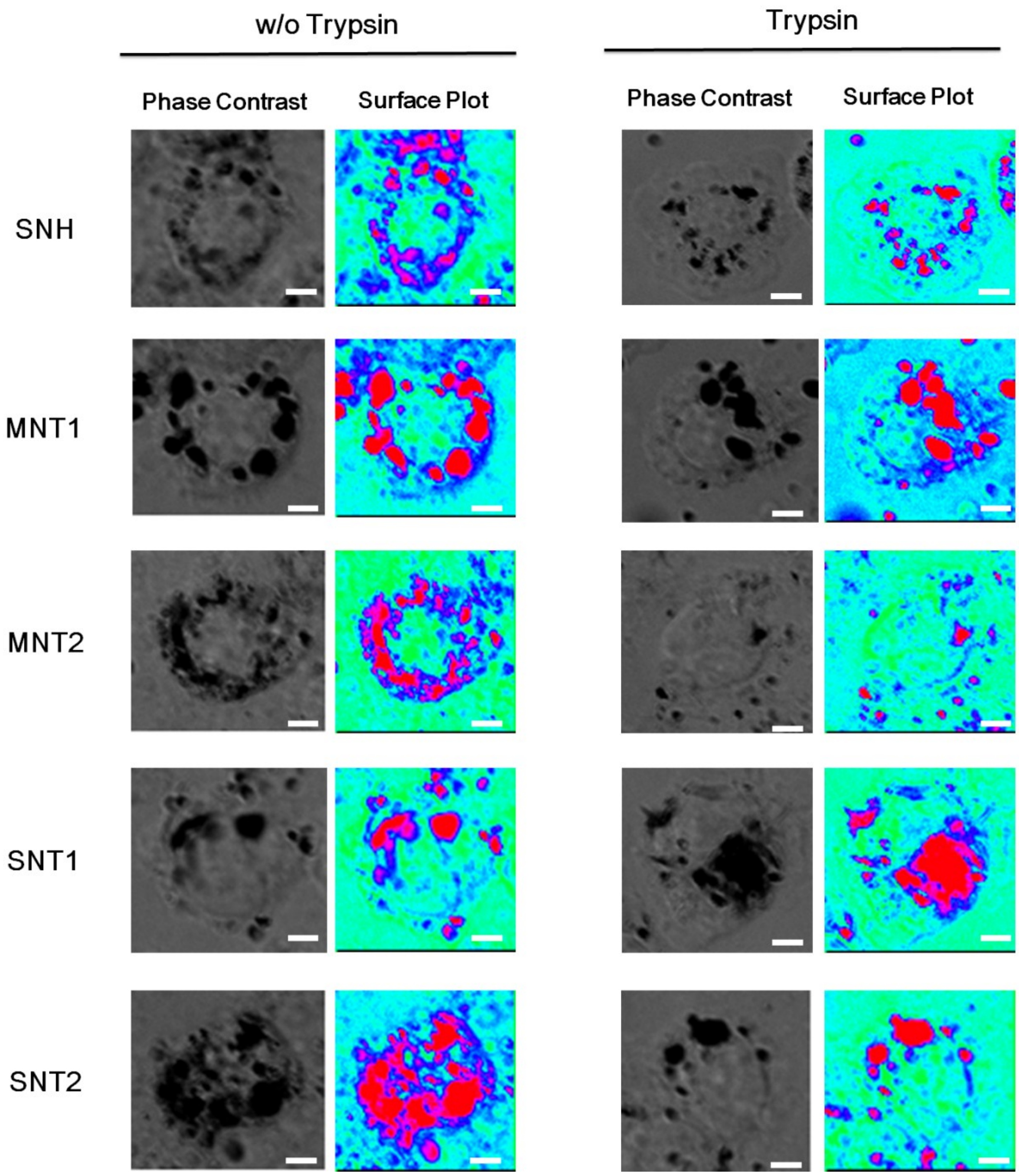

Supplementary Figure.51 Microscopy images of intracellular nanocarbons after trypsin treatment. Scale bar: 5 μm. The surface plots were obtained by IPP software to highlight the intracellular nanocarbons (red color), showing the slight decreases of nanocarbon internalizations caused by trypsin.

Supplementary Table.1 The normalization of cellular uptakes for different nanocarbon incubations

	$\boldsymbol{I}_{\boldsymbol{LR}(\boldsymbol{medium})} = \mathbf{f}_{\mathbf{1}}(\boldsymbol{C}_{(\boldsymbol{medium})})$	$\boldsymbol{I}_{\boldsymbol{LR}(\boldsymbol{cells})} = \mathbf{f}_{\mathbf{2}}(\boldsymbol{C}_{(\boldsymbol{medium})})$	$\boldsymbol{C}_{(\boldsymbol{cells})} = \mathbf{f}_{\mathbf{3}}(\boldsymbol{C}_{(\boldsymbol{medium})})$	$C_{(\text{cells})}$ in the case of nanocarbon incubation with 100 μg ml^{-1}	Incubative concentration regulation for normalized cellular uptake
SNH	I=262.57C -1394.2	I=474.46C +6703.4	C_c =1.81 C_m +30.84	211.54 μg ml^{-1}	100 μg ml^{-1}
MNT1	I=218.42C +1323.6	I=445.14C +22765	C_c =2.04 C_m +98.17	301.97 μg ml^{-1}	55.63 μg ml^{-1}
MNT2	I=198.64C +1944	I=402.31C +20181	C_c =2.03 C_m +41.47	244.00 μg ml^{-1}	83.97 μg ml^{-1}
SNT1	I=92.5C +1618.5	I=348.73C +15793	C_c =3.77 C_m +153.24	530.24 μg ml^{-1}	15.46 μg ml^{-1}
SNT2	I=113.99C +3374.3	I=372.16C +13530	C_c =3.26C_m +89.09	415.58 μg ml^{-1}	37.50 μg ml^{-1}

Supplementary Table.2 Mass ratios of nanocarbons that used for ALM interaction analysis. The converted mass ratios of five nanocarbons could assure the basically identical apparent contact area for five nanocarbons with ALMs

	Fitting based on Cumulants algorithm		Fitting based on NNLS algorithm	
	Ratio of specific surface area (compared to SNH as 1.00)	Mass ratio based on equal surface area (compared to 100 μg SNH)	Ratio of specific surface area (compared to SNH as 1.00)	Mass ratio based on equal surface area (compared to 100 μg SNH)
MNT1	1.35	74	0.93	108
MNT2	0.92	109	0.85	118
SNT1	0.60	166	0.59	170
SNT2	0.19	519	0.22	457
SNH	1.00	100	1.00	100

Supplementary Note 1

The normalization of intracellular nanocarbon concentrations during the cellular incubation

Briefly, the first related issue is the quantitative determination of these nanocarbons inside of cells. We think that the classical fluorescence labeling strategy is not a good choice here because of the influence of chemical modification and possible fluorescence interference from intracellular substances and so on. So a label-free laser reflection (LR) technology based on confocal laser scanning microscopy (CLSM) was utilized in study to evaluate the intracellular nanocarbons. In the manuscript, we had demonstrated that LR signals co-localized well with intracellular nanocarbons (Supplementary Figure.16). Here, our additional tests further demonstrated that there were good correlations between LR signal and nanocarbon concentration in acellular (Supplementary Figure.18) and cellular models (Supplementary Figure.19). For five types of nanocarbons, LR intensities increased with the concentration, and the data regressions all manifested the good linear dependences (Supplementary Figure.18d and 19d). The regression functions in two models could be described as follow:

Acellular model: $I_{\mathrm{LR(medium)}} = \alpha C_{\mathrm{medium}} + \gamma$ (1)

Cellular model: $I_{\mathrm{LR(cells)}} = \beta C_{\mathrm{medium}} + \delta$ (2)

Where α and β represented the slopes of regression lines, γ and δ represented the intercepts. $I_{\mathrm{LR(medium)}}$ was the LR intensities of nanocarbons in medium, while $I_{\mathrm{LR(cells)}}$ was the LR intensities of nanocarbons in cells. C_{medium} was the concentration of nanocarbons in medium.

Notably, as shown in Supplementary Figure.18b and 19b, the LR intensities per unit area for five nanocarbons were nearly invariable, no matter with the changes of concentration or environment (acellular vs cellular models). So it could be rational to deduce that the correlation between LR signals and intracellular nanocarbons conformed to function (1), which was described as follow:

$$I_{\mathrm{LR(cells)}} = \alpha C_{cells} + \gamma \quad (3)$$

According to function (1) to (3), the correlation between C_{medium} and C_{cells} could be obtained based on the linear conversion.

So $$C_{\mathrm{cells}} = \frac{\beta}{\alpha} C_{\mathrm{medium}} + \frac{\delta-\gamma}{\alpha} \quad (4)$$

It was then established the linear correlation of intracellular concentration with extracellular incubative concentration.

Namely, our investigations confirmed the feasibility of our established quantitative

assay of laser refection technology. Then, we found the special incubating concentration for each of the five nanocarbons via uptake study, and under such concentration the five nanocarbons could achieve almost the same level of macrophage uptake (Supplementary Table 1). Namely, we normalized nanocarbon concentrations per cell according to reviewer’s comment.
