# Supplementary material for: Single-walled carbon-nanohorns improve biocompatibility over nanotubes by triggering less protein-initiated pyroptosis and apoptosis in macrophages
Source: Nat Commun. 2018 Jun 19;9:2393. doi: 10.1038/s41467-018-04700-z (PMC6008334; doi:10.1038/s41467-018-04700-z)
Supplement: Supplementary file 2 — Description of Additional Supplementary Files [file 41467_2018_4700_MOESM2_ESM.docx]

**Description of Additional Supplementary Files**

File Name: Supplementary Data 1

Description: LFQ Proteimics identification (Search Result) of total cellular proteins after incubation with five nanocarbons based on identical incubative concentration (100 μg ml-1)

File Name: Supplementary Data 2

Description: LFQ Proteimics identification (Search Result) of total cellular proteins after incubation with five nanocarbons based on identical intracellular concentration

File Name: Supplementary Data 3

Description: LFQ proteomics identification and characterization of high affinitive proteins with five nanocarbons

File Name: Supplementary Data 4

Description: LFQ Proteimics identification (Search Result) of surface binding proteins on five nanocarbons (1st detection)

File Name: Supplementary Data 5

Description: LFQ Proteimics identification (Search Result) of surface binding proteins on five nanocarbons (2nd detection)
